# Interpretable Quantitative Structure–Activity Relationship (QSAR) for identification of potent antifungal activity agents towards *Candida albicans* ATCC 2091

**DOI:** 10.1007/s11030-025-11404-2

**Published:** 2025-11-28

**Authors:** Mariusz Zapadka, Krzysztof Zbigniew Łączkowski, Anna Budzyńska, Mateusz Maciejewski, Przemysław Dekowski, Bogumiła Kupcewicz

**Affiliations:** 1 Department of Inorganic and Analytical Chemistry, Nicolaus Copernicus University in Toruń, Ludwik Rydygier Collegium Medicum in Bydgoszcz, Jurasza 2, 85-089 Bydgoszcz, Poland; 2 Department of Chemical Technology and Pharmaceuticals, Nicolaus Copernicus University in Toruń, Ludwik Rydygier Collegium Medicum in Bydgoszcz, Jurasza 2, 85-089 Bydgoszcz, Poland; 3 Department of Microbiology, Nicolaus Copernicus University in Toruń, Ludwik Rydygier Collegium Medicum in Bydgoszcz, Jurasza 2, 85-089 Bydgoszcz, Poland; 4 Faculty of Mathematics and Computer Science, Nicolaus Copernicus University in Toruń, Chopina 12/18, 87-100 Toruń, Poland; 5 New Technologies Department, Softmaks.pl Sp. z o.o., Kraszewskiego 1, Bydgoszcz, Poland

**Keywords:** Structure–activity relationships, Molecular descriptor, Molecular modeling, RDF(R), GETAWAY

## Abstract

Fungal infections are an increasing global health issue. Despite available treatments, fungal resistance reduces medicine effectiveness. This research conducted QSAR analysis on fifty-one 4-aryl-2-hydrazinothiazole derivatives previously evaluated for antifungal activity. The QSAR model was derived from a hybrid method combining genetic algorithms (GA) and multiple linear regression (MLR). The analysis showed a negative correlation between pMIC and RDF100e, ITH, R4m+, RDF120s, and GATS8e. The model was validated using an external test set by the leave-one-out cross-validation method. Additionally, Y-randomization, MAE, and Golbraikh-Tropsha metrics assessed the model’s applicability domain. The study offers an in-depth molecular descriptor interpretation through three methods: atomic pair distribution, substructure-based analysis, and molecular surface mapping with cumulative atomic contributions. These methods help identify favorable and unfavorable structural groupings. Key molecular features influencing antifungal activity were identified, particularly the spatial arrangement of N1-hydrazine and C4 fragments in the thiazole nucleus. The research highlights Van der Waals interactions, electronegative atoms in substituents, and electron-donating groups. To address the limitations of modeling a small dataset, we applied the novel ARKA approach—based on Arithmetic Residuals in K-groups Analysis—to reduce descriptor dimensionality while preserving chemical relevance and improving interpretability.

**Graphical abstract:**

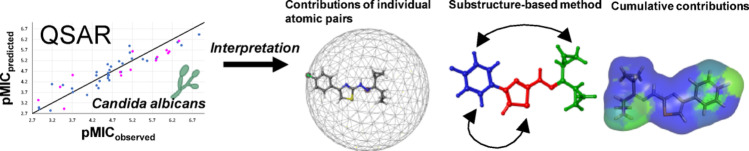

In the era of artificial intelligence (AI), molecular descriptor interpretation remains crucial in QSAR studies, especially for small datasets, where AI methods are less effective. This study identifies key molecular descriptors governing the antifungal activity of thiazole derivatives against *Candida albicans* through QSAR modeling. The findings clarify molecular influences on bioactivity, providing a valuable alternative to AI for predicting compound efficacy.

**Supplementary Information:**

The online version contains supplementary material available at 10.1007/s11030-025-11404-2.

## Introduction

*Candida* spp. are the primary opportunistic fungal pathogens in humans, and the mortality associated with invasive fungal infections of this etiology has steadily increased in recent decades. The most commonly reported species, *C. albicans*, is responsible for various infections localized in mucous membranes and skin (e.g., inflammation of the oral cavity and vagina). It is also an etiological factor in wound infections, including burn wounds, contributing to an increase in mortality rates among patients with thermal injuries [1]. A weakened host immune system, combined with the ability of these microorganisms to adhere to the surface proteins of epithelial cells, which involves, among others, Hyphal Wall Proteins 1 and 2 (Hwp1p and Hwp2p), or the adhesin Eap1p (Enhanced Adherence to Polystyrene), the ability to invade host cells (with the participation of proteinase Sap5p and other hydrolytic enzymes) and to avoid the immune response, may result in the development of not only superficial fungal infections but also deep and systemic ones [2]. C. albicans is one of the more common etiological agents of hospital-acquired bloodstream infections. Apart from C. albicans, most cases of invasive candidiasis are caused by species such as Nakaseomyces glabrata (formerly C. glabrata), C. parapsilosis, C. tropicalis, and C. krusei. Another significant etiological factor is C. auris, listed along with C. albicans under critical priority in the World Health Organization's fungal priority pathogens list (WHO FPPL) [3]. This pathogen, which is difficult to eradicate, causes invasive candidiasis with increased mortality (29%–53% of fatal cases). Prognosis depends not only on the patient's condition but also on the implementation of proper treatment, which is difficult due to this pathogen’s multidrug resistance (including pan-drug-resistant strains) [4, 5].

Patients with AIDS, organ transplant recipients, people with implanted biomaterials, oncology patients undergoing chemotherapy and other immunocompromised individuals are particularly at risk of invasive candidiasis. The treatment of deep fungal infections remains a challenge due to the increasing occurrence of antifungal drug resistance, especially in patients with prolonged exposure to antifungal therapy, those treated with broad-spectrum antibiotics, immunosuppressive drugs, anticancer drugs, and anti-AIDS drugs [6]. The main factors contributing to antimicrobial resistance include the overuse of these drugs, underdosing, poor hygiene and sanitation practices, and limited development of new drugs [1]. Additionally, the need for repeated therapy in patients due to recurring infections involving *Candida* spp. contributes to the rise in isolation rates of drug-resistant fungal strains. In turn, the increased rate of emergence of resistant strains of N. glabrata is influenced by the haploid genome, unlike other *Candida* species [7]. Another significant issue is the ability of *Candida* spp. to form biofilms, which complicates the eradication of microorganisms due to the increase in the minimum inhibitory concentration (MIC) of drugs observed in fungal cells suspended within the biofilm [8, 9].

Candidiasis treatment primarily involves the use of azoles (imidazoles, such as miconazole and ketoconazole, and triazoles, such as fluconazole and voriconazole), polyenes (such as amphotericin B and nystatin), and echinocandins (such as caspofungin and anidulafungin) [10]. Drugs from the first of these classes, which are fungistatic antifungal agents, are widely used in candidemia. However, their use is increasingly limited by the emergence of *Candida* spp. strains with high levels of tolerance to drugs like fluconazole, leading to persistent candidemia and the selection of resistant strains [10, 11]. The diverse pharmacokinetics of azoles further limit their use in certain infections. Side effects, especially with long-term azole use, stem from their hepatotoxicity and inhibition of steroidogenesis [12]. Polyenes, which are fungicidal agents, are characterized by an extensive toxicity profile, thus their use is mainly limited to short-term treatment of local infections or invasive infections caused by strains resistant to echinocandins and azoles [13]. Echinocandins are recommended by the Infectious Diseases Society of America (IDSA) as first-line treatments for invasive candidemia due to their fungicidal action against these yeast-like fungi, broad spectrum, and limited side effects [14]. Reports of acquired resistance to polyenes and echinocandins appear less frequently than with azoles. However, the increase in MIC values and the growing percentage of resistant *Candida* spp. strains, often after prolonged exposure to the drug, pose a significant challenge for clinicians. Resistance to polyenes and echinocandins is primarily noted in species such as *N. glabrata*, *C. krusei*, and *C. auris* (with resistance rates of up to 10%) [10, 15–17].

Due to the increasing resistance to antifungal drugs and the side effects that some of these drugs may have on patients, alternative therapeutic solutions are being sought—in the form of new drugs or synergistic combinations of existing drugs with other compounds. Early and effective antifungal therapy would reduce mortality rates.

Hydrazine thiazole derivatives have attracted growing interest in recent years owing to their potential antimicrobial activities, especially against fungal pathogens that pose important clinical challenges [18]. The action of these types of compounds has been evidenced by numerous investigations, where their activity against *Candida* spp. has been a particular focus [18]. While there is a notable lack of comprehensive studies on QSAR models of hydrazine thiazole derivatives with antimicrobial activity [19], the literature is predominantly focused on the analysis of Structure–Activity Relationships (SAR), exploring correlations between chemical structure and biological activity [20–24]. The QSAR models play an essential role in the predicting biological activity from molecular structures. To fulfill this gap, the present study was designed to develop a QSAR model for hydrazine thiazole derivatives. The research also focused on interpreting the QSAR model by conducting an in-depth molecular descriptor analysis. A relevant lacuna in the current scientific literature is the lack of articles exclusively dedicated to methods of molecular descriptor interpretation within the context of QSAR models. Molecular descriptors assess physicochemical attributes based on molecular structure. They embed important information, inter alia, shape, electronic distribution, hydrophobicity, and hydrogen bonding capability, which could potentially influence biological activity. The molecular descriptors, in conjunction with the machine learning method, establish the nature of the relationship between biological activity and molecular structure, thus enabling the creation of more efficient and specific therapeutic agents. However, the explanation of molecular descriptors remains a highly challenging task in the field of QSAR modeling. One significant limitation arises from the complex and multidimensional nature of many descriptors, which might hide their precise role in modulating biological activity. Also, certain descriptors might not present evident structural analogies, making it challenging to translate numerical values into useful chemical information [25, 26]. This ambiguity is increased when using machine learning algorithms since models often operate as ‘black boxes,’ prioritizing predictive accuracy over interpretability. However, in recent literature, SHAP (SHapley Additive exPlanations) has emerged as a powerful tool for enhancing the interpretability of machine learning predictions. SHAP assigns importance values to individual molecular descriptors, providing insights into their contribution to the biological activity of chemical compounds [27–29]. However, a notable limitation of this method is that its interpretation is conducted independently of the original information, including the mathematical algorithm used to calculate the descriptors and the molecular structure of the chemical compound itself. As a result, the connection between specific chemical groups or physical properties and their influence on biological activity remains unclear. This gap underscores the need for further research to bridge the divide between abstract numerical descriptors and the structural and functional attributes of chemical entities. In this way, researchers can more easily understand how specific molecular features affect activity, enabling the rational design of compounds with desired properties. This area of QSAR research is very relevant but underdeveloped, and it points out the need for more in-depth studies on descriptor interpretation approaches. Another goal of this study was to assess the usefulness of the two ARKA descriptors (ARKA_1 and ARKA_2) in interpreting the QSAR model. The ARKA descriptors effectively encode the chemical information of various descriptors in a particular form of computationally derived descriptors using an (A)rithmetic (R)esiduals in K-groups (A)nalysis approach. ARKA descriptors are particularly recommended by Kunal Roy as an effective dimensionality reduction technique for statistical modeling of small datasets. Their ability to condense complex molecular information into interpretable components makes them especially suitable for QSAR analyses where the number of compounds is limited [30].

## Results and discussion

### Analysis and interpretation of selected QSAR model

Among the developed QSAR models, the optimal one was selected for further analysis using Multi-Criteria Decision Making (MCDM) approach (Sect. 8, Supplementary Information). It demonstrated the best statistical parameters and the lowest RMSEs and MAEs in training, cross-validation, and external prediction sets. The antifungal activity of thiazole derivatives towards *Candida albicans* (ATCC 2091) was described by the linear equation with five parameters (Eq. 1):

1 $$\begin{aligned}&pMIC=4.6771\left(\pm 0.0703\right)-0.5651\left(\pm 0.0814\right)\cdot {\boldsymbol{R}}{\boldsymbol{D}}{\boldsymbol{F}}100{\boldsymbol{e}}\\ &\quad-0.4206\left(\pm 0.0794\right)\cdot {{\boldsymbol{I}}}_{{\boldsymbol{T}}{\boldsymbol{H}}}-0.2905\left(\pm 0.1047\right)\cdot {{\boldsymbol{R}}}_{4}^{+}({\boldsymbol{m}})\\ &\quad-0.2070\left(\pm 0.0878\right)\cdot {\boldsymbol{R}}{\boldsymbol{D}}{\boldsymbol{F}}120{\boldsymbol{s}}-0.1308\left(\pm 0.0739\right)\cdot {\boldsymbol{G}}{\boldsymbol{A}}{\boldsymbol{T}}{\boldsymbol{S}}8{\boldsymbol{e}}\end{aligned}$$

The QSAR models were evaluated using various validation parameters, as presented in Table S3 (Supplementary Information). A detailed discussion of validation metrics, their significance, and their role in assessing the QSAR model can be found in Sect. 3 of the Supplementary Information.

**Fig. 1:**
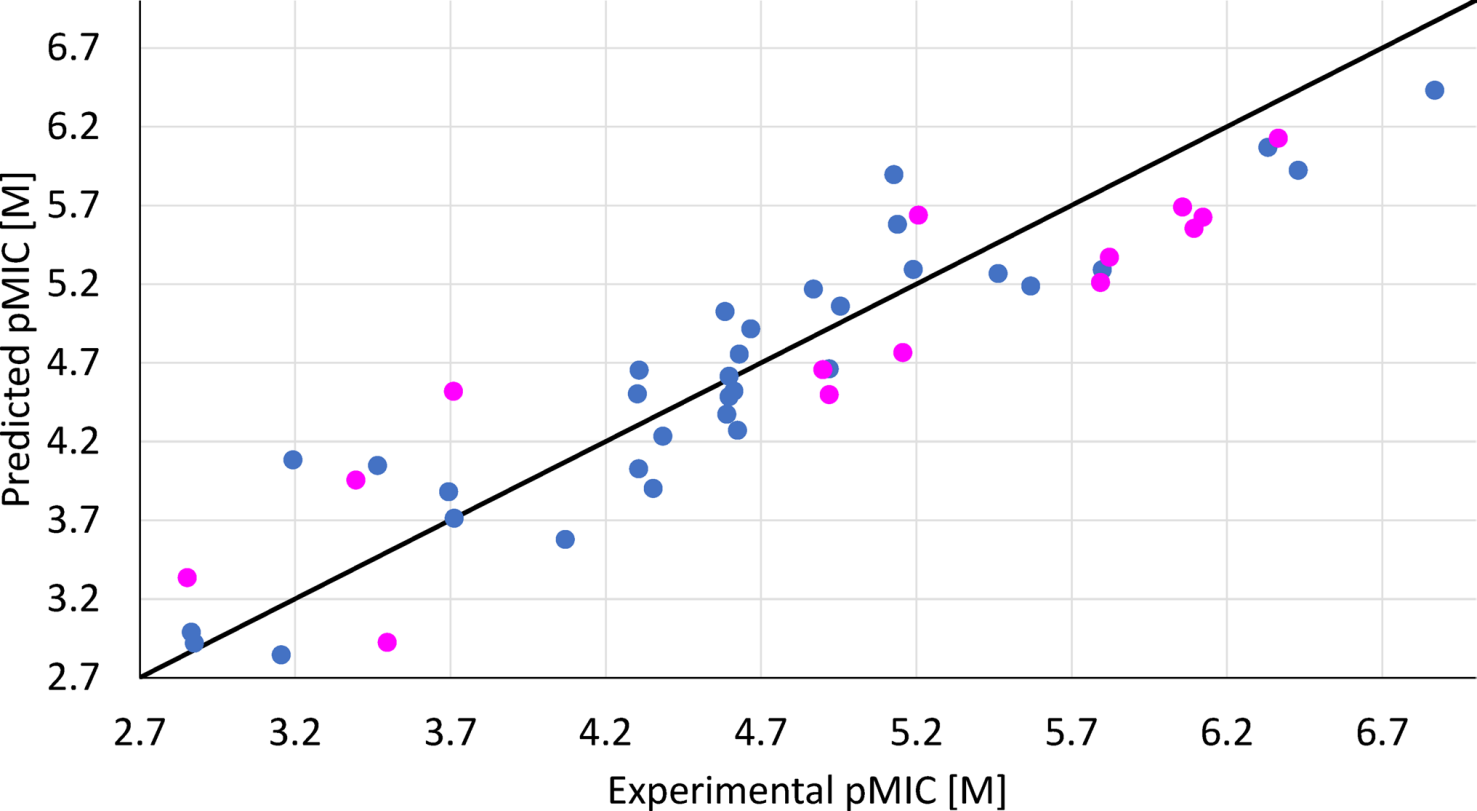
Scatter plot of predicted versus experimentally measured pMIC (M) of antifungal activities against *Candida albicans* (ATCC 2091) of thiazole derivatives (training set—blue points, test set—magenta points)

Figure 1 shows the plot of predicted pMIC against the experimental results.

Regression coefficients represent the effect of one-unit change in an independent variable on the dependent variable, keeping other variables constant. All the significant molecular descriptors have negative coefficients indicating that a decrease in their values enhances antifungal activity (higher pMIC = lower MIC). The descriptors can be ranked by their impact on the dependent variable: RDF100e, I_TH_, ${R}_{4}^{+}\left(m\right)$, RDF120s, GATS8e. Standard deviations of regression coefficients are in parenthesis, and Table 1 provides details on the selected molecular features.

**Table 1:** Molecular descriptors of the MLR model

Name	Description	Class
RDF100e	Radial Distribution Function − 100/weighted by Sanderson electronegativity	RDF descriptors
I_TH_	Total information content on the leverage equality	GETAWAY descriptors
${R}_{4}^{+}\left(m\right)$	R maximal autocorrelation of lag 4/weighted by mass	GETAWAY descriptors
RDF120s	Radial Distribution Function − 120/weighted by I-state	RDF descriptors
GATS8e	Geary autocorrelation of lag 8 weighted by Sanderson electronegativity	2D autocorrelations

### Interpretation of QSAR

The study aims to develop a predictive QSAR model while identifying significant molecular features of thiazole derivatives responsible for antifungal activity against *Candida albicans* (ATCC 2091). This aligns with the fifth OECD, which recommends mechanistic interpretability of QSAR models, if possible. The other four principles—defined endpoint, unambiguous algorithm, defined applicability domain, appropriate measures of goodness-of-fit, robustness, and productivity—have been met as previously demonstrated. Polishchuk has pointed out that the global interpretation of QSAR model presents a dual challenge: ensuring the interpretability of both selected descriptors and the applied machine learning technique [31].

**Fig. 2:**
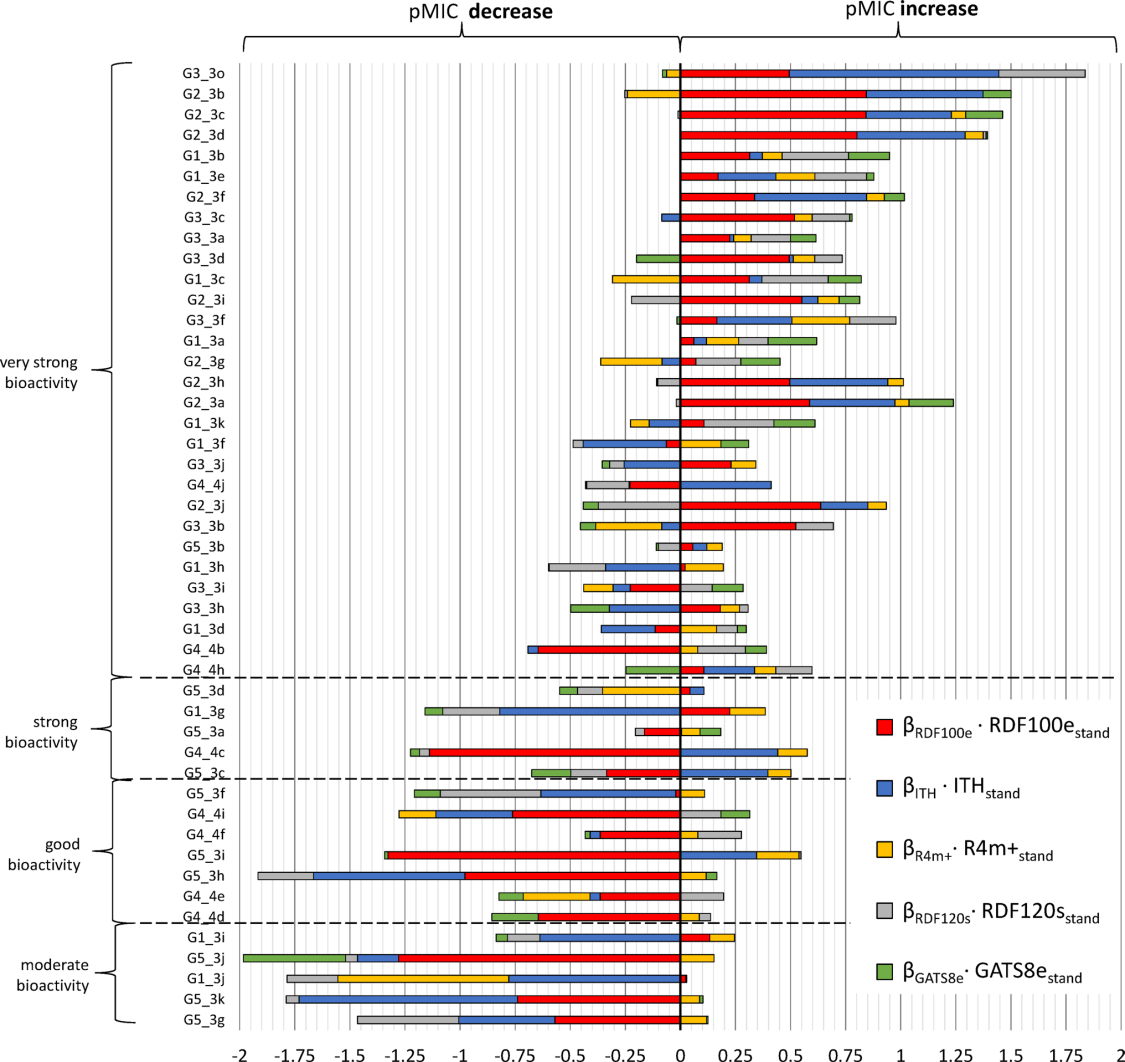
Contribution of individual components of the regression equation on the predicted biological activity for thiazole derivatives

The QSAR model was interpreted in accordance with the model → descriptors → structure paradigm. To comprehensively identify and evaluate how different structural features of molecules encoded by molecular descriptors affect the endpoint, attention should be paid to (1) the data preparation process (standardization of molecular descriptor matrix), (2) sign and value of estimated regression coefficients (${\beta }_{i}$) and (3) methodology for calculating the descriptor. Figure 2 shows the effect of the individual components of the equation on the predicted pMIC value.

The thiazole derivatives are listed in descending order of pMIC values. In addition, compounds were divided into activity classes according to pMIC values. Cumulative bars consist of elements representing the influence of individual molecular descriptors, understood as the product of the standardized descriptor value and the regression coefficient (${\beta }_{i}$), on the pMIC value. Additionally, the molecular descriptors' influences are ranked based on their decreasing ${\beta }_{i}$ values. Standardized descriptors were used to build models. Prior to standardization, all molecular descriptors had positive sign values, referred to as raw values. However, after standardization, descriptor values can be both positive and negative. The influence of a specific descriptor on the pMIC is not only dependent on its value and sign but also on the value and sign of the regression coefficient (${\beta }_{i}$). In Eq. 1, all regression coefficients are negative which means that negative values of standardized molecular descriptors have a positive influence and lead to an increase in pMIC values. Conversely, positive values of standardized molecular descriptors decrease pMIC values.

Understanding the QSAR model based on transformed variables can be facilitated by calculating a raw descriptor value that equals zero after standardization. This value shows whether the descriptor has a positive or negative effect on the modeled endpoint, depending on the regression coefficient. For instance, in Figure S3, the relationship between standardized and raw RDF100e values is shown (${RDF100e}_{stand.}={0.3802\cdot RDF100e}_{raw}-2.041$)). The positive influence occurs for raw RDF100e values less than 5.368, while a negative effect occurs for raw values greater than 5.368.

For the remaining descriptors, their raw values were similarly determined (for I_TH_: 67.069; ${R}_{4}^{+}\left(m\right)$: 0.045; RDF120s: 14.197 and GATS8e: 0.872).

### The interpretation methods of selected molecular descriptors

Most programs for calculating molecular descriptors consider the molecular structure as a single entity. Consequently, researchers lack the ability to analyze the contributions of specific molecular fragments to the descriptor value. To address this issue, we employed a dedicated methodology to determine the contributions of individual atomic pairs and molecular fragments to the descriptors values. The interpretation relies on the principle that molecular descriptors in the developed QSAR model are additive in nature. The interpretation was carried out using three approaches: (1) analysis of the distribution and significance of atomic pairs, (2) the substructure-based method and (3) analysis of the molecular surface mapped with the values of cumulative contributions of individual atoms.

The first interpretation approach involves analyzing the **contributions of individual atomic pairs** (${\gamma }_{ij}$) to the descriptor value. The final value of the additive molecular descriptor is the sum of contributions (${\gamma }_{ij}$) for the atomic pairs (*i*,* j*) (Eq. 2):

2 $${Molecular\;Descriptor}_{additive}=\sum_{i,j\epsilon \mathcal{M},i<j}{\gamma }_{ij},$$

where $i, j$—*i*th and *j*th atoms constitute elements of the molecular structure ($\mathcal{M}$) of compound, $\mathcal{M}$—the arrangement of the specifically numbered atoms that comprise a molecule.

The inequality ($i<j$) ensures that the contributions for pairs of atoms are counted uniquely, thereby preventing any duplication in the total contributions.

In the second **substructure-based method**, the molecule is divided into molecular fragments. The final value of molecular descriptor is the sum of the total intra-fragment contribution—${\varepsilon }_{t}$ and the total inter-fragment contribution between fragments—${\delta }_{t}$:

3 $${Molecular\;Descriptor}_{additive}={\varepsilon }_{t}+{\delta }_{t}.$$

**Scheme 1:**
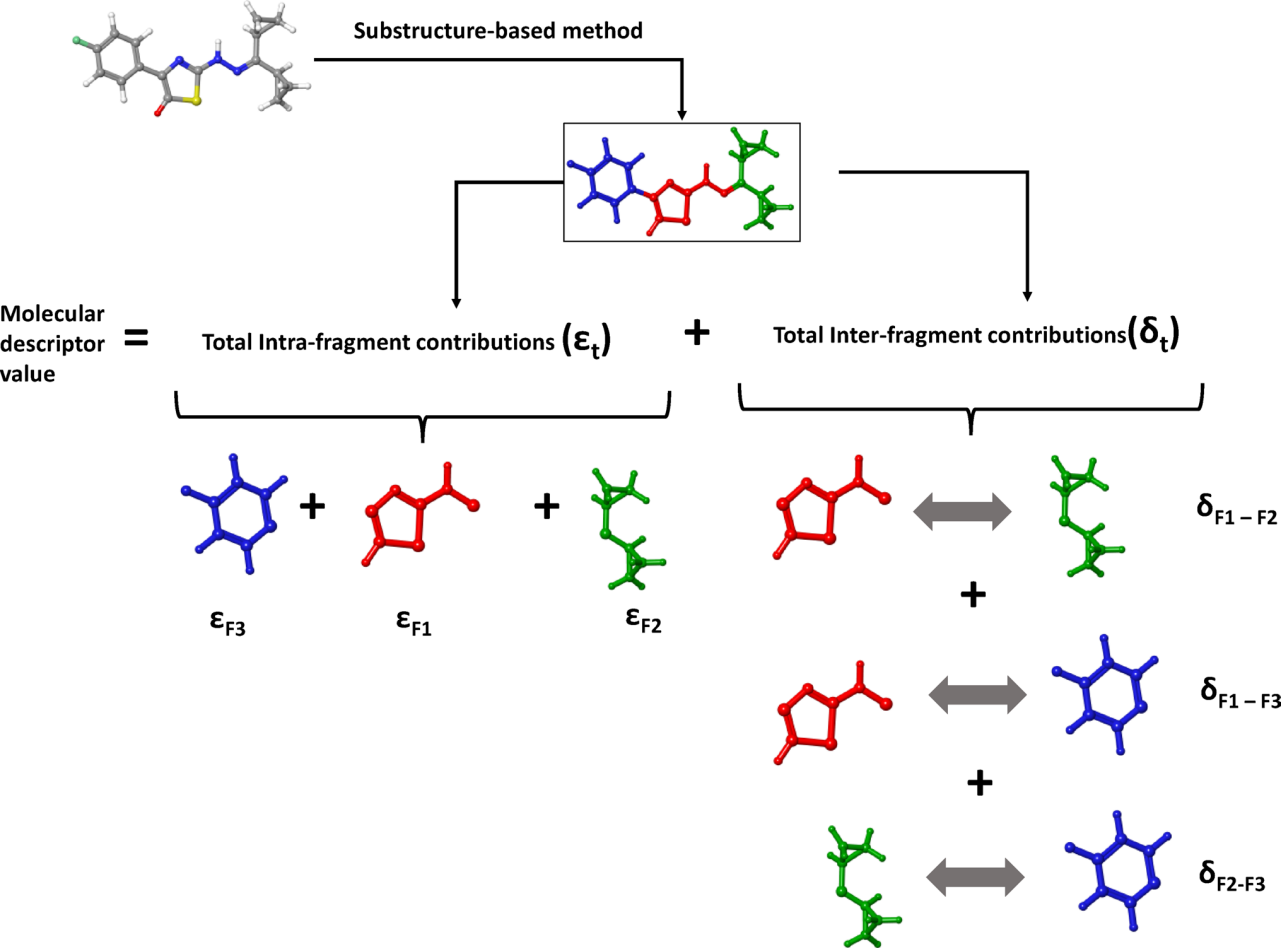
Visual explanation of the substructure-based approach. The molecular descriptors as the sum of the individual fragment’s contributions: intra- and inter-fragment effects

It is a very convenient way of defining the contribution not only of each fragment (${\varepsilon }_{t}$) but also to demonstrating the importance of their mutual spatial relationships (${\delta }_{t}$) in the molecular descriptor value (Scheme 1).

**Scheme 2:**
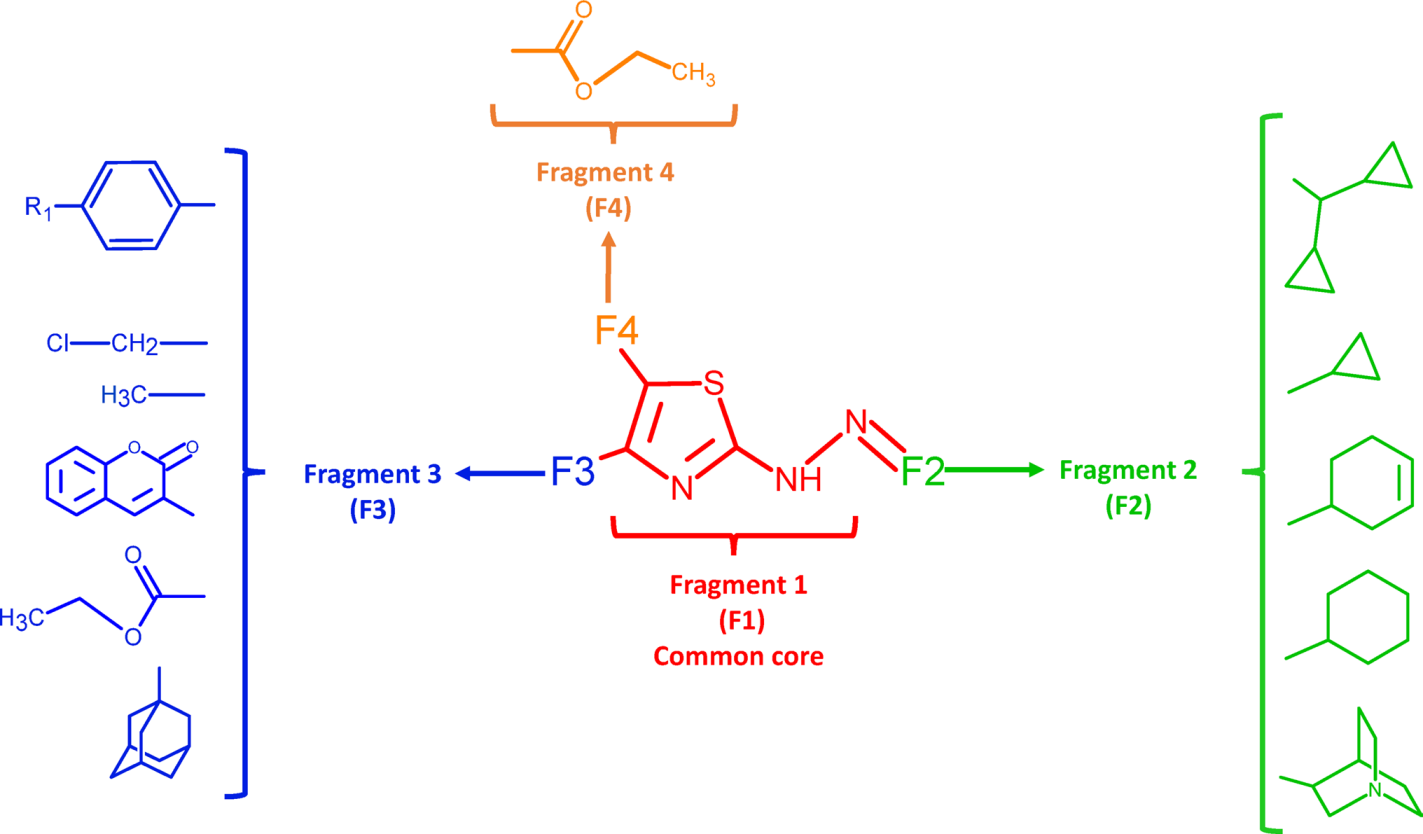
The studied thiazole derivatives were divided into four substructures: the first fragment, also referred to as the common core (F1, see Fig. 1), the second fragment (F2), the third fragment (F3), and the fourth fragment (F4). For a better overview, molecular fragments were highlighted in different colors: F1—red, F2—green, F3—blue, F4—orange

This substructure-based method is used as a screening tool for the identification of intra- and inter-fragment effects that are favorable for the increase of the molecular descriptor value. The molecular structures of the studied thiazole derivatives were decomposed into four non-overlapping substructures. The initial fragment (F1) is red, the next fragment (F2) is green, the following fragment (F3) is blue, and the final substructure (F4) is orange (Scheme 2).

The total value of the intra-fragment contribution (${\varepsilon }_{t}$) can be defined as the sum of the contributions of the individual structural fragments —${\varepsilon }_{Fi}$ (Eq. 4). In the case of thiazole derivatives, four intra-fragment contributions can be distinguished—one for each isolated structural fragment.

4 $${\varepsilon }_{t}=\sum {\varepsilon }_{\mathrm{Fi}}={\varepsilon }_{F1}+{\varepsilon }_{F2}+{\varepsilon }_{F3}+{\varepsilon }_{F4},$$

where Fi—the subset of atoms of the molecule ($\mathcal{M}$) constituting the *i*th molecular fragment. ${\varepsilon }_{\mathrm{Fi}}$—intra-fragment contribution of *i*th molecular fragment (Fi), ${\varepsilon }_{\mathrm{F}1}$—intra-fragment contribution of common core (F1), ${\varepsilon }_{F2}$—intra-fragment contribution of the second fragment (F2), ${\varepsilon }_{F3}$—intra-fragment contribution of the third fragment (F3), ${\varepsilon }_{F4}$—intra-fragment contribution of the fourth fragment (F4).

The individual intra-fragment contributions can be represented as the sum of the atomic pair contributions from the corresponding structural fragments (Eqs. 5–8):

5 $${\varepsilon }_{F1}=\sum_{i,j\in F1, i<j }{\gamma }_{ij},$$

6 $${\varepsilon }_{F2}=\sum_{i,j\in F2, i<j }{\gamma }_{ij},$$

7 $${\varepsilon }_{F3}=\sum_{i,j\in F3, i<j}{\gamma }_{ij},$$

8 $${\varepsilon }_{F4}=\sum_{i,j\in F4, i<j }{\gamma }_{ij}.$$

Equation 5 is the sum of the contributions (${\gamma }_{ij}$) for the atomic pairs (*i*,* j*) from the common core substructure (F1). Equation 6 is similar but calculates the sum of atomic pair contributions in the second fragment (F2). Equations 7 and 8 follow the same idea but for the third (F3) and fourth (F4) fragments, respectively.

Analogous equations can be presented for inter-fragment contributions (Eq. 9). The total inter-fragment contribution (${\delta }_{t}$) is the sum of the contributions resulting from the mutual spatial arrangement of fragments. For four non-overlapping substructures (F1, F2, F3, F4), we can create six possible inter-fragment contributions:

9 $$\begin{aligned}{\delta }_{t}&={\sum {\delta }_{\mathrm{Fi}-\mathrm{Fj}}=\delta }_{\mathrm{F}1-\mathrm{F}2}+{\delta }_{\mathrm{F}1-\mathrm{F}3}+{\delta }_{\mathrm{F}1-\mathrm{F}4}\\ &\quad+{\delta }_{\mathrm{F}2-\mathrm{F}3}+{\delta }_{\mathrm{F}2-\mathrm{F}4}+{\delta }_{\mathrm{F}3-\mathrm{F}4}.\end{aligned}$$

For example, the value of ${\delta }_{\mathrm{F}1-\mathrm{F}2}$ can indicate how important the mutual arrangement of the common core (F1) with fragment F2 is for the descriptor value.

Each inter-fragment contribution can be represented as the sum of the contributions (${\gamma }_{ij}$) for atomic pairs (*i*,* j*), where the *i*th atom belongs to one fragment and the *j*th atom is in another fragment, but *i*th and *j*th atoms are not the same (Eqs. 10–15):

10 $${\delta }_{\mathrm{F}1-\mathrm{F}2}=\sum_{\left(i,j\right)\in F1\times F2 }{\gamma }_{ij},$$

11 $${\delta }_{\mathrm{F}1-\mathrm{F}3}=\sum_{(i,j)\in F1\times F3 }{\gamma }_{ij},$$

12 $${\delta }_{\mathrm{F}1-\mathrm{F}4}=\sum_{(i,j)\in F1\times F4 }{\gamma }_{ij},$$

13 $${\delta }_{\mathrm{F}2-\mathrm{F}3}=\sum_{(i,j)\in F2\times F3}{\gamma }_{ij},$$

14 $${\delta }_{\mathrm{F}2-\mathrm{F}4}=\sum_{(i,j)\in F2\times F4 }{\gamma }_{ij},$$

15 $${\delta }_{\mathrm{F}3-\mathrm{F}4}=\sum_{(i,j)\in F3\times F4 }{\gamma }_{ij}.$$

For example, contribution ${\delta }_{\mathrm{F}1-\mathrm{F}2}$ refers to two non-empty sets, F1 and F2, representing substructures. The Cartesian product $F1\times F2$ is the set of all ordered pairs of elements (atoms) from F1 and F2, i.e.,

16 $$F1\times F2=\left\{\left(i,j\right):i\in F1, j\in F2\right\}.$$

To generate unique pairs, each atom in set F1 should be systematically combined with every atom in set F2. This approach will yield a complete set of pairs that includes all possible combinations of the two molecular fragments.

In the third approach, **the cumulative contributions (**${{\boldsymbol{\Omega}}}_{{\boldsymbol{n}}}$**)** are utilized to map the solvent-accessible surface area (SASA) of molecules. The ${\Omega }_{n}$ value for the *n*th atom is determined by the sum of the atomic pair’s contributions (${\gamma }_{ij}$) that include the *n*th atom (Eq. 17):

17 $${\Omega }_{n} =\sum_{i\ne n }{\gamma }_{in}={\gamma }_{1n}+{\gamma }_{2n}+\dots +{\gamma }_{n-1,n}+{\gamma }_{n+1,n}+\dots ,$$

The ${\Omega }_{n}$ contributions are visualized on the SASA surface of chemical compounds using a sequential color scale i.e., blue to brown. ${\Omega }_{n}$ values are color-coded: brown for the highest, yellow for high, green for middle, and blue for the lowest.

Scheme S2 presents three proposed methods for interpreting additive molecular descriptors of a thiazole derivative and RDF100(e). Plot (a) shows atomic pair contributions, identifying interactions at the atomic level. Plot (b) shows the results of substructure-based method analysis, facilitating a detailed examination of the contributions made by individual molecular fragments. Meanwhile, plot (c) depicts SASA-mapped molecular surfaces, which provide a global perspective. SASA-mapped molecular surfaces present several advantages, including an intuitive understanding of molecular structure, the capacity for effectively comparing structural elements, and insights at a global level concerning the entire molecule. Conversely, alternative approaches tend to offer more detailed local insights, facilitating a more precise analysis of specific structural moieties.

### Interpretation of RDF100e descriptor

The methodology for calculating RDF100e is detailed in Sect. 4.1. The lower the standardized value of RDF100e, the higher the predicted pMIC value, indicating greater antifungal activity against *Candida albicans* ATCC 2091. The interpretation of a molecular descriptor translates numerical values into structural features based on its raw values. An increase in pMIC values is associated with RDF100e raw values less than 5.368 (Fig. S3). The RDF100e values of the thiazole derivatives were decomposed into intra- and inter-fragment contributions according to the substructural approach (Fig. S4). Thiazole derivatives are divided into molecular fragments in Scheme 2. The value of the RDF100e descriptor results from the sum of the contributions of atomic pairs (${\gamma }_{ij}$), the atoms of which are situated roughly 10 Å (R, radius of the spherical volume). This distance is adequate for connecting the atoms of solely two separate structural fragments. Hence, the RDF100e descriptor depicts only the positioning of fragments F3, F2, F4 and the core (F1) within the three-dimensional space. Intra-fragment contributions (${\varepsilon }_{Fi}$) are irrelevant to the descriptor value as their values are equal to 0.

Compounds with a raw RDF100e descriptor value of less than 5.368 are mainly considered to be molecules with very high activity. Most G1-G3 compounds have a raw RDF100e descriptor value below 5.368 (${\beta }_{RDF100e}\cdot {RDF100e}_{stand}>0$). In contrast, most thiazole derivatives of groups 3 and 4 have raw RDF100e values above 5.368. For most compounds, regardless of the activity group, the RDF100e value depends mainly on the ${\delta }_{\mathrm{F}3-\mathrm{F}2}$ contributions encoding the mutual arrangement of fragments 3 and 2 (red bar). The ${\delta }_{\mathrm{F}3-\mathrm{F}1}$ and ${\delta }_{\mathrm{F}2-\mathrm{F}1}$ contributions also influence the descriptor value.

Figure S5 illustrates the outcomes of substructural analysis carried out on the raw RDF100e descriptor value, which has been segregated into individual groups of thiazole derivatives (G1–G5). It can be observed that the RDF100e value of compounds belonging to groups 1 and 2 is solely dependent on the arrangement of fragments 3 and 2 and 3 and the common core in the three-dimensional space, which correspond to contributions ${\delta }_{\mathrm{F}3-\mathrm{F}2}$ and ${\delta }_{\mathrm{F}3-\mathrm{F}1}$, respectively. Compared to G2 compounds, thiazole G1 derivatives are characterized by larger ${\delta }_{\mathrm{F}3-\mathrm{F}2}$ contributions and smaller ${\delta }_{\mathrm{F}3-\mathrm{F}1}$. The increase in the value of ${\delta }_{\mathrm{F}3-\mathrm{F}2}$ contributions result mainly from the different structures of fragment 2 of compounds G1 and G2. The F2 fragment of G2 thiazole derivatives includes a cyclopropane moiety, while G1 compounds feature two cyclopropane groups. Therefore, G2 structures have more atomic pairs separated by approximately 10 Å, which increases the value of ${\delta }_{\mathrm{F}3-\mathrm{F}2}$ contributions. It should be noted that in the case of compounds falling under the groups G3 and G4, the ${\delta }_{\mathrm{F}2-\mathrm{F}1}$ contributions hold significant importance, as they encode the mutual geometric relationships between the cyclohex-4-ene moiety (G3), cyclohexane (G4), and the common core. The substructure-based method and the weighting scheme analysis have limitations in comprehensively elucidating the extent of variation in the ${\delta }_{\mathrm{F}3-\mathrm{F}1}$ contribution values of halogen derivatives. Table 2 lists the RDF100e values of halogenated thiazole derivatives.

**Table 2:** Summary of RDF100e values for halogenated thiazole derivatives

F		Cl		Br	
Name	RDF100e	Name	RDF100e	Name	RDF100e
G1_3a	5.086	G1_3b	3.913	G1_3c	3.901
G2_3a	2.642	G2_3c	1.449	G2_3b	1.441
G3_3a	4.317	G3_3c	2.955	G3_3b	2.932
G4_4b	8.376	G4_4f	7.074	G4_4e	7.074
G5_3a	6.130	G5_3b	5.107	G5_3d	5.168

In the tested compounds, the halogen atom is substituted in the para position of the phenyl ring (fragment F3). It would be expected that, according to the increase in the mass of the halogen atom, molecules with the Br atom should have the lowest descriptor value, intermediate ones with the Cl atom, and the highest with the F atom. The RDF100e values for bromo- or chlorine derivatives are very similar and smaller compared to fluoroderivatives of thiazoles. The distribution and significance of atomic pair analysis was used to elucidate the RDF100e values of halogenated thiazole derivatives (Fig. S6). The Supplementary Materials include the structures of the analyzed compounds along with the numbering of atoms (Fig. S7). According to Figure S6, it is apparent that the fluorine atom (F37) forms an atomic pair (F37-N1) that strongly affects the descriptor value. The contribution of ${\gamma }_{F37-N1}$ in RDF100e is the highest among all the examined ${\gamma }_{ij}$ contributions, with the consistent value of 1.2 for all the groups of thiazole derivatives (G1–G5). Furthermore, the ${\gamma }_{F37-N1}$ accounts for 100% of the inter-fragment contribution value ${\delta }_{\mathrm{F}3-\mathrm{F}1}$. However, when examining the thiazole bromo derivative (G1_3c), the bromine atom in the para position of the phenyl ring (fragment F3) does not affect the RDF100e value. The chlorine atom in the para position of the phenyl ring does not significantly contribute to the descriptor value. Moving down the halogen group increases the atomic radius and consequently, the length of the C-X bond (where X is the halogen atom). The interatomic distances (${r}_{ij}$) between the chlorine and bromine atoms increase significantly. In contrast, the fluorine atom in the para position of the phenyl ring has an interatomic distance (${r}_{F37-N1}$) = 9.94 Å) is close to the radius of the spherical volume (R) of approximately 10 Å. However, the interatomic distances ${r}_{Cl37-N1}$ and ${r}_{Br37-N1}$ are 10.35 Å and 10.51 Å, respectively. This implies that the differences between R and ${r}_{Cl37-N1}$ and R and ${r}_{Br37-N1}$ are significantly large, leading to the values of the contributions ${\gamma }_{Cl37-N1}$ and ${\gamma }_{Br37-N1}$ being close to zero (as depicted in Equation S1).

The descriptor RDF100e can be effectively interpreted by examining cumulative contribution values (${\Omega }_{n}$) mapped onto the accessible surface area (SASA). Equation 17 describes calculation methodology for ${\Omega }_{n}$. A color scale is used to represent these cumulative contribution values, ranging from brown (the highest ${\Omega }_{n}$ values) followed by yellow (high values), green (middle values), and blue (the lowest values). Figure S8 shows a graphical representation of cumulative contribution values (${\Omega }_{n}$) mapped onto the accessible surface area (SASA) of halogen derivatives of thiazoles with key structural fragments highlighted with red ellipses for clarity.

Examining the molecular surfaces showed green areas (with specific values) only in halogenated thiazole derivatives and were linked to the F37 and N1 atoms. In addition, analyzing the data in Figure S8 can provide additional information that complements the results from the substructural method. For the G1 group of compounds, the RDF100e value is influenced by the spatial arrangement of the cyclopropyl group of the F2 fragment and the phenyl ring of the F3 fragment. Within the G2 compounds, the average Ω contributions are from the cyclopropyl group (F2) and the hydrogen atom (H16 in G2_3a; H19 in G2_3b and G2_3c) of the phenyl ring (F3). In the G3 group, the average ${\Omega }_{n}$ contributions come from the cyclohex-4-ene moiety and the common core hydrogen atom H15 (F1). Furthermore, the analysis of derivatives of the G4 group shows that RDF100e values depend on the spatial relations between the cyclohexane fragment (F2) and the C9 and H16 atoms of the phenyl ring (F3) as well as the C5 and N4 atoms of the common core (F1). Lastly, the values of G5 group compounds are affected by the spatial relations of atoms H29, H31 (high ${\Omega }_{n}$ contributions) of the quinuclidine fragment (F2) and atoms H16 and H19 of the phenyl ring of the F3 fragment.

Figure S9 contains molecular surfaces that show the cumulative contributions (${\Omega }_{n}$) of thiazole derivatives (G1–G5) with –CH3, –CN and –NO2 groups as the R1 substituent of the F3 fragment. The substitution of the phenyl ring with a methyl group at the 4-position of the phenyl ring has a negligible effect on the RDF100e value (${\Omega }_{n}$≈0). However, substituting the phenyl ring in position 4 with nitrile and nitro groups causes an increase in the RDF100e value. The presence of the nitrile group is reflected in the average values of the ${\Omega }_{n}$ contributions of the N31 (F3) and N2, N1 (F2) atoms. On the other hand, the introduction of the nitro group is represented by the average values of the ${\Omega }_{n}$ contributions of the nitrogen atoms of the –NO2 group. The oxygen atoms of this functional group have a negligible effect on the RDF100e value (${\Omega }_{n}$≈0).

The influence of the arrangement of the carbon skeleton in space on the RDF100e value was presented in the example of two selected thiazole derivatives: the chlorine derivative G4_4f and the methyl derivative G4_4h (Fig. S10). Both molecules differ only in the type of R1 substituent, which does not directly affect the RDF100e value. Therefore, differences in descriptor values should be attributed to the spatial arrangement of the carbon skeleton. Compound G4_4f has a higher RDF100e value than G4_4h, 7.074 and 4.872, respectively. In Fig. S10 we compared the 3D geometry of G4_4f (green) and G4_4 (red) by superposition of their structures. The greatest differences in the geometry of the compounds can be observed in the cyclohexane fragment (F2) where the atoms overlap the least. As a result, small changes in the spatial arrangement of the carbon skeleton significantly affect variations in the RDF100e value.

### Interpretation of RDF120s descriptor

The standardized RDF120s value is inversely proportional to the predicted pMIC value. This implies that lower RDF120s values correspond to higher antifungal activity against *Candida albicans* ATCC 2091. An increase in pMIC values is observed when RDF100e raw values are less than 14.197, while a decrease is associated with values exceeding 14.197. The cumulative contributions mapped on the SASA surface were used to interpret the RDF120s descriptor, which is analogous to the RDF100e descriptor. However, RDF120s has a larger sphere radius (R) by 2 Å. As a result, RDF120s only encodes the spatial relationships between fragments F2 and F3 (${\delta }_{\mathrm{F}2-\mathrm{F}3}$). Additionally, RDF120s utilizes the I-State weighting scheme, which combines both electronic and topological characteristics of atoms [32]. Figure S11 displays the SASA surface mapping for the 10 compounds with the highest RDF120s value and G3_3o, the most active molecule with the lowest descriptor value. The substitution of the phenyl ring of the F3 fragment with R1 substituents, such as –NHCOCH3, –CF3, –NO2, –NHSO2CH3, –NHCOCH2Cl, and N3, results in high RDF120s values. This is attributed to the high values of the internal state of atoms: = 0, –F, –Cl, and =N– (as presented in Table S6). The internal state value (s) directly correlates with the number of valence electrons available for intermolecular interactions. Therefore, a higher internal state value leads to a higher number of valence electrons, resulting in stronger intermolecular interactions. Conversely, the low RDF120s value of the G3_3o compound is solely due to the contribution of one atomic pair ${\gamma }_{H30-H22}$, whose weight quotient is 1.

### Interpretation of ITH descriptor

The I_TH_ descriptor is the second most important molecular descriptor of QSAR model. Its calculation methodology is elucidated in Sect. 4.2 (Supplementary Information). As with the previous descriptors, a lower standardized I_TH_ value corresponds to a higher pMIC value. The I_TH_ formula (Eq. S5) comprises two terms: ${A}_{0}\cdot {log}_{2}{A}_{0}$ and $\sum_{g=1}^{G}{N}_{g}\cdot {log}_{2}{N}_{g}$. Figure S12 summarizes the I_TH_ values of the tested compounds considering both components. The blue bars represent the values of the I_TH_ descriptor, while the grey ones mark the second part of Equation S5$\sum_{g=1}^{G}{N}_{g}\cdot {log}_{2}{N}_{g}$), which determines the degree of spatial order of the compounds. In order to determine the values of the first element, it is necessary to sum up the two bars (blue and grey). The element ${A}_{0}\cdot {log}_{2}{A}_{0}$ measures the complexity of the molecular structure. The minimum and maximum values of the raw I_TH_ descriptor are 48.603 and 86.337, respectively, while the average value is 67.453. Figure S12 is a summary of the I_TH_ values of the thiazole derivatives and their classification into activity classes. The compounds are sorted in descending order of pMIC value. The dashed line refers to the raw descriptor value, which indicates the positive and negative effects of I_TH_ on the pMIC value.

The I_TH_ value of thiazole derivatives is greatly influenced by the term ${A}_{0}\cdot {log}_{2}{A}_{0}$. As the number of atoms (${A}_{0}$) increases, the value of ${A}_{0}\cdot {log}_{2}{A}_{0}$ increases, which in turn leads to a higher I_TH_ value. However, the second part of Eq. S5, $\sum_{g=1}^{G}{N}_{g}\cdot {log}_{2}{N}_{g}$, becomes more significant when the molecule contains several atoms with the same leverage value (${h}_{ii}$). It is noteworthy that more than 63% of the compounds that exhibit very strong activity possess descriptor values less than 67.069, indicating a positive impact (${\beta }_{{I}_{TH}}\cdot {{I}_{TH}}_{stand.}>0$) on the dependent variable. Moreover, among the top 10 compounds with the highest pMIC value, only one structure (G3_3c) has an I_TH_ value greater than 67.069. Notably, all thiazole derivatives belonging to the G2 group have an I_TH_ value less than 67.069. However, approximately 70% of thiazoles with strong, good, and moderate antifungal activity against *Candida albicans* ATCC 2091 exhibit negative ${\beta }_{{I}_{TH}}\cdot {{I}_{TH}}_{stand.}$ values.

Figure S13 shows two selected thiazole derivatives, G3_3o and G5_3g. The compound G3_3o, exhibiting the highest pMIC value, demonstrates the lowest I_TH_ value among all thiazole derivatives, while G5_3g, with the lowest pMIC value, has an I_TH_ of 75.567. The atoms contributing to the value of $\sum_{g=1}^{G}{N}_{g}\cdot {log}_{2}{N}_{g}$ are highlighted in red in the molecular structures of these compounds. The higher I_TH_ value of G5_3g, relative to G3_3o, is attributed to two factors: (1) a larger number of atoms (excluding hydrogen atoms, ${A}_{0}$), and (2) a larger number of atoms with influence indicators satisfying the relationship ${h}_{ii}$-${h}_{jj}$≅0.

### Interpretation of ${{\boldsymbol{R}}}_{4}^{+}\left({\boldsymbol{m}}\right)$ descriptor

QSAR model comprises a molecular descriptor known as ${R}_{4}^{+}\left(m\right)$, whose calculation methodology is outlined in Sect. 4.3 (Supplementary Information). The interpretation of this descriptor entails the identification of the atomic pair with the highest contribution to the ${R}_{4}(m)$ value (Eqs. S6 and S7). The regression coefficient shows an inverse relationship between ${R}_{4}^{+}\left(m\right)$ and pMIC. However, the strength of this relationship is very weak, as indicated by the low value of the Pearson correlation coefficient. Consequently, the interpretation of ${R}_{4}^{+}\left(m\right)$ aims to identify trends in the incidence of specific atomic pairs among thiazole derivatives. By analyzing the correlation between standardized and raw ${R}_{4}^{+}\left(m\right)$ values, the trend function's zero was found to be 0.0454. As a result, compounds with descriptor values below 0.0454 demonstrate positive influences of ${R}_{4}^{+}\left(m\right)$ on the pMIC value, whereas compounds with descriptor values above 0.0454 demonstrate negative influences.

Table 3 presents a detailed analysis of the atomic pairs of thiazole derivatives along with their corresponding ${R}_{4}^{+}\left(m\right)$ values. The table also highlights atomic pairs or molecules with ${R}_{4}^{+}\left(m\right)$ values that exceed the criterion (0.0454) in bold. The compounds in the table are listed in descending order of pMIC value.

**Table 3:** A list of thiazole derivatives with their respective atomic pairs and corresponding ${R}_{4}^{+}\left(m\right)$ values

Compound	Atomic pair	${R}_{4}^{+}\left(m\right)$	Compound	Atomic pair	${R}_{4}^{+}\left(m\right)$	Compound	Atomic pair	${R}_{4}^{+}\left(m\right)$
**G3_3o**	**C9-S6**	**0.052**	G3_3f	C9-S7	0.017	G5_3a	C9-S7	0.036
**G2_3b**	**Br30-C8**	**0.071**	G1_3f	C9-S7	0.025	G5_3c	C9-S7	0.034
G2_3c	C9-S7	0.038	**G1_3k**	**Cl37-C9**	**0.054**	**G5_3d**	**Br30-C8**	**0.083**
G2_3d	C9-S7	0.036	G2_3j	C9-S7	0.036	G5_3f	C9-S7	0.033
G1_3b	Cl37-C8	0.035	G3_3j	C9-S7	0.033	G4_4f	C9-S7	0.036
G1_3e	C9-S7	0.026	G4_4j	C9-S6	0.045	**G4_4i**	**F30-F29**	**0.063**
G2_3f	C9-S7	0.036	G1_3d	C9-S7	0.027	G5_3i	C36-O33	0.024
G3_3a	C9-S7	0.036	G1_3h	C9-S7	0.026	G4_4d	C9-S7	0.036
G3_3c	C9-S7	0.036	**G3_3b**	**Br30-C8**	**0.077**	**G4_4e**	**Br30-C8**	**0.077**
G3_3d	C9-S7	0.034	G3_3h	C9-S7	0.036	G5_3h	C9-S7	0.032
**G1_3c**	**Br30-C8**	**0.078**	**G3_3i**	**F37-F36**	**0.059**	G1_3i	Cl47-C37	0.033
G2_3i	C9-S7	0.034	G4_4b	C9-S7	0.036	G5_3j	C31-S6	0.028
G1_3a	C9-S7	0.029	G4_4h	C9-S7	0.034	**G1_3j**	**Cl44-O38**	**0.130**
G2_3a	C9-S7	0.038	G5_3b	Cl37-C8	0.037	G5_3g	C9-S7	0.032
**G2_3g**	**F30-F29**	**0.075**	G1_3g	F39-C9	0.027	G5_3k	C31-S6	0.035
G2_3h	C9-S7	0.037	G4_4c	C9-S7	0.030			

It's worth mentioning that eleven compounds have descriptor values above 0.0454, which indicates a negative impact of the descriptor (${\beta }_{{I}_{TH}}\cdot {{I}_{{R}_{4}^{+}\left(m\right)}}_{stand.}<0$) on the dependent variable. For clarification, we have included a graphical representation of the atom pairs that constitute the descriptor value for these compounds in Figure S14. Additionally, the atomic pair (C9-S7) that occurred most frequently among compounds with a positive impact on the pMIC value was taken into consideration. It has been observed that the substitution of the phenyl ring with fluorine atoms in positions 2 and 4 leads to a decrease in activity (G2_3g; G3_3i; G4_4i). Similarly, when a bromine atom is introduced in position 4 of the phenyl ring, it also leads to a decrease in the dependent variable (G2_3b; G1_3c; G3_3b; G5_3d; G4_4e). Furthermore, the presence of chlorine atoms in the meta position of the phenyl ring of the 1,3-disubstituted compound (G1_3k) leads to a decreased pMIC.

### Interpretation of GATS8e descriptor

GATS8e is the final descriptor in the QSAR model and represents the two-dimensional molecular structure of thiazole derivatives. Its calculation methodology is explained in Sect. 4.4 (Supplementary Information). Like the other descriptors, lower GATS8e values correspond to higher predicted pMIC values. A threshold of 0.872 was also determined to indicate whether GATS8e's influence on the pMIC value is positive or negative (${\beta }_{GATS8e}\cdot {GATS8e}_{stand.}$).

GATS8e is a tool designed to measure spatial autocorrelation specifically for two-dimensional structures. This tool evaluates the clustering degree of atoms sharing similar electronegativity values, separated by a topological distance of eight. Raw GATS8e values below one denote positive spatial autocorrelation, whereas values above one signify negative spatial autocorrelation regarding electronegativity. Figure S15 displays the GATS8e values of thiazole derivatives, arranged in descending order of pMIC value and classified into activity classes. The dashed line in the figure shows the raw descriptor value and indicates whether GATS8e has a positive or negative effect on the pMIC. It is noteworthy that almost 62% of compounds with very strong activity exhibit a descriptor value below 0.872, indicated a positive impact (${\beta }_{GATS8e}\cdot {GATS8e}_{stand.}>0$) on pMIC. In turn, about 66% of thiazoles with good, strong, and moderate antifungal activity against *Candida albicans* ATCC 2091 have negative values of ${\beta }_{GATS8e}\cdot {\mathrm{GATS}8\mathrm{e}}_{stand.}$. Most compounds, where atoms with ${d}_{ij}$ equal to 8 have nearly identical electronegativity values: ${\left({w}_{i}-{w}_{j}\right)}^{2}\cong 0,$ show positive spatial autocorrelation. In Figure S15, twelve compounds with negative spatial autocorrelation are marked with bordered bars. Only four compounds show very strong activity (G3_3d; G2_3j; G3_3b; G3_3h). The negative spatial autocorrelation is associated with certain R1 substituents in the aromatic ring of the F3 fragment of thiazole derivatives, such as –CH_3_, –NO_2_, –CN, –Br, –CF_3_, –NHCO(CH_2_)_2_Cl. Table S7 presents the squares of electronegativity differences for the atoms that constitute thiazole derivatives. Atomic pairs with similar electronegativity values are highlighted in green, ${\left({w}_{i}-{w}_{j}\right)}^{2}\cong 0$, indicating positive autocorrelation and, thus, a GATS8e value of less than 0.872. However, the remaining atomic pairs exhibit negative autocorrelation.

The influence of electronegativity on the GATS8e value was presented in the example of three selected compounds: G1_3a, G1_3b, and G1_3c. The compounds differ in the type of halogen atom in the para position of the phenyl ring (F3 fragment). Specifically, G1_3a contains a fluorine atom, G1_3b contains a chlorine atom, and G1_3c contains a bromine atom. The compounds have the same number of atoms in the molecule (A = 37) and the number of atoms separated by a distance ${d}_{ij}$ equal to 8 (${\Delta }_{k}$=43), but they differ in the weight values (${w}_{i}$) of fluorine atoms, chlorine, and bromine. In particular, the weight value (${w}_{i}$) of the fluorine atom is the highest due to its higher electronegativity, followed by the chlorine atom with an intermediate value and the bromine atom with the lowest value. Figure S16 shows the squares of differences in electronegativity values ${\left({w}_{i}-{w}_{j}\right)}^{2}$ of atomic pairs with a value ${d}_{ij}$ equal to 8 of selected three compounds G1_3a, G1_3b, and G1_3c. Atomic pairs are arranged in descending order of value ${\left({w}_{i}-{w}_{j}\right)}^{2}$.

The negative spatial autocorrelation of G5_3j can be attributed to 21 out of 65 atomic pairs displaying significant differences in their electronegativity values, as indicated by the red border in Figure S17. These distinguished atomic pairs connect the F2 and F3 fragments with F1. In summary, most of the atomic pairs with significant electronegativity differences occur between the atoms of fragment 3 (–CH_3_, –NO_2_, –CN, –Br, –CF_3_, –NHCO(CH_2_)_2_Cl) and fragment 1. Additionally, the presence of quinuclidine (F2) significantly increases the GATS8e.

### ARKA (arithmetic residuals in K-groups analysis) method

We recognize that the limited dataset—comprising 33 compounds modeled with five descriptors—poses a significant limitation. This constraint makes it difficult to interpret descriptors with weaker, yet still statistically significant, correlations to antifungal activity. Acknowledging the inherent challenges of QSAR modeling with small datasets, we addressed this issue by using novel supervised dimensionality reduction techniques (the ARKA framework) to optimize the descriptor matrix while maintaining chemical relevance. The ARKA descriptors encode various chemical information into a specific form of computationally derived descriptors using an (A)rithmetic (R)esiduals in K-groups (A)nalysis approach [30].

**Fig. 3:**
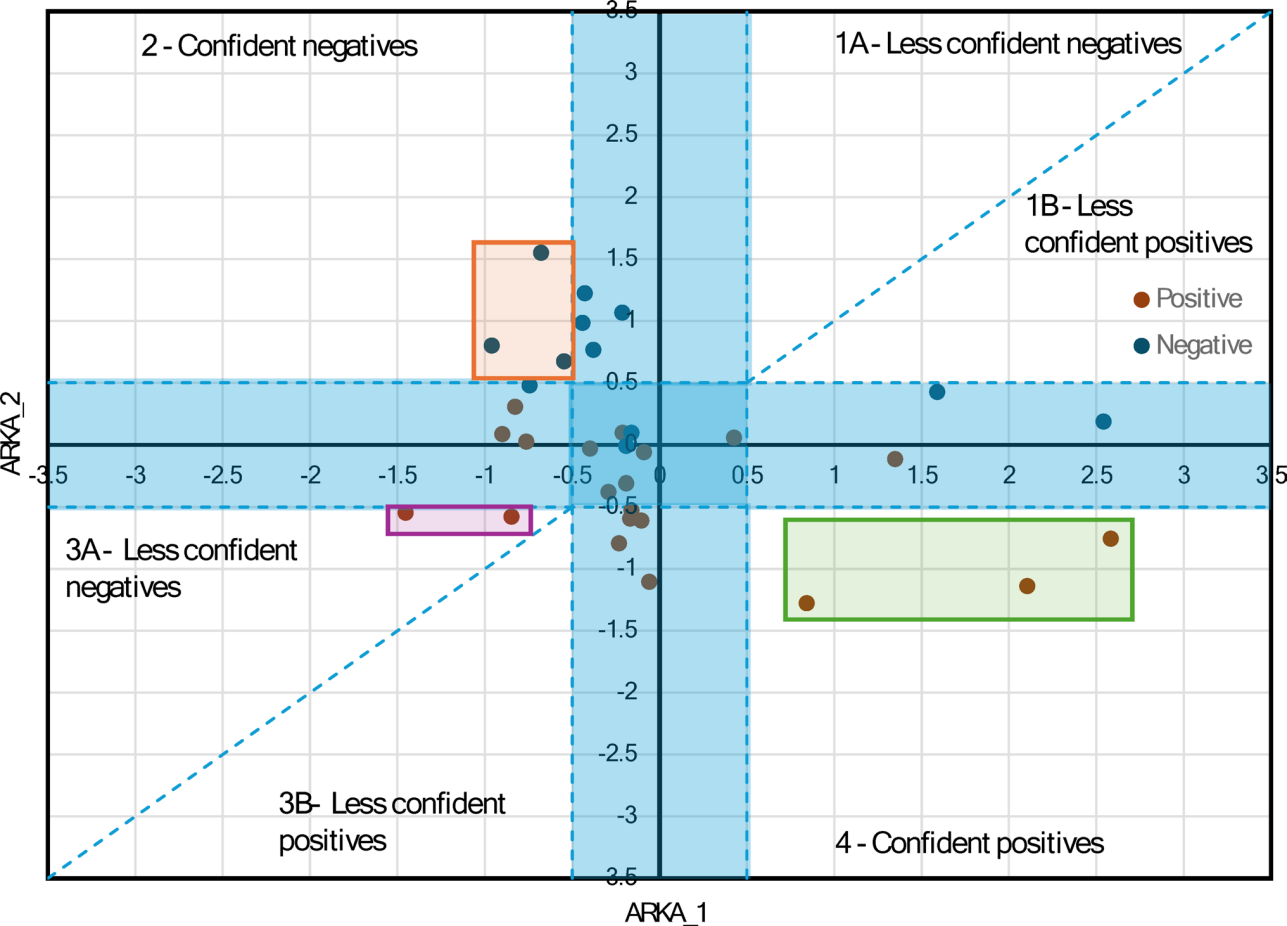
ARKA 2 vs ARKA 1 in the **training set**. The blue-shaded region on the plot highlights the area where both ARKA_1 and ARKA_2 values are less than or equal to 0.5. This zone represents compounds with low descriptor intensity, often associated with reduced classification confidence

**Fig. 4:**
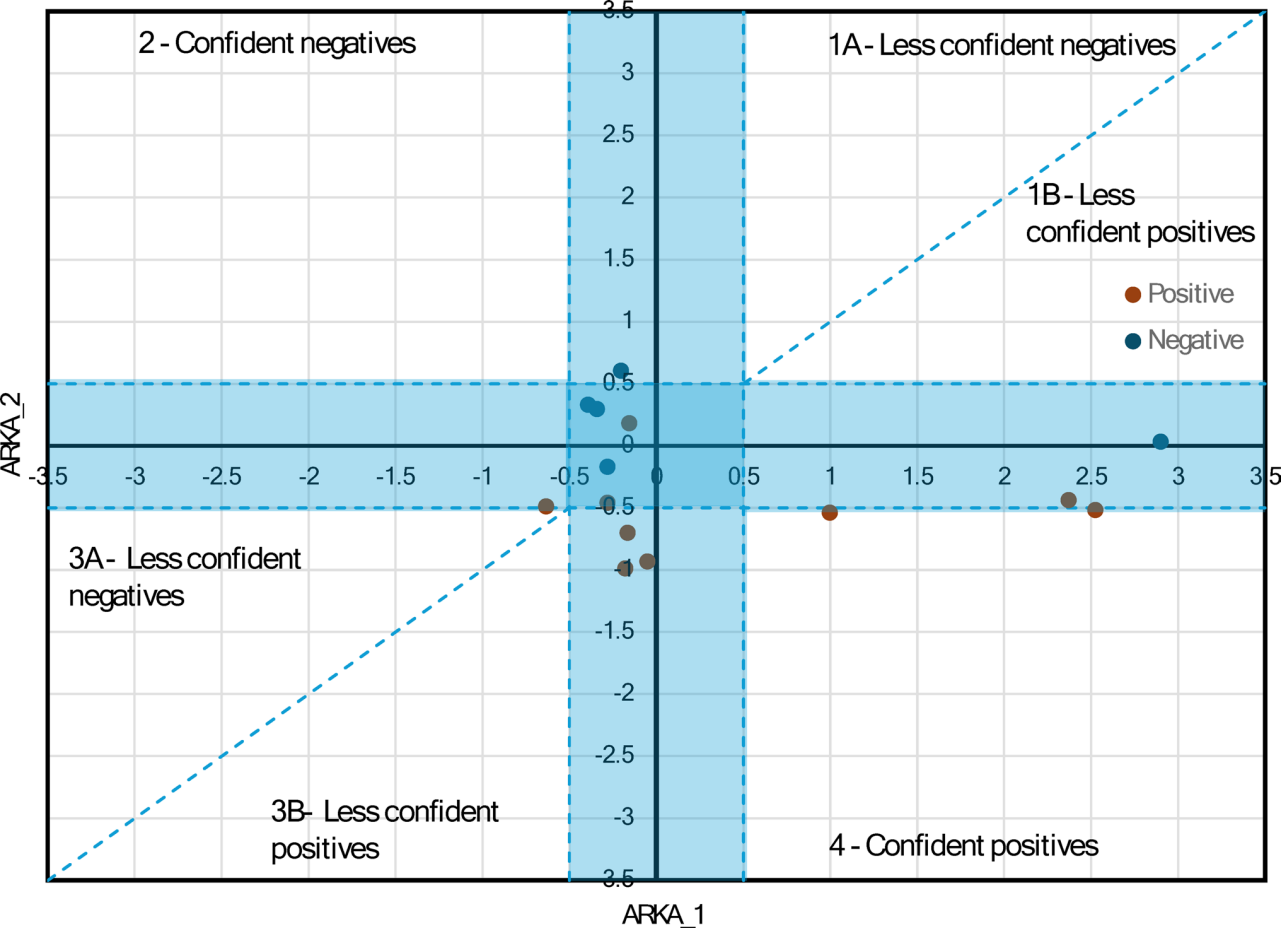
ARKA 2 vs ARKA 1 in the **test set**. The blue-shaded region on the plot highlights the area where both ARKA_1 and ARKA_2 values are less than or equal to 0.5. This zone represents compounds with low descriptor intensity, often associated with reduced classification confidence

Figures 3 and 4 present a scatter plot of ARKA_1 versus ARKA_2, which was used to examine how the compounds are spatially distributed and to highlight regions where slight shifts in ARKA descriptor values may correspond with notable differences in biological activity, suggesting the presence of potential activity cliffs. Since the AKRA descriptor was used in the activity cliff analysis of the classification model, this study focused on the regression model. Therefore, we divided the target (pMIC) in the training set into two groups: those with pMIC greater than 4.46, labeled as the “positive group” and those with pMIC less than 4.46, labeled as the “negative group.” This division corresponds to the previously established classification of thiazole derivatives into four levels of bioactivity: very strong, strong, good, and moderate. In the context of ARKA descriptors, the “positive group” includes only compounds with very strong antifungal activity, while the “negative group” consists of all remaining derivatives with strong, good, or moderate activity levels. Both ARKA_1 and ARKA_2 descriptors were computed based on five key molecular descriptors identified in the developed QSAR model, including RDF100e, ITH, RDF120s, GATS8e, ${{\boldsymbol{R}}}_{4}^{+}\left({\boldsymbol{m}}\right)$.

According to the theory presented by Banerjee and Roy [30], the descriptor ARKA_1 encodes chemical information of the descriptors that have a higher discriminatory capacity toward the positive/active class. Similarly, the descriptor ARKA_2 encodes chemical information of the descriptors with a higher discriminatory ability for the negative/inactive class. The active compounds would most likely be present in the “4—confident positives” region, where ARKA_1 is positive and ARKA_2 is negative. Conversely, the “2—confident negatives” region, where ARKA_1 is negative and ARKA_2 is positive, is typically associated with inactive compounds. In our training set, a clear spatial separation is observed between positive and negative thiazole derivatives in the ARKA_2 vs. ARKA_1 scatter plot (Fig. 3).

Importantly, none of the compounds deviated from these expected regions; therefore, no potential candidates for activity cliffs were identified. The absence of such deviations confirms the robustness of the classification and suggests that minor structural changes did not lead to sudden shifts in biological activity within the dataset.

Two compounds (G3_3f and G1_3e) appear in the “3A—less confident negatives” region, marked by a purple square. Their ARKA_2 values slightly exceed the 0.5 threshold, indicating borderline classification and reduced confidence. Three compounds from the positive group (G3_3o, G2_3b, G1_3c) are positioned in the “4—confident positives” region, marked by a green square. In this case, the ARKA_1 descriptor solely represents the chemical data of the ${{\boldsymbol{R}}}_{4}^{+}\left({\boldsymbol{m}}\right)$ variable, demonstrating a strong discriminatory capacity and playing a key role in distinguishing active compounds within this subset.

A corresponding “2—confident negatives” region, marked with an orange square, includes three correctly classified inactive compounds (G5_3j, G5_3i, and G4_4c). Here, the ARKA_2 descriptor captures chemical information from four key variables: RDF100e, RDF120s, ITH, and GATS8e, which together help differentiate compounds within this subset.

In the test set, the ARKA_2 vs. ARKA_1 scatter plot reveals zones where the model’s predictions may be less reliable. Compounds located near the origin—where both ARKA values are close to zero—display low descriptor intensity, which leads to lower classification confidence due to their proximity to the axes. These points indicate areas of uncertainty in the model’s predictive landscape.

**Fig. 5:**
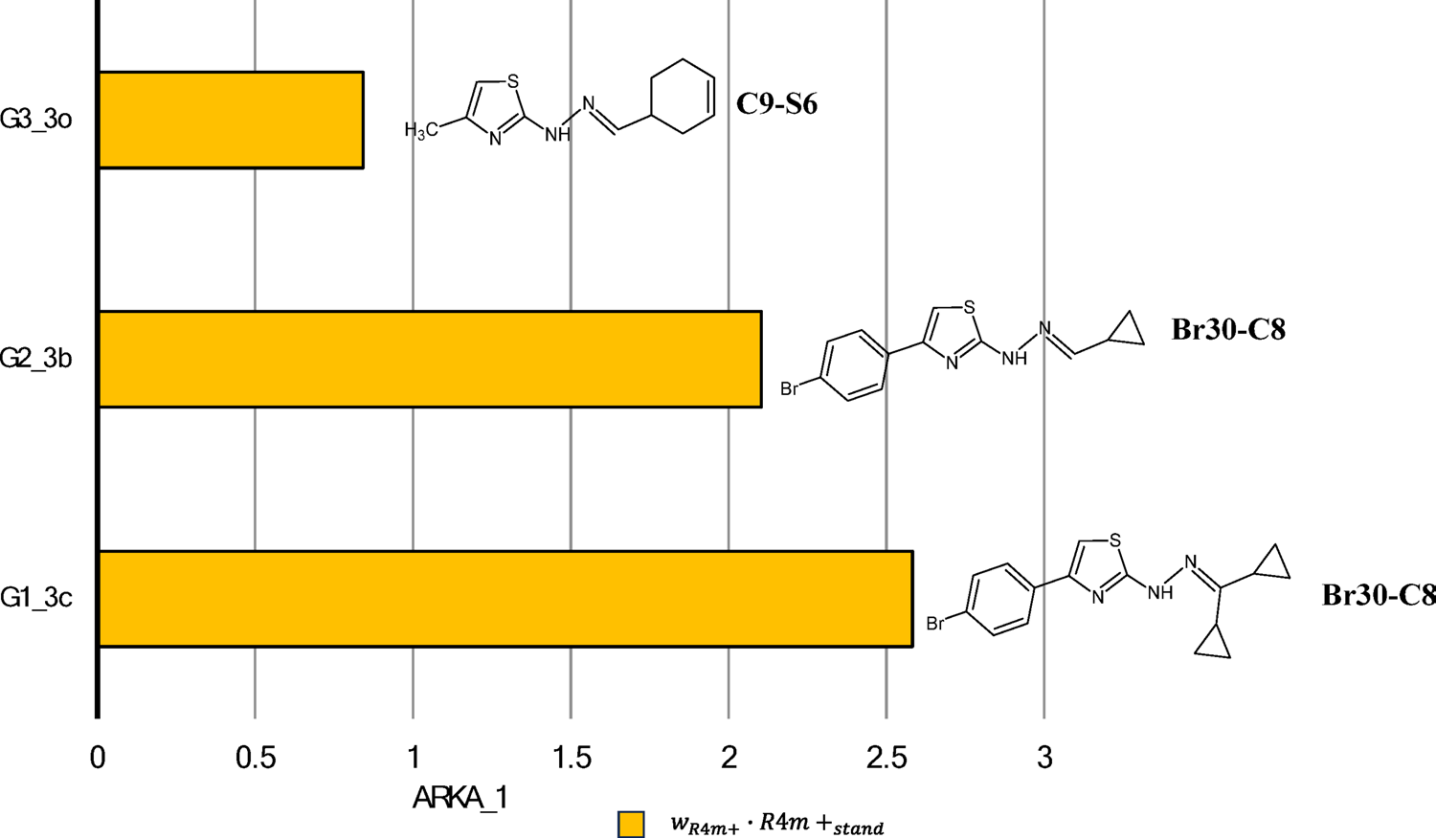
ARKA_1 values of positive compounds located in the “4—confident positives” region (green zone) of the ARKA 2 vs ARKA_1 plot (training set). The molecular structures of these compounds are provided, along with the specific atom pairs that determine the ${{\boldsymbol{R}}}_{4}^{+}\left({\boldsymbol{m}}\right)$ descriptor value

Since ARKA_1 exclusively captures the chemical information of the ${{\boldsymbol{R}}}_{4}^{+}\left({\boldsymbol{m}}\right)$ descriptor, the next part of our analysis focused on how ${{\boldsymbol{R}}}_{4}^{+}\left({\boldsymbol{m}}\right)$ characterizes the positive group of compounds located in “4—confident positives” region, marked by the green square in Fig. 3. To illustrate this, Fig. 5 presents the ARKA_1 values for three compounds from this region: G3_3o, G2_3b, and G1_3c.

The bar length equals the product of the weight and the standardized value of the ${{\boldsymbol{R}}}_{4}^{+}\left({\boldsymbol{m}}\right)$ descriptor. Since ARKA_1 only represents the ${{\boldsymbol{R}}}_{4}^{+}\left({\boldsymbol{m}}\right)$ descriptor in this case, its weight is 1, so the ARKA_1 value directly matches the standardized ${{\boldsymbol{R}}}_{4}^{+}\left({\boldsymbol{m}}\right)$ value. Based on paragraph “Interpretation of ${{\boldsymbol{R}}}_{4}^{+}\left({\boldsymbol{m}}\right)$ descriptor”, it can be concluded that the presence of the atomic pair Br–C plays a significant role in distinguishing compounds within the positive group. Compounds like G3_3i, G4_4i, and G4_4e are found in the boundary region where ARKA_2 values range between –0.5 and 0.5, indicating limited confidence in classification. Still, they show high ARKA_1 values driven by high ${{\boldsymbol{R}}}_{4}^{+}\left({\boldsymbol{m}}\right)$ descriptor scores. These scores are mainly affected by specific atomic pairs, especially F–F and Br–C. This implies that adding bromine or fluorine atoms greatly influences the biological activity of thiazole derivatives, even in structurally uncertain areas of the ARKA plot.

In the next phase of our analysis, we examined the role of the ARKA_2 descriptor in categorizing compounds G5_3j, G5_3i, and G4_4c as inactive, located in the “2—confident negatives” region of the ARKA plot (Fig. 3). We found that the ARKA_2 value is derived from a combination of several molecular descriptors, including RDF100e, RDF120s, ITH, and GATS8e, which together help classify these compounds as biologically inactive.

**Fig. 6:**
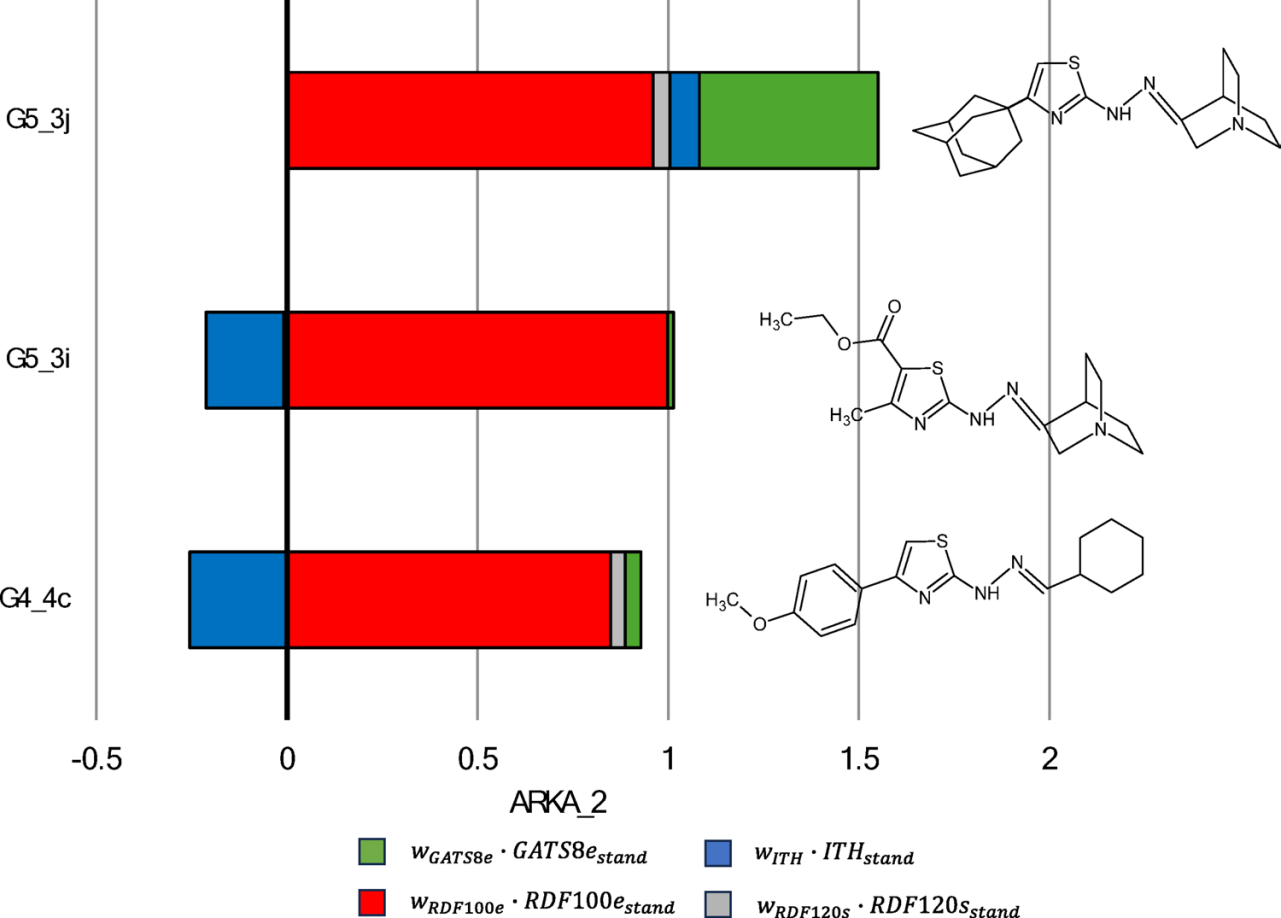
ARKA_2 values of negative compounds located in the “2—confident negatives” region (orange zone) of the ARKA 2 vs ARKA_1 plot (training set). The molecular structures of these compounds are provided

Cumulative bars display elements showing how each molecular descriptor contributes to the ARKA_2 value, which is calculated as the sum of the products of the standardized descriptor value and its respective weight. Interestingly, ARKA_2 identified exactly the three compounds—G5_3j, G5_3i, and G4_4c—that have the highest RDF100e descriptor values among all the molecules analyzed (Fig. S4). Additionally, ARKA_2—based on the RDF100e descriptor—highlighted compound G5_3i, which uniquely contains inter-fragment contribution ${{\boldsymbol{\delta}}}_{\mathbf{F}1-\mathbf{F}4}$ and ${{\boldsymbol{\delta}}}_{\mathbf{F}2-\mathbf{F}4}$ not seen in any other analyzed molecule (Fig. S4). Furthermore, ARKA_2 identified compound G5_3j as distinctive within the dataset, as its RDF100e value is almost entirely derived from a single type of inter-fragment contribution ${{\boldsymbol{\delta}}}_{\mathbf{F}2-\mathbf{F}3}$ (Fig. S4). This finding suggests that the RDF100e descriptor has a significant impact on the ARKA_2 scoring metric and may serve as a crucial structural descriptor for differentiating inactive compounds in the dataset. Based on Fig. 6, compound G5_3j shows the highest ARKA_2 value compared to G5_3i and G4_4c, mainly due to two factors: a positive contribution from the ITH descriptor and a strong positive influence from GATS8e. In other words, these two descriptors are key factors within the ARKA_2 profile that differentiate the compounds. The significant positive contribution of GATS8e for compound G5_3j was previously examined in Figure S17, in the section titled “Interpretation of GATS8e descriptor”. The negative spatial autocorrelation observed for G5_3j can be linked to 21 out of 65 atomic pairs showing notable differences in electronegativity values. Compound G5_3j demonstrates a positive contribution from the ITH descriptor to ARKA_2 compared to G5_3i and G4_4c, as it is the only one with a raw ITH value exceeding 67.069—the threshold at which the standardized descriptor value equals zero (Fig. S12). The ITH value of G5_3j is attributed to two key factors: (1) a large number of atoms in the molecule (excluding hydrogen atoms, denoted as A₀), which increases the overall complexity and connectivity of the structure; (2) a substantial number of atoms with influence indicators that satisfy the relationship ${{\boldsymbol{h}}}_{{\boldsymbol{i}}{\boldsymbol{i}}}$-${{\boldsymbol{h}}}_{{\boldsymbol{j}}{\boldsymbol{j}}}$≅0, reflecting a balanced distribution of atoms around the geometric center of the molecule.

## Conclusion

The study aimed to identify the structural components of thiazole derivatives contributing to their antifungal activity against *Candida albicans* (ATCC 2091). To achieve this, a quantitative structure–activity relationship (QSAR) model was developed, and molecular descriptors were interpreted following the model → descriptors → structure paradigm. Results indicated a negative correlation between all model descriptors and pMIC (Eq. 1).

**Scheme 3:**
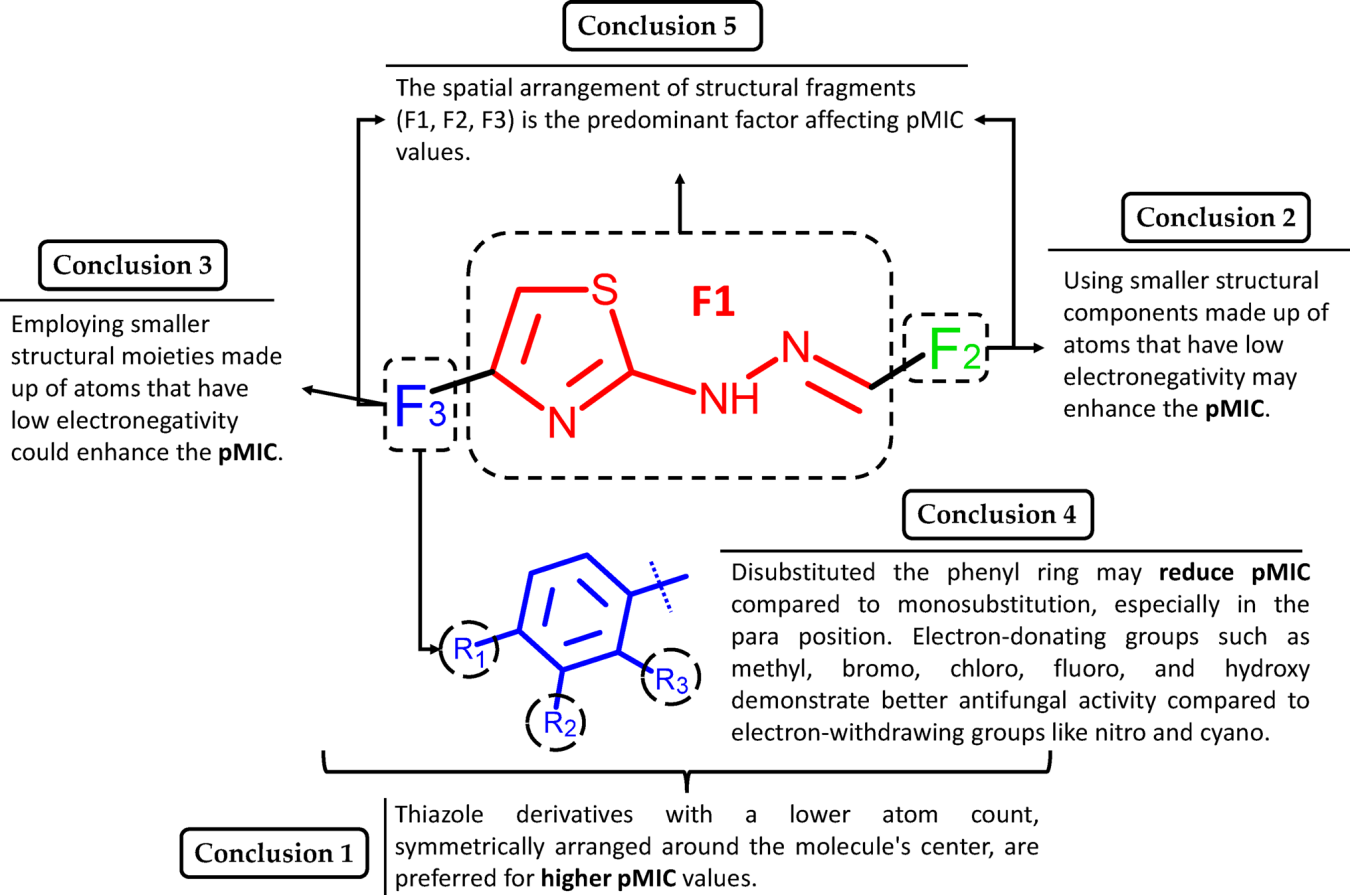
Interpretation of QSAR model: Summary of structure-antifungal activity relationship of thiazole derivatives against *Candida albicans* (ATCC 2091)

Scheme 3 provides a summary of the structural factors of thiazole derivatives influencing their antifungal efficacy against *Candida albicans* (ATCC 2091).

The justifications for the five conclusions indicated in Scheme 3 are summarized in detail in Table 4.

**Table 4:** Summary of conclusions from the interpretation of molecular descriptors of the QSAR model

Conclusion from Scheme 3	Molecular descriptor	Interpretation
1	I_TH_	When the number of atoms in a molecule (excluding hydrogen atoms) decrease, the ${A}_{0}\cdot {log}_{2}{A}_{0}$ value also decreases, resulting in a reduced I_TH_ value. If a structure has more symmetry elements, the value of $\sum_{g=1}^{G}{N}_{g}\cdot {log}_{2}{N}_{g}$ increases, leading to a lower ITH value
2, 3	RDF100e	The more atoms of the two fragments are separated by 10 Å, the higher the RDF100e value. The incorporation of atoms with low electronegativity values (${w}_{i}$) in the F2 and F3 fragments serves to decrease the RDF100e value. The F2 and F3 fragments of thiazole groups (G1–G5) are mainly composed of carbon and hydrogen atoms with low electronegativity values. Thus, as the size of these fragments decreases, the RDF100e value is observed to decline
	RDF120s	The more atoms of the F2 and F3 fragments are separated by 12 Å, the higher the RDF120s value. Therefore, to achieve low RDF120s values, it is preferred to have compact and small structural fragments F2 and F3. Moreover, the presence of atoms with low internal state values (${w}_{i}$) such as > CH–, –CH_2_–, > C=, and =CH– in the F2 and F3 fragments ensures a lower RDF120s value
	GATS8e	The GATS8e value exhibits a lower value when the square of the electronegativity difference between the *i*th atoms of the F2 fragment and the *j*th atoms of the remaining fragments is lower, assuming that both atoms are separated by a topological distance ${d}_{ij}$ equal to 8. F2 fragments consisting predominantly of low electronegativity atoms, such as carbon and hydrogen atoms, exhibit smaller sizes and, consequently lower GATS8e values
4	RDF100e	If the R1 substituent is part of the F3 fragment in a thiazole derivative and contains more atoms with higher electronegativity values, the molecule will exhibit an increased RDF100e value
	RDF120s	If R1 (in F3) contains more atoms with high internal state values, then the molecule will have a higher RDF120s value. The internal state value of an atom increases with its electronegativity and decreases with the number of σ bonds. The higher the internal state value of an atom, the more its valence electrons are available for intermolecular interactions
	${R}_{4}^{+}\left(m\right)$	${R}_{4}^{+}\left(m\right)$ increases with the presence of atomic pairs such as ${\gamma }_{F30-F29},$ ${\gamma }_{Cl37-C9},$ and ${\gamma }_{Br30-C8}$
	GATS8e	An increase in the GATS8e value occurs when the electronegativity values of the atoms in R1 substituents are significantly different from the electronegativity of another atom at a topological distance of 8 (${d}_{ij}$). The GATS8 values of the thiazole derivative are higher when the R1 substituent has more atoms with high electronegativity values
5	RDF100e, RDF120s	The mutual arrangement of fragments F3 and F2 (${\delta }_{\mathrm{F}3-\mathrm{F}2}$) in 3D space is crucial for defining the descriptor value. However, when the F3 fragment is made up of a substituted phenyl ring, the values of the ${\delta }_{\mathrm{F}3-\mathrm{F}2}$ contributions mainly rely on the structure of the F2 fragment. The RDF100e and RDF120s descriptors have higher values for larger F2 fragments In addition to the spatial arrangement of fragments F3 and F2, the spatial relations of fragments F3 and F2 in relation to the common core F1 are also important for the RDF100e value

Several studies have examined the antifungal activity of hydrazine thiazole derivatives, but researchers have developed only a limited number of interpretable QSAR models [19]. Lino and co‐workers constructed 2D, 3D-QSAR, and 3D similarity models to predict biological activities against another clinically significant species of *Candida* (*C. neoformans*) [19]. The scope of the research is constrained by the small sample size of compounds, which only comprises 22 molecules. In comparison to our compounds, the molecules discussed by Lino et al. share the same (4-(4-substituted-phenyl)-thiazol-2-yl)hydrazine scaffold but with different substitutions at the C2 positions of the thiazole nucleus. Our study examined a broader spectrum of compounds (n = 47) and substituents attached to the phenyl ring. Another difference is that Lino et al. employed HQSAR, CoMFA, CoMSIA, and ROCS to model antifungal activity in their research. Lino et al. summarized the findings from the models using a classic Structure–Activity Relationship (SAR) approach. Although our study focuses on a distinct species of *Candida* and different hydrazine thiazole derivatives, the conclusions drawn are broadly consistent with those reached by Lino et al. We have also demonstrated that the presence of bulky regions around the hydrazone linker and at the para position of the aryl group is unfavorable (the second and the third conclusions). In addition, the interpretation of I_TH_ molecular descriptor laid the foundation for understanding the intricate relationship between molecular size, symmetry, and their collective influence on the antifungal activity of 4-aryl-2-hydrazinothiazole derivatives. This finding supports previous SAR studies in the literature, which emphasize that linear aliphatic chains were found to be more active than cycloaliphatic rings or hindered aliphatic [20]. According to Carradori et al., the better activity could be attributed to the portion of the molecule on the hydrazone linker [20]. However, our research strongly suggests that the mutual spatial arrangement of moieties at N1-hydrazine and the 4-substituted-phenyl at the C4 position of the thiazole nucleus is crucial in determining antifungal activity. This could be the reason why thiazolyl-isatin derivatives exhibit lower antifungal activity compared to our compounds [33]. Several studies have shown that the hydrophobic features of hydrazone substituents play a significant role [18, 19, 21, 22]. Attempts to develop models incorporating lipophilicity descriptors did not meet the goodness of fit criteria. Notably, four of the five independent variables in our model are geometric descriptors (RDF100e, RDF120s, I_TH_, ${R}_{4}^{+}\left(m\right)$), suggesting that Van der Waals forces may play a more significant role than the electrostatic interactions in influencing the biological activity of the thiazole derivatives. Studies by Sunil D. et al. support this, based on MESP surface analysis, showing that thiazole and phenyl rings (electron-rich) interact via van der Waals forces with amino acid residues [34]. In our model, electronic effects were considered in three of the five descriptors using a weighting scheme based on electronegativity (RDF100e, GATS8e) and I-State (RDF120s). Our research suggests that an increase in the number of atoms with high electronegativity values in the substituents of the hydrazine linker and the phenyl ring reduces the antifungal activity of thiazole derivatives. This finding supports previous SAR studies in the literature, which have shown that electron-donating groups such as methyl, bromo, chloro, fluoro, and hydroxy demonstrate better antifungal activity compared to electron-withdrawing groups like nitro and cyano [18–24].

This paper presents the methodology for interpreting RDF(R), GETAWAY, and GATSk(w) class descriptors. The key to interpreting these descriptors lies in breaking down their values into atomic pair contributions (γ). Analyzing the significance and distribution of these atomic pair contributions helps understand the relationship between the structure, descriptor value, and biological activity. Using the proposed interpretation methodology, atomic pair contributions can serve as descriptors in predictive models. Furthermore, atomic pair contributions can also be divided into intra- (ε) and inter-fragment (δ) contributions, highlighting key structural fragments of the compounds with the greatest impact on the descriptor values. To improve interpretation, visualizing atomic pair contributions by overlaying their cumulative values (Ω) on molecular surfaces is beneficial, allowing us to intuitively determine the influence of individual atoms on the descriptor value using a color scale. Additionally, the ARKA method was used to identify activity cliffs by reducing the dataset's dimensionality to two main descriptors, ARKA_1 and ARKA_2. This reduction in dimensions allowed for clearer visualization of compound distribution and made it easier to detect regions where small structural changes caused significant shifts in antifungal activity. By mapping compounds onto a two-dimensional space defined by ARKA_1 and ARKA_2, the method uncovered subtle yet influential molecular features responsible for biological changes. Consequently, ARKA offered valuable insights into the structure–activity relationship, highlighting specific descriptors and atomic arrangements that contribute to antifungal activity.

Overall, the interpretation of QSAR model reveals several key molecular features influencing the antifungal activity of 4-aryl-2-hydrazinothiazole derivatives:

1. The spatial arrangement of moieties at N1-hydrazine and the 4-substituted-phenyl at the C4 position of the thiazole nucleus is crucial.
2. The Van der Waals force could be slightly more significant than the electrostatic force in influencing the antifungal activity.
3. The higher number of electronegative atoms in the substituents of the hydrazine linker and phenyl ring reduces the antifungal activity.
4. Electron-donating groups such as methyl, bromo, chloro, fluoro, and hydroxy enhance antifungal activity whereas electron-withdrawing groups like nitro and cyano decrease it.

These findings provide a basis for future modifications of 4-aryl-2-hydrazinothiazole derivatives for better antifungal activity. A summary of the structure–activity relationship observed from the molecular modeling studies is illustrated in Scheme 3.

### Design of newly proposed candidate compounds

**Fig. 7:**
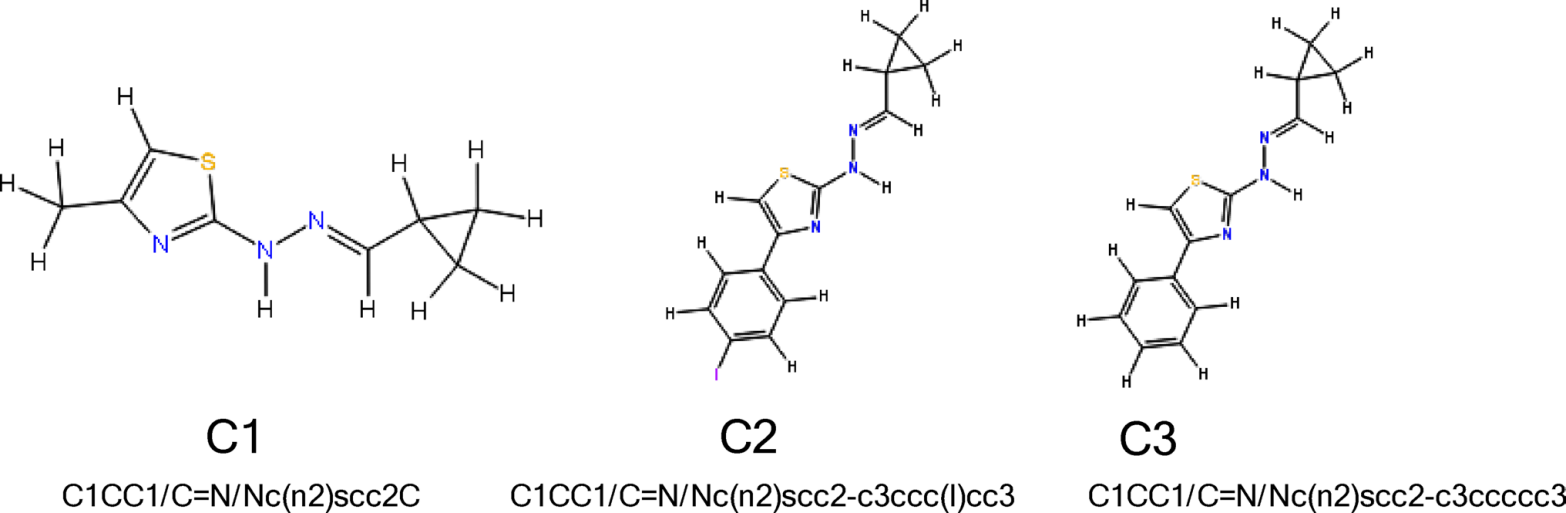
Molecular structures corresponding SMILES of the newly designed compounds

Based on interpretative conclusions presented in Scheme 3, Table 4, and insights derived from the **ARKA approach**, three novel compounds (**C1**, **C2**, and **C3**) were designed. The chemical structures and corresponding SMILES representations of these candidates are shown in Fig. 7.

#### Design rationale for C1

This compound was inspired by **G3_3o**, which exhibited the highest biological activity among the tested molecules. The ARKA_1 analysis indicated that the descriptor ${{\boldsymbol{R}}}_{4}^{+}\left({\boldsymbol{m}}\right)$ plays a key role in characterizing the group of compounds located in the “4**—**confident positives” region. To potentially enhance the pMIC value of the new molecule, we retained the atomic pair **C9–C6**, which was highlighted by ${{\boldsymbol{R}}}_{4}^{+}\left({\boldsymbol{m}}\right)$ as structurally significant. In contrast, the bulky substituent **cyclohex-3-enyl** was replaced with **cyclopropyl**, a group characteristic of the G2 group. This modification aligns with **Conclusions 1 and 2** (Scheme 3), leading to a noticeable reduction in the number of atoms and a more symmetrical spatial arrangement around the molecular center, which correlates with a lower **ITH** descriptor value. Furthermore, **Conclusion 5** supported the substitution with a smaller group, as it resulted in a significant decrease in **RDF100e** and **RDF120s** values.

#### Design rationale for C2

This compound was inspired by the series **G2_3a**, **G2_3b**, and **G2_3c**. The ARKA_1 analysis revealed that the addition of bromine or fluorine atoms strongly influences the biological activity of thiazole derivatives, even in structurally ambiguous regions of the ARKA plot. Based on **Conclusion 4** (Scheme 3), we inferred that substituting a halogen atom with the **lowest electronegativity** i.e. iodine could be beneficial. The core scaffold characteristic of the G2 group was preserved. This design choice is reflected in the reduced values of descriptors encoding electronegativity directly or indirectly: **RDF100e**, **RDF120s**, and **GATS8e**.

#### Design rationale for C3

In our model, four out of the five independent variables are **geometric descriptors** (**RDF100e**, **RDF120s**, **ITH**, **R_4⁺(m)**), suggesting that **Van der Waals forces** might influence the biological activity of thiazole derivatives more than electrostatic interactions. Therefore, we decided to investigate how activity would change if the molecule were **deprived of electronegative atoms**, while maintaining the atomic arrangement typical of the G2 compound group.

In Table 5, we present the calculated molecular descriptor values along with the predicted pMIC values for the three newly designed candidate compounds. The predicted pMIC values are relatively high, suggesting that the proposed compounds may represent promising candidates. The model interpretation appears to be both reasonable and informative. Notably, **C1** exhibits a predicted pMIC value of **8.41**, the highest among the designed structures.

**Table 5:** Standardized molecular descriptor values and predicted pMIC for three newly proposed compounds

	GATS8e	RDF100e	RDF120s	ITH	R4m+	pMIC
C1	− 2.1410	− 1.9145	− 1.8982	− 4.3533	− 0.4967	8.41
C2	− 0.2256	− 1.5133	0.0931	− 1.2592	2.1041	5.46
C3	0.0534	− 1.5133	0.3679	− 1.3592	− 0.2189	6.08

## Material and methods

For detailed information on the materials and methods, model validation, and the calculation algorithms of the selected molecular descriptors, please refer to the Supporting Information. This supplementary material provides comprehensive insights to ensure transparency and reproducibility of our research findings.

### Dataset

This study develops a QSAR model that balances prediction accuracy and mechanistic interpretability. It examines 50 thiazole derivatives with antifungal activity against *Candida albicans* (ATCC 2091), classified into five structural groups (G1–G5). Antifungal activity, obtained from the literature sources, was determined using MIC [μg/mL], representing the minimum inhibitory concentration preventing fungal growth [35–39]. MIC values were converted to mol/L for standardization and further transformed into pMIC to facilitate modeling. The structures and activity data (MIC, mol/L, pMIC) are summarized in Table S2, categorizing the compounds into six bioactivity levels: no bioactivity (MIC > 1000 μg/mL), mild (MIC = 501–1000 μg/mL), moderate (MIC = 126–500 μg/mL), good (MIC = 26–125 μg/mL), strong (MIC = 10–25 μg/mL), very strong (MIC < 10 μg/mL).

### Computational chemistry

The geometry optimization of the studied compounds was influenced by their initial structure, derived from Molecule-1, which shares a common core with the investigated thiazoles. The molecular geometry of Molecule-1 was previously optimized using DFT (B3LYP/6-311G), and its Cartesian coordinates were sourced from Łączkowski et al. [40].

The structure preparation followed four stages (Scheme S1):

1. Extraction of common cores from Molecule-1
2. Attachment of molecular fragments (M1–M5), forming five maximum common substructures (MCS1–MCS5).
3. Optimization via molecular mechanics (MM) and refinement using PM6 and DFT (B3LYP/6-31G**) methods**.
4. Addition of substituents (R1–R3) to generate thiazole derivatives (G1–G5), followed by full geometry optimization using B3LYP hybrid functionals (B3LYP/6-311g**).

The vibrational frequencies of optimized structures were evaluated at the DFT/6-311G level using Gaussian 09 (PL-Grid Infrastructure) [41, 42]. The result files from geometry optimization have been deposited in the NOMAD repository, allowing reviewers to access the data [43]. Corrections to molecular representations were applied via alvaMolecule, and descriptor analysis was performed using alvaDesc, yielding 5471 molecular descriptors [44]. Additionally, DFT-based global reactivity indices related to electronic structure properties were computed. In this study, we have used the novel supervised dimensionality reduction technique—the ARKA framework to assess the modelability and check the presence/absence of potential activity cliffs. Using the selected feature matrix of the QSAR model, we have computed the ARKA descriptors using the tool ARKAdesc-v2.0 [30]. To support reproducibility and facilitate further analysis, a supplementary SDF file containing the molecular structures and corresponding SMILES notations has been provided.

### Model development and validation

The collected data underwent variable reduction, selection, and pre-treatment to eliminate irrelevant descriptors, improving model accuracy and interpretability. Molecular descriptors were standardized, ensuring comparability across different scales.

The dataset was systematically divided into training and test subsets using the Kennard–Stone algorithm, which ensures uniform coverage of the chemical space and avoids random bias. Specifically, 70% of the compounds (n = 33) were assigned to the training set for descriptor selection, variable screening, and model construction. The remaining 30% (n = 14) formed the independent test set, used exclusively for external validation of the QSAR model’s predictive performance. The QSAR model was developed using a hybrid Genetic Algorithm (GA) and Multiple Linear Regression (MLR) approach, optimizing descriptor selection. GA settings included 500 iterations, mutation probability of 0.3, and crossover probability of 1, ensuring robust feature selection. The final GA-MLR model achieved a fitness score of 0.6147.

Validation included internal cross-validation, Y-randomization, external validation, and applicability domain analysis (Sect. 3, Supplementary Information). The model demonstrated high predictive reliability, explaining 85.14% of pMIC variation (R^2^), with minimal collinearity (r < 0.3611). ANOVA confirmed strong statistical significance (F = 30.9352, *p* < 2.3E-10). Error analysis using PRESS, MSE, RMSE, and MAE further validated model robustness. Ultimately, the antifungal activity of thiazole derivatives was found to be significantly influenced by RDF100e, ITH, ${R}_{4}^{+}\left(m\right)$, RDF120s, and GATS8e, highlighting key molecular features affecting bioactivity. The goodness-of-fit parameters (R^2^, RMSE) can be misleading in MLR models, often overestimating accuracy in smaller datasets. To mitigate this, we conducted internal (LOO-CV) and external validation beyond standard model assessments. Validation confirmed high robustness (Q^2^(LOO) = 0.7809) and model stability (|R^2^—Q^2^(LOO)|= 0.0705), indicating no overfitting. Further validation with ${\overline{r}}_{m}^{2}$ metrics and concordance correlation coefficient (CCC = 0.8866) ensured predictive reliability. Additionally, Golbraikh-Tropsha criteria verified experimental vs. predicted alignment. Y-randomization tests ruled out accidental correlations in descriptor selection. Applicability domain analysis using William’s plot identified outliers, leading to final model refinement. Overall, metrics confirm the QSAR model is robust, statistically significant, and highly predictive for antifungal activity of thiazole derivatives against *Candida albicans* (ATCC 2091).

### The calculation algorithms of selected molecular descriptors

Molecular descriptors play a key role in QSAR models, offering insights into molecular properties and behavior. Their selection depends on molecular representation and calculation algorithms. This study outlines five key descriptors: RDF100e, I_TH_, ${R}_{4}^{+}\left(m\right)$, RDF120s, and GATS8e, detailing their methodology in Sects. 4.1–4.4 (Supplementary Information) [45].

The Radial Distribution Function (RDF) class analyzes internal molecular structure, estimating atom pair probabilities within a spherical shell. RDF100e incorporates electronegativity, while RDF120s utilizes I-State weighting. Atomic pair contributions (${\gamma }_{ij}$) are influenced by interatomic distances and physicochemical properties, defining descriptor values. Geometric structure is represented using Cartesian coordinates, with Euclidean distances (${r}_{ij}$) computed. The R-value encodes atom distance probabilities, scaled tenfold (e.g., 10 Å for RDF100e, 12 Å for RDF120s). Descriptors are evaluated based on ${\gamma }_{ij}$ distribution, ranking their contributions to bioactivity.

The Total and Standardized Information Content on Leverage Equality (I_TH_**)** belongs to the GETAWAY descriptor class, characterizing molecular structure using atomic leverage values rather than physicochemical properties. I_TH_ assesses the distribution of atomic positions within a molecule by measuring how atoms are spatially arranged based on their leverage values. The leverage quantifies an atom's distance from the molecular center, with peripheral atoms having the highest leverage values. Molecular symmetry and atomic arrangement influence I_TH_ values. A molecule with uniform atomic leverage exhibits a low I_TH_ value, indicating a highly symmetrical structure. In contrast, molecules with diverse atomic leverage values display higher entropy, reflecting a more complex spatial arrangement. Unlike RDF descriptors, which focus on atomic pairs, I_TH_ evaluates atomic sets, offering a distinctive geometric perspective on molecular structure.

The ${R}_{4}^{+}\left(m\right)$ descriptor, part of the GETAWAY class, integrates physicochemical, topological (2D), and geometric (3D) properties to characterize molecular structure [67]. Unlike other descriptors, ${R}_{4}^{+}\left(m\right)$ identifies a single atomic pair with the highest contribution, making it highly selective in assessing molecular interactions. This ${R}_{4}^{+}\left(m\right)$ evaluates atomic mass-weighted leverage, measuring distances relative to the molecular center while also incorporating topological connections between atoms. By prioritizing the most significant atomic interaction, ${R}_{4}^{+}\left(m\right)$ provides a focused representation of molecular properties, aiding in precise bioactivity predictions within QSAR studies.

The GATS8e descriptor, the only 2D molecular descriptor in the QSAR model, combines physicochemical and topological properties to characterize molecular structure. It applies Sanderson electronegativity as a weighting method, assessing spatial autocorrelation—the clustering of atoms with similar properties.

This descriptor evaluates atomic property distributions, identifying patterns in molecular topology. A value close to zero indicates strong positive autocorrelation, while values above one suggests random spatial distribution. GATS8e plays a crucial role in analyzing molecular size, shape, and topology, aiding in the assessment of structural features influencing antifungal activity.

Computational algorithms for selected molecular descriptors provide combined information regarding physicochemical properties of atoms, molecular topology, size and shape of compounds. Therefore, the impact of different structural features on the anti-candidal activity of thiazole derivatives can be assessed by a thorough understanding of the methodology for the calculation of individual descriptors. Table 6 is a summary of the most important features of the analyzed descriptors.

**Table 6:** Summary of calculation algorithms used for selected molecular descriptors

	RDF(R)	GETAWAY		${\mathrm{GATS}}_{\mathrm{k}}\left(\mathrm{w}\right)$
	RDF100e RDF120s	${R}_{k}^{+}\left(w\right):$ ${R}_{4}^{+}\left(m\right)$	I_TH_	GATS8e
Number of dimensions in a Euclidean space	3	3	3	2
Additive nature of molecular descriptor. Interpretation based on atomic pair contributions	+^a^	+	–	–
the sign of atomic pair contribution	Positive	Positive	–	–
Physicochemical properties of atoms	RDF100e: electronegativity RDF120s: I-state	Atomic mass	–	Electronegativity
Topological description of molecular structure				
D^2D^: topological distance matrix	–	+ (H-filled molecular graph)	–	+ (H- depleted molecular graph)
$\delta \left(k;{d}_{ij}\right):$ Dirac delta function	–	k = d_ij_ = 4	–	k = d_ij_ = 8
Geometric description of molecular structure				
M: Cartesian coordinates	Non-centered	Centered	Centered	–
D^3D^: distance matrix	+	+	+	
H: molecular influence matrix	–	+ (H-filled molecular graph)	+ (hydrogen depleted molecular graph)	

^a^The plus sign indicates that a feature is included in the descriptor calculation methodology. The minus sign means that the feature does not influence the descriptor value

## Supplementary Information

Below is the link to the electronic supplementary material.

Supplementary Material 1

## Data Availability

No datasets were generated or analysed during the current study.

## Appendix

Please refer to the Supporting Information to access the frequently used symbols, detailed information on materials and methods, model validation, and the calculation algorithms of selected molecular descriptors. An SDF file containing molecular structures of the studied compounds and their SMILES notations is provided.

The authors have cited additional references within the Supporting Information [45–66].
