# Supplementary material for: Interpretable Quantitative Structure–Activity Relationship (QSAR) for identification of potent antifungal activity agents towards Candida albicans ATCC 2091
Source: Mol Divers. 2025 Nov 28;30(3):4635–59. doi: 10.1007/s11030-025-11404-2 (PMC13198517; doi:10.1007/s11030-025-11404-2)
Supplement: Supplementary file 1 — Supplementary Material 1 [file 11030_2025_11404_MOESM1_ESM.docx]

Supporting Information

Interpretable Quantitative Structure-Activity Relationship (QSAR) for Identification of Potent Antifungal Activity Agents Towards *Candida albicans* ATCC 2091

Mariusz Zapadka,*^[a]^ Krzysztof Zbigniew Łączkowski,^[b]^ Anna Budzyńska,^[c]^ Mateusz Maciejewski,^[d]^ Przemysław Dekowski,^[e]^ and Bogumiła Kupcewicz*^[a]^

[a] Dr. M. Zapadka, Prof. B. Kupcewicz
Department of Inorganic and Analytical Chemistry
Nicolaus Copernicus University in Toruń, Ludwik Rydygier Collegium Medicum in Bydgoszcz
Jurasza 2, 85-089 Bydgoszcz, Poland
Dr. M. Zapadka: https://orcid.org/0000-0002-7968-4665
Prof. B. Kupcewicz: https://orcid.org/0000-0002-4480-7338
E-mail: kupcewicz@cm.umk.pl, mariusz.zapadka@cm.umk.pl

[b] Prof. K.Z. Łączkowski
Department of Chemical Technology and Pharmaceuticals
Nicolaus Copernicus University in Toruń, Ludwik Rydygier Collegium Medicum in Bydgoszcz
Jurasza 2, 85-089 Bydgoszcz, Poland
https://orcid.org/0000-0003-2107-2719

[c] Dr. A. Budzyńska
Department of Microbiology
Nicolaus Copernicus University in Toruń, Ludwik Rydygier Collegium Medicum in Bydgoszcz
Jurasza 2, 85-089 Bydgoszcz, Poland
https://orcid.org/0000-0002-8545-177X

[d] Dr. M. Maciejewski
Faculty of Mathematics and Computer Science
Nicolaus Copernicus University in Toruń
Chopina 12/18, 87-100 Toruń
https://orcid.org/0000-0002-7294-9448

[e] MSc P. Dekowski
New Technologies Department
Softmaks.pl Sp. z o.o.
Kraszewskiego 1, Bydgoszcz, Poland

**Table of content**

[1. Frequently Used Symbols 3](#_Toc212844358)

[2. Material and Methods 4](#_Toc212844359)

[2.1. Dataset 4](#_Toc212844360)

[2.2. Geometry Optimization Methods 7](#_Toc212844361)

[2.3. Calculation of Molecular Descriptors. 7](#_Toc212844362)

[2.4. Variable’s Reduction, Selection, and Pre-treatment 8](#_Toc212844363)

[2.5. Data Split 8](#_Toc212844364)

[2.6. Model Development 8](#_Toc212844365)

[2.7. Multi-criteria Decision-making (MCDM) 8](#_Toc212844366)

[3. Model Validation 9](#_Toc212844367)

[4. The Calculation Algorithms of Selected Molecular Descriptors 14](#_Toc212844368)

[4.1. Radial distribution function - RDF100e and RDF120s 14](#_Toc212844369)

[4.2. The total and standardized information content on the leverage equality - I_TH_ 15](#_Toc212844370)

[4.3. R maximal autocorrelation of lag 4 / weighted by mass -$R_{4}^{+}\left( m \right)$ 15](#_Toc212844371)

[4.4. Geary autocorrelation of lag 8 weighted by Sanderson electronegativity (GATS8e) 17](#_Toc212844372)

[5. Supporting Tables 18](#_Toc212844373)

[6. Supporting Figures 20](#_Toc212844374)

[7. Supporting Scheme 33](#_Toc212844375)

[8. References 33](#_Toc212844376)

1. Frequently Used Symbols

The following list contains the most frequently used symbols in this article (Table S1). To avoid redundancy, subscripts are only noted in exceptional cases.

**Table S1**. List of the most used symbols in this article.

| Symbol | Designation |
| --- | --- |
| A | number of the atoms (hydrogens included) |
| $A_{0}$ | number of non-hydrogen atoms |
| $\beta$ | regression coefficient of QSAR model |
| $d_{ij}$ | topological distance between *i*th and *j*th atoms |
| $\delta\left( k;d_{ij} \right)$ | Dirac delta function |
| $\delta_{t}$ | total inter-fragment contribution |
| $\delta_{Fi-Fj}$ | The inter-fragment contribution delineates the spatial relationship between the *i*th and *j*th fragments. |
| e | electronegativity as a weighting scheme |
| $\varepsilon_{t}$ | total intra-fragment contribution |
| Fi | the subset of atoms of the molecule ($\mathcal{M}$) constituting the *i*th molecular fragment |
| $\varepsilon_{\mathrm{Fi}}$ | intra-fragment contribution of *i*th molecular fragment (Fi) |
| F1 | the largest common substructure of the molecules in a series (common core) |
| F2 | second molecular fragment |
| F3 | third molecular fragment |
| F4 | fourth molecular fragment |
| G | the number of equivalence classes |
| $\gamma_{ij}$ | The contribution of a pair of atoms (i and j) to the value of the molecular descriptor. |
| $h$ | leverage value |
| i, j | *i*th and *j*th atoms constitute elements of the molecular structure ($\mathcal{M}$) of compound |
| m | atomic mass as a weighting scheme |
| M | molecular matrix |
| $\mathcal{M}$ | the arrangement of the specifically numbered atoms that comprise a molecule |
| $N_{g}$ | the number of atoms with the same leverage value |
| $\Omega_{n}$ | Cumulative contribution of *n*th atom of molecular structure |
| $r_{ij}$ | interatomic distances between the *i*th and *j*th atoms |
| R | spherical volume of radius R |
| s | I-State as a weighting scheme |
| $w_{i},w_{j}$ | the atomic property of the *i*th and *j*th atoms |
| x,y,z | geometric coordinates |

1. Material and Methods
   1. Dataset

This study attempts to develop the QSAR model that balances prediction accuracy and mechanistic interpretability. For this purpose, 50 compounds of thiazole derivatives and their antifungal activities against Candida albicans (ATCC 2091) were used (Table S2). Based on structure, the compounds were divided into five classes of thiazoles. (G1-G5). The antifungal activity of the compounds under study was determined using MIC [μg/mL] (i.e., the lowest chemical concentration that prevents the visible growth of a fungal strain). Subsequently, the MIC values obtained in µg/mL were converted to moles per liter (mol/L) for a more standardized comparison. The inverse decimal logarithm of the MIC values, now expressed in mol/l, was calculated to facilitate the modeling process. The structures of the compounds, along with their activities expressed in MIC [μg/mL and mol/L] and pMIC [mol/L], are presented in Table S2. The compounds were divided into five activity groups: no bioactivity (MIC >1000 μg/mL), mild (MIC = 501–1000 μg/mL), moderate (MIC = 126–500 μg/mL), good (MIC = 26–125 μg/mL), strong (MIC = 10–25 μg/mL) and very strong bioactivity (MIC < 10 μg/mL).

**Table S2**. Compounds used in the QSAR study.

| ***Group 1*** | |  | | | | | | | |
| --- | --- | --- | --- | --- | --- | --- | --- | --- | --- |
| **Nr** | **Name** | **R_1_** | **MIC [µg/mL]** | **MIC [µM]** | | | | | **pMIC [M]** |
| 1 | G1_3a | F | 1.95 | 6.47 | | | | | 5.19 |
| 2 | G1_3b | Cl | 0.24 | 0.76 | | | | | 6.12 |
| 3 | G1_3c | Br | 0.98 | 2.70 | | | | | 5.57 |
| 4 | G1_3d | CN | 7.81 | 25.32 | | | | | 4.60 |
| 5 | G1_3e | CH_3_ | 0.24 | 0.81 | | | | | 6.09 |
| 6 | G1_3f | N_3_ | 3.91 | 12.05 | | | | | 4.92 |
| 7 | G1_3g | CF_3_ | 15.62 | 44.45 | | | | | 4.35 |
| 8 | G1_3h | NO_2_ | 7.81 | 23.78 | | | | | 4.62 |
| 9 | G1_3i | NHCO(CH_2_)_2_Cl | 250 | 642.74 | | | | | 3.19 |
| 10 | G1_3j | NHCOCH_2_Cl | 500 | 1333.58 | | | | | 2.87 |
| 11 | G1_3k | 3,4-diCl | 3.91 | 11.10 | | | | | 4.95 |
| ***Group 2*** | |  | | | | | | | |
| **Nr** | **Name** | **R_1_** | **MIC [µg/mL]** | **MIC [µM]** | | | | | **pMIC [M]** |
| 12 | G2_3a | F | 1.95 | 7.46 | | | | | 5.13 |
| 13 | G2_3b | Br | 0.12 | 0.37 | | | | | 6.43 |
| 14 | G2_3c | Cl | 0.12 | 0.43 | | | | | 6.36 |
| 15 | G2_3d | CH_3_ | 0.12 | 0.47 | | | | | 6.33 |
| 16 | G2_3f | O-CH_3_ | 0.24 | 0.88 | | | | | 6.06 |
| 17 | G2_3g | 2,4-diF | 1.95 | 6.98 | | | | | 5.16 |
| 18 | G2_3h | CN | 1.95 | 7.27 | | | | | 5.14 |
| 19 | G2_3i | N_3_ | 0.98 | 3.45 | | | | | 5.46 |
| 20 | G2_3j | NO_2_ | 3.91 | 13.56 | | | | | 4.87 |
| ***Group 3*** | |  | | | | | | | |
| **Nr** | **Name** | **R_1_** | **R_2_** | | **MIC [µg/mL]** | **MIC [µM]** | | | **pMIC [M]** |
| 21 | G3_3a | F | - | | 0.48 | 1.59 | | | 5.80 |
| 22 | G3_3b | Br | - | | 7.81 | 21.56 | | | 4.67 |
| 23 | G3_3c | Cl | - | | 0.48 | 1.51 | | | 5.82 |
| 24 | G3_3d | CH_3_ | - | | 0.48 | 1.61 | | | 5.79 |
| 25 | G3_3f | O-CH_3_ | - | | 1.95 | 6.22 | | | 5.21 |
| 26 | G3_3g | NO_2_ | - | | 1000 | 3044.79 | | | 2.52 |
| 27 | G3_3h | CN | - | | 7.81 | 25.32 | | | 4.60 |
| 28 | G3_3i | 2,4-diF | - | | 7.81 | 24.45 | | | 4.61 |
| 29 | G3_3j | N_3_ | - | | 3.91 | 12.05 | | | 4.92 |
| ***Group 3***  cont. | |  | | | | | | | |
| **Nr** | **Name** | **R_1_** | **R_2_** | | **MIC [µg/mL]** | **MIC [µmol/L]** | | | **pMIC [mol/L]** |
| 30 | G3_3l | - | CH_2_Cl | | 125 | 488.66 | | | 3.31 |
| 31 | G3_3n | - | CH_2_COOC_2_H_5_ | | 0.015 | 0.05 | | | 7.29 |
| 32 | G3_3o | - | CH_3_ | | 0.03 | 0.14 | | | 6.87 |
| ***Group 4*** | |  | | | | | | | |
| **Nr** | **Name** | **R_1_** | **R_2_** | | **MIC [µg/mL]** | **MIC [µM]** | | | **pMIC [M]** |
| 33 | G4_4b | F | - | | 7.81 | 25.74 | | | 4.59 |
| 34 | G4_4c | O-CH_3_ | - | | 15.62 | 49.51 | | | 4.31 |
| 35 | G4_4d | CN | - | | 125 | 402.63 | | | 3.40 |
| 36 | G4_4e | Br | - | | 125 | 343.09 | | | 3.46 |
| 37 | G4_4f | Cl | - | | 62.5 | 195.38 | | | 3.71 |
| 38 | G4_4h | CH_3_ | - | | 7.81 | 26.08 | | | 4.56 |
| 39 | G4_4i | 2,4-diF | - | | 62.5 | 194.44 | | | 3.71 |
| ***Group 4***  cont. | |  | | | | | | | |
| **Nr** | **Name** | **R_1_** | **R_2_** | | **MIC [µg/mL]** | **MIC [µM]** | | | **pMIC [M]** |
| 40 | G4_4j | - | COOC_2_H_5_ | | 3.91 | 12.63 | | | 4.90 |
| ***Group 5*** | |  | | | | | | | |
| **Nr** | **Name** | **R_1_** | **R_2_** | | **R_3_** | | **MIC [µg/mL]** | **MIC [µM]** | **pMIC [M]** |
| 41 | G5_3a | F | - | | - | | 15.62 | 49.36 | 4.31 |
| 42 | G5_3b | Cl | - | | - | | 7.81 | 23.46 | 4.63 |
| 43 | G5_3c | CH_3_ | - | | - | | 15.62 | 49.99 | 4.30 |
| 44 | G5_3d | Br | - | | - | | 15.62 | 41.40 | 4.38 |
| 45 | G5_3f | CF_3_ | - | | - | | 31.25 | 85.28 | 4.07 |
| 46 | G5_3g | NHCOCH_3_ | - | | - | | 500 | 1406.43 | 2.85 |
| 47 | G5_3h | NHSO_2_CH_3_ | - | | - | | 125 | 319.23 | 3.50 |
| ***Group G5***  cont. | |  | | | | | | | |
| **Nr** | **Name** | **R_1_** | **R_2_** | | **R_3_** | | **MIC [µg/mL]** | **MIC [µM]** | **pMIC [M]** |
| 48 | G5_3i | - | CH_3_ | | COOC_2_H_5_ | | 62.5 | 202.63 | 3.69 |
| 49 | G5_3j | - |  | | H | | 250 | 701.09 | 3.15 |
| 50 | G5_3k | - |  | | H | | 500 | 1364.33 | 2.87 |

- 1. Geometry Optimization Methods

The outcome of the geometry optimization procedure is significantly affected by the initial structure of the compound. Therefore, the studied compounds were acquired by altering the initial structure (Molecule-1), which has a common core with the thiazoles being studied. The molecular geometry of Molecule-1 had already been optimized using density functional theory (DFT) approximations (B3LYP/6-311G**). Therefore, the optimized Cartesian coordinates of the starting structure were taken from the Supplementary Material of the Łączkowski et al. research [40]. The molecular structure preparation routine of thiazole derivatives is illustrated in Scheme S1.

**Scheme S1**. Methodology for preparing molecular structures of the studied compounds.

In the first stage, two common cores were separated from Molecule-1. In the second step, the molecular fragments (M1-M5) were attached to respective common core, forming five maximum common substructures (MCS1-MCS5). In the third stage, the MCSs were investigated based on molecular mechanics (MM) calculations, and their further optimization was performed at semiempirical PM6 and Density Functional Theory (B3LYP/6-31G**) levels [38]. Next, substituents (R1-R3) were added to the MCSs to create a range of thiazole derivatives (G1-G5). In the fourth final stage, the geometries of thiazole derivatives were fully optimized using B3LYP hybrid density functional combined with 6-31G** and 6-311g** [46]. The corresponding vibrational frequencies were evaluated at the same level of theory (DFT/6-311g**). All calculations were carried out using the Gaussian 09 package in PL-Grid Infrastructure [41,42].

- 1. Calculation of Molecular Descriptors.

Geometry-optimized structures of the investigated compounds were analyzed with alvaMolecule v1.0.4, which was used to fix erroneous representations of molecules. Next, alvaDesc v2.0.10 was used to calculate and analyze 5471 molecular descriptors (from 0- to 3-dimensional descriptors). This software is one of the most recent tools used for this purpose [44]. Additionally, DFT-based global reactivity indices were calculated that are associated with electronic structure principles (chemical potential, hardness, softness, electron affinity, ionization potential, electronegativity, electrophilicity, electrodonating power, electroacepting power, net electrophilicity).

- 1. Variable’s Reduction, Selection, and Pre-treatment

After data collection, it is necessary to perform variable reduction, selection, and pre-treatment (also known as pre-processing). The purpose of variable reduction and selection is to eliminate irrelevant or insignificant descriptors in predicting a compound's biological activity. This improves the speed, performance, and interpretability of models. In the pre-treatment, the molecular descriptors were standardized. This makes it easier to compare variables, even if they were measured differently. Standardized variables have a mean of zero and a standard deviation of one. According to Tetko et al, the QSAR model was developed after performing descriptor pre-selection [47]. Data pre-treatment was carried out in the V-WSP tool implemented in DTC-QSAR: A complete QSAR modeling package (available at http://teqip.jdvu.ac.in/QSAR_Tools/) [48-50]. The applied protocol included: (1) the descriptors that are constant among all chemicals were removed; (2) the normalized descriptors with a variance less than 0.001 were excluded; and (3) the descriptors that were cross-correlated with linear correlation coefficient R2 higher than 0.9900 were grouped, and only the first descriptor from the group was selected for the development of the model. Following Racz et al. recommendations, the intercorrelation limit was checked before finalizing the selection, as the specific choice is inherently dataset-dependent [51].

- 1. Data Split

The data set was divided into a training set and an independent test subset using Kennard-Stone. The training set, consisting of approximately 70% of the compounds (n=33), was used for feature selection, variable screening, and model construction. The remaining compounds (n=14) included in the test set were used to evaluate the predictive performance of the models (external validation of the models).

- 1. Model Development

The QSAR model was developed using a hybrid method that combined genetic algorithms (GA) and multiple linear regression (MLR), which are available in the DTC-QSAR software. GA is a stochastic technique that mimics natural selection and evolution. The process begins by generating a population of random solutions representing chromosomes in a biological system. These solutions consist of binary vectors that indicate whether each descriptor is present in the model. GA operators were set to the number of iterations- 500, equation length- 5, mutation probability- 0.3, crossover probability- 1, the initial number of equations generated- 500, and the number of equations selected in each generation- 200. The low probability of mutation preserves the most significant descriptors. Each generated/optimized QSAR equation is evaluated using the mean of absolute errors (MAE) criteria [52-54]. The final GA-MLR model reached a fitness score of 0.6147.

- 1. Multi-criteria Decision-making (MCDM)

In the context of QSAR studies, multi-criteria decision-making (MCDM_) is used to assess various models by applying multiple validation parameters. MCDM allows for a comprehensive evaluation of models, considering various aspects of their performance and helps them make informed and balanced decisions. Each statistical metric offers only a single projection of the model error as it condenses vast amounts of data into one value. Therefore, a combination of metrics was used to assess properly model performance [55]. The model quality was evaluated using goodness-of-fit, leave-one-out cross-validation, Y-randomization, MAE, and Golbraikh Tropsha metrics. Internal validation metrics include determination coefficient (R^2^), adjusted determination coefficient (R^2^_a_), standard error of estimate (SEE), predicted residual error sum of squares (PRESS), and Fisher F-statistics. Subsequently, the model quality was also judged by leave-one-out metrics: Q^2^, $r_{m}^{2}$, $\overline{r}_{m}^{2}$, ${\Delta r}_{m}^{2}$. The model robustness was verified with a Y-randomization test with a 200-response permutation ($\overline{R}$, $\overline{R}^{2}$,$\overline{Q}^{2}$,${}^{C}{R_{p}^{2}}$). The external validation involved predicting the antifungal activity of the test set. The results were expressed using various metrics: R^2^, $R_{0}^{2}$, reverse $R_{0}^{2}$, root mean square error of prediction (RMSEP), $Q_{F1}^{2}$, $Q_{F2}^{2}$, $\overline{r}_{m}^{2}$ (test). The model’s predictive performance was evaluated based on the MAE value employing both 100% and 95% data points. The reliability of the model was investigated using criteria based on the regression through the origin (RTO) proposed by Golbraikh and Tropsha [56,57]. The MLR plus Validation GUI v1.3 software was used for these analyses. Besides model validation, the assessment of the applicability domain (AD) is a fundamental aspect of QSAR analysis. The AD is a theoretical region in chemical space, described by molecular descriptors and biological activity, where the model makes reliable predictions. A model developed without a defined AD could predict the activity of all types of compounds, leading to unreliable predictions. The AD is investigated using William’s plot, which shows the distribution of standardized residuals against the leverage. The AD is an area within ±3 of standardized residuals and a leverage cutoff h=3*p/n, where p is the number of model parameters and n is the number of compounds in the training set.

1. Model Validation

QSAR aims to develop a robust, unbiased model capable of accurately predicting new compound's properties with reliability and precision. To determine the most significant QSAR model, several validation techniques were applied: (a) internal validation and cross-validation using the training set compounds, (b) Y-randomization, (c) external validation using the test set compounds, (d) Golbraikh and Tropsha acceptable model criteria, and (e) applicability domain analysis. Table S3 presents a detailed evaluation of the validation metrics for the most effective model.

**Table S3**. Validation metrics of best-fitted model.

| **Validation metrics** | **Criteria** | **Score** |
| --- | --- | --- |
| **goodness-of-fit and internal validation** (training set) **(n=33)** | | |
| number of molecular descriptor (p) | $5\cdot p\leq n$ (n – number of compounds) | $5\cdot5\leq34$ |
| Molecular descriptor correlations | $\leq$ 0.8 | Table 5 |
| r | $\geq$ 0.8 | 0.9227 |
| R^2^ | $\geq$0.7 | 0.8514 |
| R^2^_adj_ | R^2^$\approx$R^2^_adj_ | 0.8239 |
| df_regression_ (p) | $5\cdot p\leq n$ | 5 |
| SSR (regression) | the bigger the better | 25.0574 |
| MSR (regression) | the bigger the better | 5.0115 |
| df_residual_ (n-p-1) | the bigger the better | 27 |
| SSE (PRESS_c_) | the smaller the better | 4.3730 |
| MSE (residual) | the smaller the better | 0.1620 |
| df_total_ (n-1) | the bigger the better | 32 |
| SST (total) | the smaller the better | 29.4304 |
| SEE (RMSE_C_) | the smaller the better | 0.4024 |
| MAE_C_ (100% data) | the smaller the better | 0.3009 |
| MAE_C ;_ RMSE_C_ | the smaller the better RMSE_C_≅ RMSE_LOO_≅ RMSE_P_ | RMSE_C_$>$MAE_C_ |
| F(df) Statistics | > f (critical value) | 30.9352 (5;27)  f (critical value): 2.3E-10 |
| Q^2^_(LOO)_ | > 0.6 | 0.7809 |
| $\left\vert R^{2}-\text{Q}\text{2}\text{(LOO)} \right\vert$ | < 0.1 | 0.0705 |
| RMSE_LOO_ | the smaller the better RMSE_C_≅ RMSE_LOO_≅ RMSE_P_ | 0.4421 |
| $\overline{r}_{m (LOO)}^{2}$ | > 0.5 | 0.7019 |
| ${\Delta r}_{m (LOO)}^{2}$ | < 0.2 | 0.0931 |
| MAE_LOO_ (100% data) | the smaller the better | 0.3685 |
| MAE_LOO_ (95% data) | $MAE\leq0.1\cdot TSR$ and $MAE+3\sigma\leq0.2\cdot TSR$  $MAE>0.15\cdot TSR$ or $MAE+3\sigma>0.25\cdot TSR$ | **Moderate** |
| $\overline{R}_{Y-rand}$ | << r | 0.4028 |
| $\overline{R}_{Y-rand}^{2}$ | << R^2^ | 0.1623 |
| $\overline{Q}_{Y-rand}^{2}$ | << 0.6 | -0.2821 |
| **External validation** (test set) **(n=14)** | | |
| $R_{pred}^{2}$ | > 0.6 | 0.8410 |
| $R_{0}^{2}$ | ≈ R^2^ | 0.8329 |
| $R_{0}^{'2}$ | ≈ R^2^ | 0.7585 |
| RMSE_P_ | the smaller the better; RMSE_C_≅ RMSE_LOO_≅ RMSE_P_ | 0.4908 |
| $Q_{F1}^{2}$ | > 0.5 | 0.8309 |
| $Q_{F2}^{2}$ | > 0.5 | 0.8120 |
| $Q_{F3}^{2}$ | > 0.5 | 0.7299 |
| $\overline{r}_{m (test)}^{2}$ | > 0.5 | 0.7268 |
| ${\Delta r}_{m (test)}^{2}$ | < 0.2 | 0.1392 |
| MAE_val_  (100% data) | the smaller the better | 0.4697 |
| MAE_test_ (95% data) | $MAE\leq0.1\cdot TSR$ and $MAE+3\sigma\leq0.2\cdot TSR$  $MAE>0.15\cdot TSR$ or $MAE+3\sigma>0.25\cdot TSR$ | **Moderate** |
| $\frac{R^{2}-R_{0}^{2}}{R^{2}}$ | < 0.1 | 0.0097 |
| $\frac{R^{2}-R_{0}^{'2}}{R^{2}}$ | < 0.1 | 0.0981 |
| k | 0.85 ≤ k ≤ 1.15 | 1.0332 |
| k’ | 0.85 ≤ k’ ≤ 1.15 | 0.9600 |
| $\left\vert R_{0}^{2}-R_{0}^{'2} \right\vert$ | < 0.3 | 0,0744 |
| CCC_test_ | 0.8500 | 0.8866 |
| $\left\vert R^{2}-Q_{F1}^{2} \right\vert$ | $\leq0.1$ | 0.0205 |
| $\left\vert R^{2}-Q_{F2}^{2} \right\vert$ | $\leq0.1$ | 0,0394 |
| $\left\vert R^{2}-Q_{F3}^{2} \right\vert$ | $\leq0.1$ | 0.1215 |
| **Applicability Domain - Williams plot**  (training and test set) | | |
| $h=\frac{3\cdot p}{n}$ | < h | <0.4545 |
| standardized residuals | ± 3 | ± 3 (Fig. 3) |

The R^2^ coefficient indicates that 85.14% of the pMIC variation is explained by the QSAR model. The minimal difference between the classic R2 coefficient and its adjusted version (R^2^_adj_) suggests the absence of redundant molecular descriptors of low importance. The model meets the requirement of at least five chemical compounds per molecular descriptor, and no significant collinearity was detected among the explanatory variables (r<0.3611, as shown in Table S4).

**Table S4**. Pearson's linear correlation coefficients (r) of molecular descriptors.

|  | RDF100e | RDF120s | I_TH_ | $R_{4}^{+}\left( m \right)$ | GATS8e |
| --- | --- | --- | --- | --- | --- |
| RDF100e | 1 |  |  |  |  |
| RDF120s | 0.1087 | 1 |  |  |  |
| I_TH_ | 0.3182 | 0.3611 | 1 |  |  |
| $R_{4}^{+}\left( m \right)$ | -0.1428 | -0.1391 | 0.0878 | 1 |  |
| GATS8e | 0.2920 | 0.2478 | 0.0923 | -0.1438 | 1 |

Residuals normality was assessed using a quantile-quantile (Q-Q) plot, where the aligment of observed residues along a straight line confirmed their normal distribution. The variance of the error is crucial for coefficient inference and quality of prediction. Therefore, the ANalysis Of Variance (ANOVA) was used to decompose the variance of Y into regression and the error components. The sum of squared error (SSE) between experimental and predicted pMIC values, was found to be 4.3730 and is also known as PRESSC of the training set in literature. The SST value is significantly higher than SSE, indicating that variability explained by the regression (SSR) is much higher than unexplained variability, known as error. The average squared distance between observed and predicted pMIC values has been determined as 0.1620 through the calculation of Mean Square Error (MSE). Furthermore, the Root Mean Squared Error (RMSE) was calculated, which differs from the MSE as it employs natural data units (mol/l) instead of square units. RMSEC, which is the average difference between the observed (measured experimentally) pMIC, and the predicted values by the QSAR model in the training set, was found to be 0.4024. The RMSEC value is slightly higher than the Mean Absolute Error (MAEC), indicating the low probability of occurrence of large error values in the training set. An approximate 95 % confidence interval for predicting future data is $\hat{y}\pm2RMSE$ i.e., $\hat{y}$±0,8048. This statement holds true if the model is correct and if model errors made follow a normal distribution, as will be demonstrated in the subsequent sections of this article [58]. The significance of the entire QSAR model was assessed using the F-statistic. In other words, it was tested whether the model as a whole (including all predictor variables) explains a significant portion of the variation in the dependent variable compared to the model without predictors. As the F-statistics of 30.9352 is greater than the critical value of 2,3E-10, it means that there’s statistical evidence for rejecting the null hypothesis that the value of all regression coefficients is 0. It can be concluded that the antifungal activity of thiazole derivatives towards *Candida albicans* (ATCC 2091) is significantly related to molecular features encoded by RDF100e, I_TH_, $R_{4}^{+}\left( m \right)$, RDF120s, GATS8e.

It's important to note that the goodness-of-fit parameters (R^2^, RMSE) can be misleading in MLR equations. Specifically, these parameters tend to overestimate the accuracy of models on smaller sample sizes compared to larger ones (RMSE depends on the scale of measure). This is a classic example of the bias-variance trade-off [59]. Therefore, we conducted both internal (training set) and external validation (test set), in addition to the standard goodness of fit assessment. At internal validation, the robustness of the model was evaluated with the data resampling technique: Leave-One-Out Cross-Validation (LOO-CV). Király et al. have pointed out that the use of Leave-One-Out scheme instead of the Leave Many-Out one in the linear models may not increase the uncertainty of the assessment [59]. The DTC-QSAR tool (version 1.0.5) does not allow for external validation using the leave-many-out cross-validation technique. The validation results confirmed that the model has good robustness (Q^2^_(LOO)_ = 0.7809) and stability ($\left| R^{2}-\text{Q}\text{2}\text{(LOO)} \right|$= 0.0705). As the difference between R^2^ and Q^2^_(LOO)_ is significantly less than the critical value of 0.1, then it can be concluded that the model is not affected by overfitting [60,61]. According to Chirico and Gramatica, the verification of any QSAR model for internal quality (R^2^ > 0.7, Q^2^_(LOO)_ > 0.6, |R^2^ - Q^2^_(LOO)_|<0.1) is a necessary but not sufficient condition [62]. Therefore, $\overline{r}_{m (LOO)}^{2}$ and $\overline{r}_{m (test)}^{2}$ validation parameters were calculated, which could be considered more rigorous metrics than Q^2^ and $R_{pred}^{2}$, respectively [63,64]. It needs to be highlighted that, the $\overline{r}_{m}^{2}$ is a rigorous metric only for the QSAR models when the bias in the external data is the scale shift type with negative angles, while for positive angles, it depends on the applied thresholds [62]. Following Kunal Roy's recommendations, it can be concluded that the QSAR model has a satisfactory predictive performance as the values of $\overline{r}_{m (LOO)}^{2}$ and $\overline{r}_{m (test)}^{2}$ in the model are 0.7019 and 0.7268 respectively, which are higher than the critical value of 0.5. In addition, the values of ${\Delta r}_{m (LOO)}^{2}$ and ${\Delta r}_{m (test)}^{2}$are well below the critical value of 0.2, which suggests that the model's predictive abilities are acceptable. Based on the analysis, there seems to be no scale shift error in the model when negative angles are considered. However, $\overline{r}_{m}^{2}$ and ${\Delta r}_{m}^{2}$ metrics are unable to discriminate among the various applied angles for the location and scale shift bias ($\overline{r}_{m}^{2}=1 and {\Delta r}_{m}^{2}=0$ whatever the applied angle). As a result, these metrics overlook the essential requirement for predictive models to have either k or k' close to 1,1 respectively [62]. According to Gramatica's recommendations, the concordance correlation coefficient (CCC) of Lin was also calculated, which performs better in comparison to to $\overline{r}_{m}^{2}$, ${\Delta r}_{m}^{2}$ in location plus scale shifts bias type [62]. The coefficient measures the precision of observations in relation to the fitting line, as well as the accuracy of the regression line in comparison to a line with a slope of 1 passing through the origin, which is known as the concordance line [62]. The CCC value of 0.8866 is greater than the threshold of 0.85 [62]. It is important to note that the developed QSAR model satisfies the $Q_{F2}^{2}$ and $\overline{r}_{m}^{2}$ stricter threshold values of 0.70 and 0.65, respectively [62], instead of the commonly used values ($Q_{F2}^{2}$ = 0.6 and $\overline{r}_{m}^{2}$= 0.5)

Roy et al. have pointed out that conventional correlation-based external validation metrics, such as $Q_{F1}^{2}$ and $Q_{F2}^{2}$, can result in a biased assessment of model predictivity [65]. Therefore, MAE criteria and the corresponding standard deviation (σ) measure of the predicted residuals were adopted to assess the external prediction of the model. Model-derived predictions were found to satisfy the criteria for “moderate predictions” based on MAE-based metrics (MAE < 0.1 × training set range, but MAE + 3 × σ value is between 0.20 × training set range and 0.25 × training set range)) measured after omitting 5% of data points with high prediction residuals.

According to Gramatica, there are rare cases, mainly in small datasets, where the model can be considered acceptable based on the CCC metric, while in the sense of the Golbraikh and Tropsha criteria (G&T), this model should be rejected as non-predictive [62]. Taking this into consideration, the metrics proposed by Golbraikh and Tropsha were calculated ($\frac{R^{2}-R_{0}^{2}}{R^{2}}$, $\frac{R^{2}-R_{0}^{'2}}{R^{2}}$, k, k’). The $\frac{R^{2}-R_{0}^{2}}{R^{2}}$ is 0.0097, which indicates that the determination coefficient value of regression through the origin ($R_{0}^{2}$) is close enough to R2 of the unconstrained model. The slopes k and k' are 1.0332 and 0.9600 respectively, satisfying the criteria of 0,85 ≤ k (k') ≤ 1,15, which indicates that the experimental and predicted values will be aligned along the diagonal line on a scatter plot.

An important next step in the validation process is performing Y-randomization. This method helps to assess the robustness and reliability of the QSAR models by ensuring that the observed correlations are not due to chance. The Y-randomization test was used to check if experimental pMIC values were correlated with molecular descriptors by chance. Y-randomization involves scrambling the Y-column data while keeping the descriptor matrix (X-matrix) unchanged. Model randomization, Y-scrambling is performed with the molecular descriptors present in the developed QSAR model. The models are built using the scrambled data, and the values of determination coefficients ($R_{Y-rand}^{2}$; $Q_{Y-rand}^{2}$) are calculated. To establish if the actual model is based on random correlation, a straightforward method created by Eriksson and Wold was employed. This method comprises a series of decision inequalities that rely on the values of $R_{Y-rand}^{2}$ and $Q_{Y-rand}^{2}$, as well as their relationship with each other [66]. The methodology for assessing Y-randomization, as proposed by Wold and Eriksson, is detailed in Table S5.

**Table S5.** Methodology for evaluating Y-randomization results.

| Inequality | Decision |
| --- | --- |
| $Q_{Y-rand}^{2}<0.2 and R_{Y-rand}^{2}<0.2$ | no chance correlation |
| $any Q_{Y-rand}^{2} and 0.2<R_{Y-rand}^{2}<0.3$ | negligible chance correlation |
| $any Q_{Y-rand}^{2} and 0.3<R_{Y-rand}^{2}<0.4$ | tolerable chance correlation |
| $Q_{Y-rand}^{2}<0.2 and R_{Y-rand}^{2}>0.4$ | recognized chance correlation |

Additionally, Figure S1 presents the distribution of $R_{Y-rand}^{2}$ and $Q_{Y-rand}^{2}$ values after 200 times randomly shuffling the biological response for compounds.

**Figure S1.** $R_{train}^{2}$ and $Q_{LOO}^{2}$ values after 200 Y-randomization tests for genetic algorithm-multiple linear regression (GA-MLR).

In most iterations, the value of $Q_{Y-rand}^{2}$ is less than zero and the value of $R_{Y-rand}^{2}$ is not greater than 0.3, which means that there is a negligible chance (probability) of obtaining a good fit with randomly reorganized response data. Since the values of $\overline{R}_{Y-rand}^{2}$ and $\overline{Q}_{Y-rand}^{2}$ in the obtained QSAR models are 0.1623 and -0.2821 respectively, which are lower than R^2^ and Q^2^_(LOO)_ of the non-randomized (original) model, it can be concluded that experimental pMIC values are correlated with the molecular descriptors, not coincidentally.

An essential next step in the validation of a QSAR model, as recommended by the OECD guidelines, is the determination of the model's applicability domain. Even the most reliable QSAR model cannot accurately predict the modeled property for all chemicals in the universe. In fact, only predictions for chemicals that fall within this specific domain can be deemed reliable. The Williams plot (Fig. S2) was used to determine the domain of the AD model, with leverage values being placed on the X-axis and standardized residuals on the Y-axis. The Williams plot is an immediate and simple graphical detection of both the response outliers (i.e., compounds with standardized residuals greater than three standard deviation units, >3s) and structurally influential chemicals in a model (h>h*).

**Figure S2.** Williams plot of applicability domain of the QSAR model for antifungal activities against Candida albicans (ATCC 2091) of thiazole derivatives (training set – blue points, test set – magenta points).

Three compounds (G3_3n, G3_3g, and G3_3l) were excluded based on statistical diagnostics during model construction. Specifically, G3_3n and G3_3g were identified as outliers based on the Williams plot analysis used to assess the applicability domain. Their standardized residuals exceeded acceptable thresholds. Compound G3_3l, although not an outlier in terms of residuals, exhibited a leverage value above the critical threshold (h), indicating disproportionate influence on the regression model. The final QSAR model was developed without these compounds. The G1_3j compound was structurally influential in a model, i.e., the small residue and leverage value exceeding the critical h* value of 0,4545. This compound stabilized the model and improved its accuracy.

All obtained validation metrics suggest that the derived model is robust and statistically significant for predicting the antifungal activity of thiazole derivatives towards *Candida albicans* ATCC 2091.

1. The Calculation Algorithms of Selected Molecular Descriptors

Molecular descriptors are a crucial aspect of QSAR models, as they provide valuable information about the properties and behavior of molecules. The type of molecular descriptors used depends on the chosen molecular representation and the algorithm used to calculate them. In a subsequent part of the article, we provided on an overview of the calculation methodology of the molecular descriptors: RDF100e (section 4.1), I_TH_ (section 4.2), $R_{4}^{+}\left( m \right)$ (section 4.3), RDF120s (section 4.1), GATS8e (section 4.4).

- 1. Radial distribution function - RDF100e and RDF120s

The radial distribution function, RDF(R), is a class of 3D molecular descriptors to analyze the internal structure of compounds. The RDF denoted also as g(R) is defined as:

| $RDF\left( R \right)=f\cdot\sum_{i=1}^{A-1} \sum_{j=i+1}^{A} w_{i}\cdot w_{j}\cdot e^{-\beta\cdot\left( R-r_{ij} \right)^{2}},$ | (S1) |
| --- | --- |

where:

f – scaling factor,

A – number of the atoms (hydrogens included),

$w_{i},w_{j}$ – the atomic property of the *i*th and *j*th atoms,

β – smoothing term, which defines the probability of the individual interatomic distances,

R – spherical volume of radius R,

$r_{ij}$ – interatomic distances between the *i*th and *j*th atoms.

The RDF estimates the probability of finding two atoms (*i* and *j)* within a spherical shell with radius R, weighted by physicochemical property. The QSAR equation supplies information on what geometrical distance (R) is relevant to the biological activity under study. Two types of description are necessary for the numerical representation of molecular structure: physicochemical and geometric (3D). A weighting scheme is used to assign a numerical value of the physicochemical property ($w_{i},w_{j}$) to each atom of the molecule. In RDF100e, "e" means electronegativity, while "s" in RDF120s denotes the use of I-State as a weighting scheme. The geometric description of the molecule uses the molecular matrix (M) of the Cartesian coordinates (x, y, z) calculated on the H-filled three-dimensional molecular graph for a defined conformation:

| $M=\left[ \begin{matrix} x_{i} & y_{i} & z_{i} \\ x_{j} & y_{j} & z_{j} \\ \cdots& \cdots& \cdots\\ x_{A} & y_{A} & z_{A} \end{matrix} \right],$ | (S2) |
| --- | --- |

where:

$x_{i}, y_{i}, z_{i}$ – position of *i*th atom in Cartesian x,y,z coordinates,

$x_{j}, y_{j}, z_{j}$ – position of *j*th atom in Cartesian x,y,z coordinates,

A – number of the atoms (hydrogens included).

Next, the Euclidean distances between every pair of atoms *i* and *j* in the molecule ($r_{ij}$) are calculated based on M matrix using Equation S3:

| $r_{ij}=\sqrt{\left( x_{i}-x_{j} \right)^{2}+\left( y_{i}-y_{i} \right)^{2}+\left( z_{i}-z_{j} \right)^{2}}.$ | (S3) |
| --- | --- |

In RDF(R), the R-value encodes the distance between atoms in the molecule, at which the probability of finding two atoms within a spherical shell is evaluated. The R-value is 10-fold of the distance ($r_{ij}$); for example the distance for RDF100e is 10 Å, whereas for RDF120s it is 12 Å. The elementary unit of RDF100e and RDF120s is atomic pair contribution ($\gamma_{ij}$), which is given by Equation S4:

| $\gamma_{ij}=w_{i}\cdot w_{j}\cdot e^{-\beta\cdot\left( R-r_{ij} \right)^{2}}.$ | (S4) |
| --- | --- |

The value of $\gamma_{ij}$ is influenced by two factors, namely the electronegativity (RDF100e) of the atoms or their I-State (RDF120s), and the geometry distance between them ($r_{ij}$). The contribution of a given atomic pair to the descriptor value increases with a smaller difference between interatomic distance ($r_{ij}$) and R value and a higher product of atomic properties ($w_{i}\cdot w_{j}$). Parameters f and β have a constant value and do not affect interpretation. The significance of atomic pairs in explaining the relationship between descriptor value and activity under study can be analyzed by the distribution of contributions $\gamma_{ij}$, allowing them to be arranged in decreasing order of $\gamma_{ij}$.

- 1. The total and standardized information content on the leverage equality - I_TH_

The total and standardized information content on the leverage equality (I_TH_) belongs to the class of GEometry, Topology, and Atom-Weights AssemblY (GETAWAY) descriptors. The ITH has a form:

| $I_{TH}=A_{0}\cdot{log}_{2}A_{0}-\sum_{g=1}^{G} N_{g}\cdot{log}_{2}N_{g},$ | (S5) |
| --- | --- |

where:

$A_{0}$ – the number of non-hydrogen atoms,

$N_{g}$ – the number of atoms with the same leverage value,

G – the number of equivalence classes.

In contrast to RDF(R), I_TH_ encodes the molecular structure only geometrically using concept of leverages ($h_{ii}$), without including the physicochemical properties of individual atoms in the calculation methodology. The leverage is a measure of distance between centered cartesian coordinates for the *i*th atom and the mean of the centered Cartesian coordinates for all atoms of molecule (M, molecular matrix). Thus, the atoms situated on the periphery of the molecule have the greatest leverage values. My previous article includes a thorough examination of the leverage concept in relation to descriptor interpretation [67]. The *i*th leverage $h_{ii}$ is the *i*th diagonal element of the Molecular Influence Matrix (MIM) matrix. The MIM, denoted also by H, is defined in terms of centered Cartesian Coordinates (M) as:

| $H=M\cdot\left( M^{T}\cdot M \right)^{-1}\cdot M^{T},$ | (S6) |
| --- | --- |

where:

M – matrix of centered Cartesian atomic coordinates x, y, z,

T – the superscript refers to the transposed matrix.

I_TH_ contains two terms, $A_{0}\cdot{log}_{2}A_{0}$ and $\sum_{g=1}^{G} N_{g}\cdot{log}_{2}N_{g}$. The first one can be interpreted as a state of disorder of molecules (entropy), i.e., the arrangement of atoms in space. The $\sum_{g=1}^{G} N_{g}\cdot{log}_{2}N_{g}$ term, on the other hand, informs molecular symmetry. The term $\sum_{g=1}^{G} N_{g}\cdot{log}_{2}N_{g}$ requires the identification of atoms with the same leverage value ($h_{ii}-h_{jj}\cong0$) and grouping them into classes according to their leverage value. The value of Ng corresponds to the total count of atoms assigned to a specific class. A class may contain two or more atoms with matching leverage values (${log}_{10}1=0$). Hence, the interpretation of I_TH_ is based on analyzing sets of atoms, rather than atomic pairs as in RDF(R). If each atom is located at a different distance from the molecule center (i.e., has different values of $h_{ii}$), term $\sum_{g=1}^{G} N_{g}\cdot{log}_{2}N_{g}$ equals zero and I_TH_ is calculated with formula $A_{0}\cdot{log}_{2}A_{0}$. However, for molecules whose all atoms have the same leverage value (a perfectly symmetric theoretical case), the I_TH_ value is 0.

- 1. R maximal autocorrelation of lag 4 / weighted by mass - $\boldsymbol{R}_{\mathbf{4}}^{\mathbf{+}}\left( \boldsymbol{m} \right)$

As well as I_TH_, the R maximal autocorrelation of lag 4 / weighted by mass ($R_{4}^{+}\left( m \right)$) belongs to the autocorrelation class of GEometry, Topology, and Atom-Weight s AssemblY (GETAWAY) descriptors. $R_{4}^{+}\left( m \right)$ is a special case of the descriptor $R_{4}(m)$. The autocorrelation functions of $R_{4}(m)$ and $R_{4}^{+}\left( m \right)$ are defined by:

| $R_{k}\left( w \right)=\sum_{i=1}^{A-1} \sum_{j>i} \frac{\sqrt{h_{ii}\cdot h_{jj}}}{r_{ij}}\cdot w_{i}\cdot w_{j}\cdot\delta(k;d_{ij}) i\neq j and k=1,2,\ldots, d,$ | (S7) |
| --- | --- |
| $R_{k}^{+}\left( w \right)={max}_{ij}\left( \frac{\sqrt{h_{ii}\cdot h_{jj}}}{r_{ij}}\cdot w_{i}\cdot w_{j}\cdot\delta\left( k;d_{ij} \right) \right) i\neq j and k=1,2,\ldots, d$, | (S8) |

where:

A – number of the atoms (hydrogens included),

$h_{ii}$, $h_{jj}$ – the leverage values of *i*th and *j*th atom,

$w_{i},w_{j}$ – the atomic property of the *i*th and *j*th atom,

$r_{ij}$ – Euclidean (geometric) distance between *i*th and *j*th atom,

$\delta\left( k;d_{ij} \right)$– Dirac delta function,

$d_{ij}$ – topological distance between *i*th and *j*th atom,

k – considered lag 1, 2,…, d.

Similarly, to RDF(R), the descriptor $R_{4}(m)$ exhibits an additive character. Therefore, the elementary unit of the $R_{4}(m)$, and thus $R_{4}^{+}\left( m \right)$, is the atomic pair contribution ($\gamma_{ij}$) whose value depends on the atomic properties ($w_{i},w_{j}$_j_), leverages ($h_{ii}$, $h_{jj}$), the geometric ($r_{ij}$) and topological distance ($d_{ij}$) by which these atoms are spaced apart. The elementary unit of $R_{4}(m)$ and $R_{4}(m)$ is atomic pair contribution ($\gamma_{ij}$), which is given by Equation 9:

| $\gamma_{ij}=\frac{\sqrt{h_{ii}\cdot h_{jj}}}{r_{ij}}\cdot w_{i}\cdot w_{j}\cdot\delta\left( k;d_{ij} \right) i\neq j and k=1,2,\ldots, d.$ | (S9) |
| --- | --- |

Upon analyzing the Eq. S7 and Eq. S8, it has been determined that the value of $R_{4}^{+}\left( m \right)$ is derived from the atomic pair that has the highest contribution of $\gamma_{ij}$ towards the value of $R_{4}(m)$.

The $R_{4}^{+}\left( m \right)$ combines three ways of describing molecular structure: physicochemical, topological (2D), and geometric (3D). The physicochemical properties of atoms are encoded with the weighting scheme ($w_{i}\cdot w_{j})$. In formula $R_{4}^{+}\left( m \right)$, the letter "m" represents the use of atomic mass as a method of weighting. The geometrical description (3D) of the molecular structure is realized by means of the influence/distance matrix R, which can be expressed as:

| ${[R]}_{ij}=\left[ \frac{\sqrt{h_{ii}\cdot h_{jj}}}{r_{ij}} \right]_{ij} i\neq j,$ | (S10) |
| --- | --- |

where:

$h_{ii}$, $h_{jj}$ – the leverage values of *i*th and *j*th atoms,

$r_{ij}$ – Euclidean (geometric) distance between *i*th and *j*th.

The leverage values are the diagonal elements of the H matrix. In turn, $r_{ij}$ is the Euclidean distance between atoms, read from the distance matrix.

The topological features of molecules are obtained from the representation of molecular structure as a graph, where the vertices correspond to atoms while the edges represent bonds. The topological distance matrix supplies information on the shortest possible path (topological distance) between atoms (vertices), i.e., the minimum number of edges, between the vertices *i* and *j*. The value of k in $R_{k}^{+}\left( w \right)$ indicates the topological distance (lag), which is relevant to the biological activity under study. The Dirac delta function equal to one if the topological distance ($d_{ij}$) is equal to k, respectively zero, otherwise:

| $\delta\left( k;d_{ij} \right)=\left\{ \begin{aligned} 1 if d_{ij}=k, \\ 0 ifd_{ij}\neq k, \end{aligned} \right.$ | (S11) |
| --- | --- |

where:

$d_{ij}$ – the topological distance between the *i*th and *j*th atoms,

k – considered lag 1, 2, …, d.

The Dirac delta function reduces the equation 9 and 10 to zero when k≠4 between atoms *i* and *j*. Therefore, in case $R_{4}(m)$, the descriptor value is calculated based on the contributions of atomic pairs whose atoms are separated by 4 bonds. The contribution of the atomic pair ($\gamma_{ij}$) to the descriptor value increases with a decreasing interatomic distance ($r_{ij}$), greater distance of atoms from the geometric center of the molecule ($h_{ii}$, $h_{jj}$), and increasing product of normalized atomic properties ($w_{i}\cdot w_{j})$.

- 1. Geary autocorrelation of lag 8 weighted by Sanderson electronegativity (GATS8e)

The only two-dimensional (2D) molecular descriptor in the QSAR model is Geary autocorrelation of lag 8 weighted by Sanderson electronegativity (GATS8e). Moreover, it is also known as the Geary coefficient (c(d)), defined as:

| ${GATS}_{k}\left( w \right)=c\left( d \right)=\frac{\frac{1}{2\Delta_{k}}\cdot\sum_{i=1}^{A_{0}} \sum_{j=1}^{A_{0}} {(w_{i}-w_{j})}^{2}\cdot\delta\left( k;d_{ij} \right)}{\frac{1}{A-1}\cdot\sum_{i=1}^{A_{0}} {(w_{i}-\overline{w})}^{2}},$ | (S12) |
| --- | --- |

where:

$w_{i}$_,_ $w_{j}$ – the atomic property of the *i*th and *j*th atoms,

$\overline{w}$ – property mean of the molecule,

$A_{0}$ – the number of non-hydrogen atoms,

$\delta\left( k;d_{ij} \right)$ – Dirac delta function,

$d_{ij}$ – topological distance between i^th^ and j^th^,

k – considered lag 1, 2,…, d,

$\Delta_{k}$– number of atoms (vertex) pairs at distance equal to k.

${GATS}_{k}\left( w \right)$ is a combination of physicochemical and topological (2D) methods for describing the structure of molecules. The physicochemical properties of the atoms are coded by means of the weighting scheme (w_i_, w_j_). In GATS8e, the letter "e" stands for using electronegativity as the weighting method. GATS8e is analogous to the $R_{4}^{+}\left( m \right)$ descriptor in its topological description. In GATS8e, the molecular graph is based on atoms other than hydrogen atoms (H-depleted graph). ${GATS}_{k}\left( w \right)$ is based on a phenomenon called spatial autocorrelation. Regarding the compounds, atomic property at different locations in molecular structure may not be independent. For example, atomic properties at nearby locations may be closer in value than atomic properties at locations farther apart. ${GATS}_{k}\left( w \right)$ is a global tool testing the presence of general clustering (spatial patterns) when atoms with similar values of weights appear in space close to each other (positive autocorrelation). It should be emphasized that space is two-dimensional. When the ${GATS}_{k}\left( w \right)$ value equals zero value, it is interpreted as a strong positive spatial autocorrelation, a larger than 1 value indicates a random spatial pattern. Finally, ${GATS}_{k}\left( w \right)$ value between 0 and 1 means positive autocorrelation.

1. Supporting Tables

**Table S6**. Internal State as a weighting scheme considering electronic properties and atomic topology in the molecule.

| Group | Internal State (s) | Group | Internal State (s) |
| --- | --- | --- | --- |
| H- | 1,00 | =N- | 3,00 |
| >C< | 1,25 | =NH | 5,00 |
| >CH- | 1,33 | -O- | 3,50 |
| -CH2- | 1,50 | ≡N | 6,00 |
| -CH3 | 2,00 | -OH | 6,00 |
| >C= | 1,67 | =O | 7,00 |
| =CH- | 2,00 | -F | 8,00 |
| =CH2 | 3,00 | -Cl | 4,111 |
| >N- | 2,00 | -Br | 2,750 |
| ≡C- | 2,50 | -I | 2,120 |
| -NH- | 2,50 | -S- | 1,833 |
| ≡CH | 4,00 | =S | 3,667 |
| -NH2 | 4,00 |  |  |

**Table S7.** Matrix of squared differences in electronegativity values of atoms $\left( w_{i}-w_{j} \right)^{2}$ from which thiazole derivatives are composed.

|  |  | H | C | S | N | Br | Cl | O | F |
| --- | --- | --- | --- | --- | --- | --- | --- | --- | --- |
|  |  | 0.942 | 1.000 | 1.076 | 1.160 | 1.171 | 1.265 | 1.327 | 1.455 |
| H | 0.942 | 0.000 |  |  |  |  |  |  |  |
| C | 1.000 | 0.003 | 0.000 |  |  |  |  |  |  |
| S | 1.076 | 0.018 | 0.006 | 0.000 |  |  |  |  |  |
| N | 1.160 | 0.048 | 0.026 | 0.007 | 0.000 |  |  |  |  |
| Br | 1.171 | 0.052 | 0.029 | 0.009 | 0.000 | 0.000 |  |  |  |
| Cl | 1.265 | 0.104 | 0.070 | 0.036 | 0.011 | 0.009 | 0.000 |  |  |
| O | 1.327 | 0.148 | 0.107 | 0.063 | 0.028 | 0.024 | 0.004 | 0.000 |  |
| F | 1.455 | 0.263 | 0.207 | 0.144 | 0.087 | 0.081 | 0.036 | 0.016 | 0.000 |

1. Supporting Figures

**Figure S3.** Correlation of raw and standardized RDF100e values. A positive influence of RDF100e on pMIC is observed for RDF100e raw values less than 5.368, while negative influence for RDF100e (raw) values greater than 5.368.

**Figure S4.** Decomposition of thiazole RDF100e values into inter-fragment δ contributions. The compounds are listed in descending order by pMIC values. The division of compounds into activity classes is included. The figure shows raw values for RDF100e. The dashed line indicates the raw descriptor value below which RDF100e positively affects activity ($\beta_{RDF100e}\cdot{RDF100e}_{stand}>0$).

**Figure S5.** Summary of the inter-fragment contributions of thiazole derivatives. The study was limited to molecules with substituted phenyl in the para position as their third fragment. Within each group, compounds were arranged in descending order of pMIC value. The dashed line indicates the raw descriptor value below which RDF100e positively affects activity ($\beta_{RDF100e}\cdot{RDF100e}_{stand}>0$).

**Figure S6.** Analysis of the distribution and significance of the atomic pairs of halogen derivatives of thiazoles. The structures of the compounds under analysis and the numbering of atoms can be found in Figure S7.

| 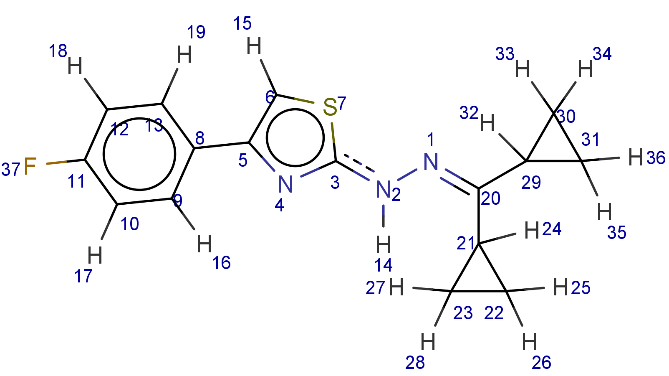 | 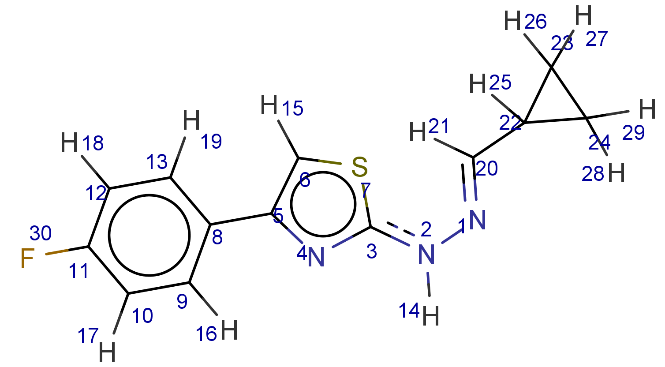 |
| --- | --- |
| G1_3a fluoroderivative of thiazole | G2_3a fluoroderivative of thiazole |
| 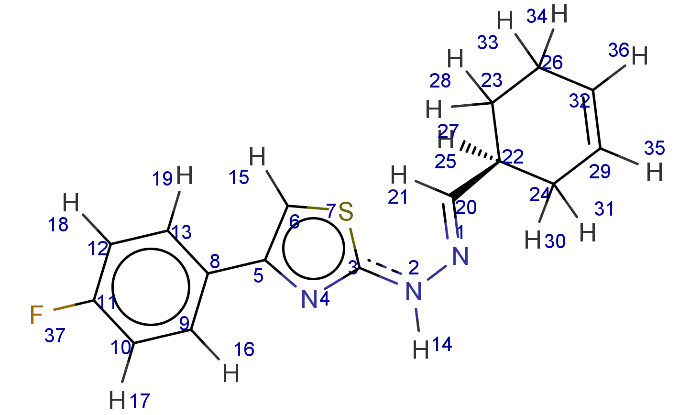 | 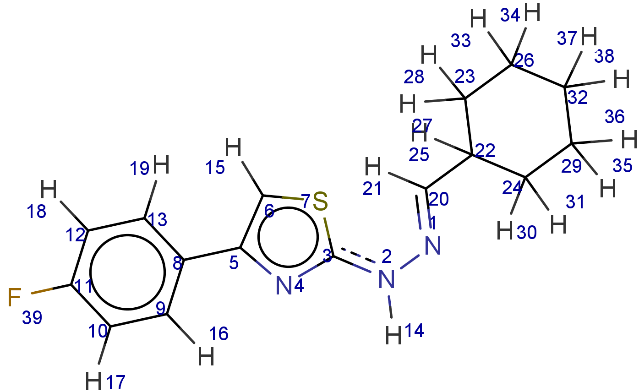 |
| G3_3a fluoroderivatives of thiazole | G4_4b fluoroderivative of thiazole |
| 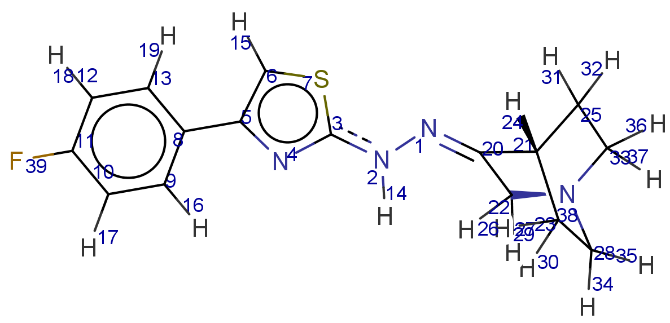 | 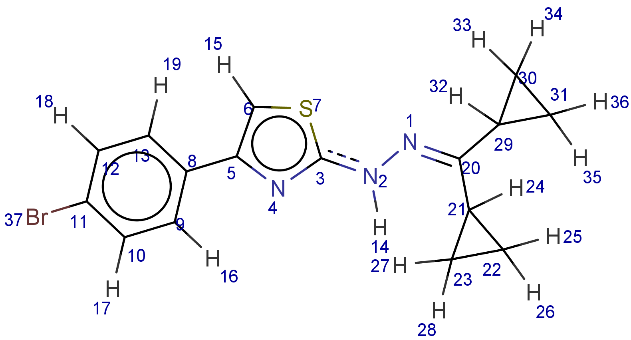 |
| G5_3a fluoroderivativee of thiazole | G1_3c bromoderivative of thiazole |

**Figure S7.** Molecular structures and atom numbering for 5 fluoroderivatives of thiazole and one bromoderivative of thiazole.

**Figure S8.** Cumulative contribution values (Ω) mapped on the accessible surface area (SASA) of halogen derivatives of thiazoles. Important structural fragments are highlighted with red ellipses.

| Group | CH_3_ | CN | NO_2_ |
| --- | --- | --- | --- |
| G1 | 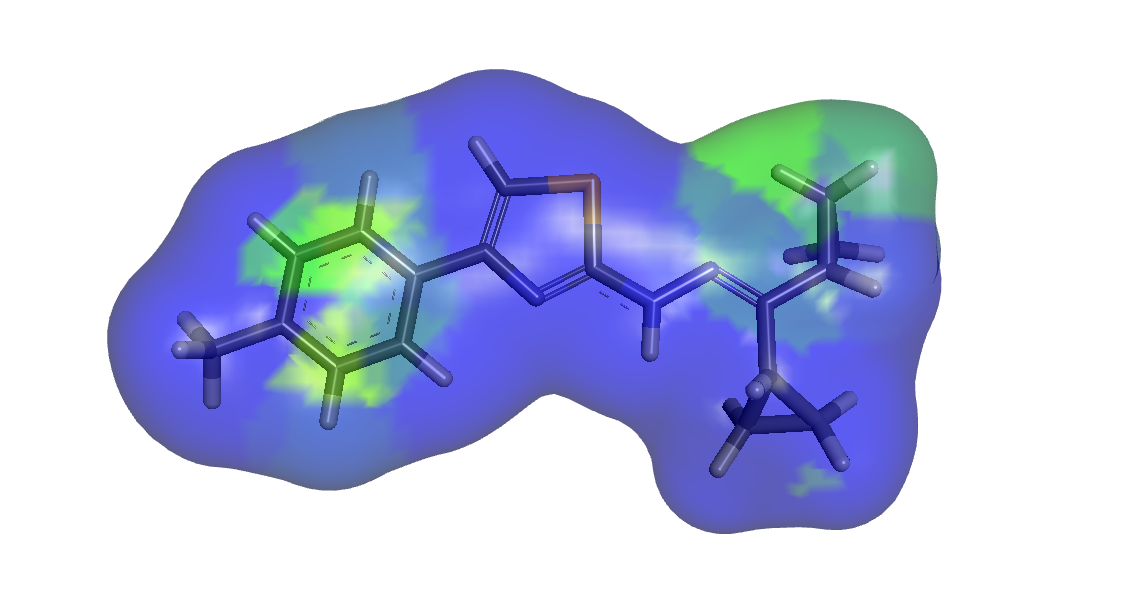 | 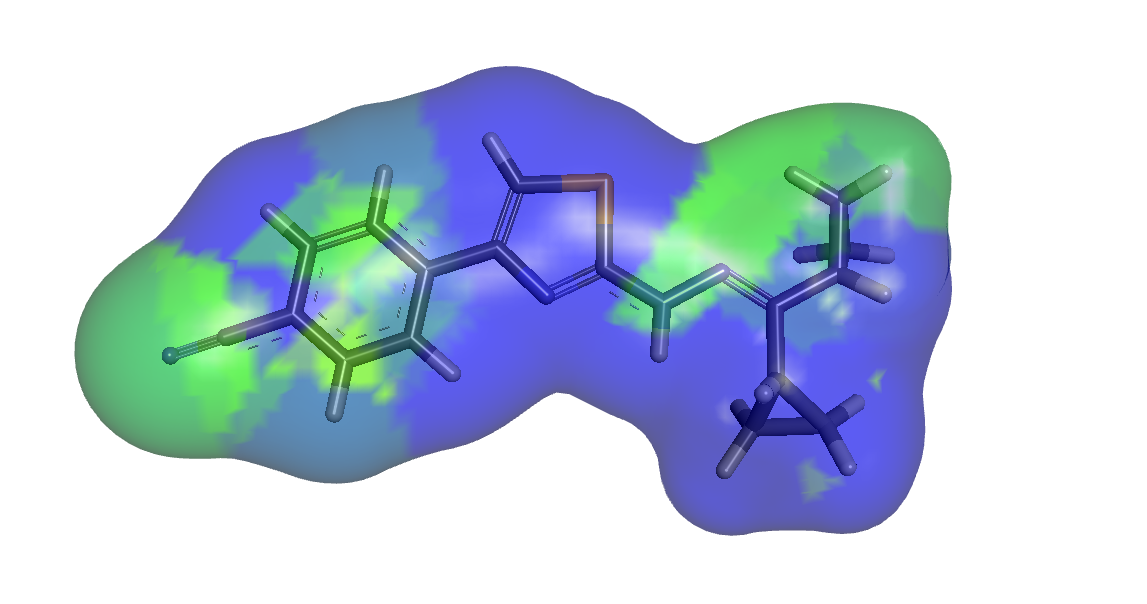 | 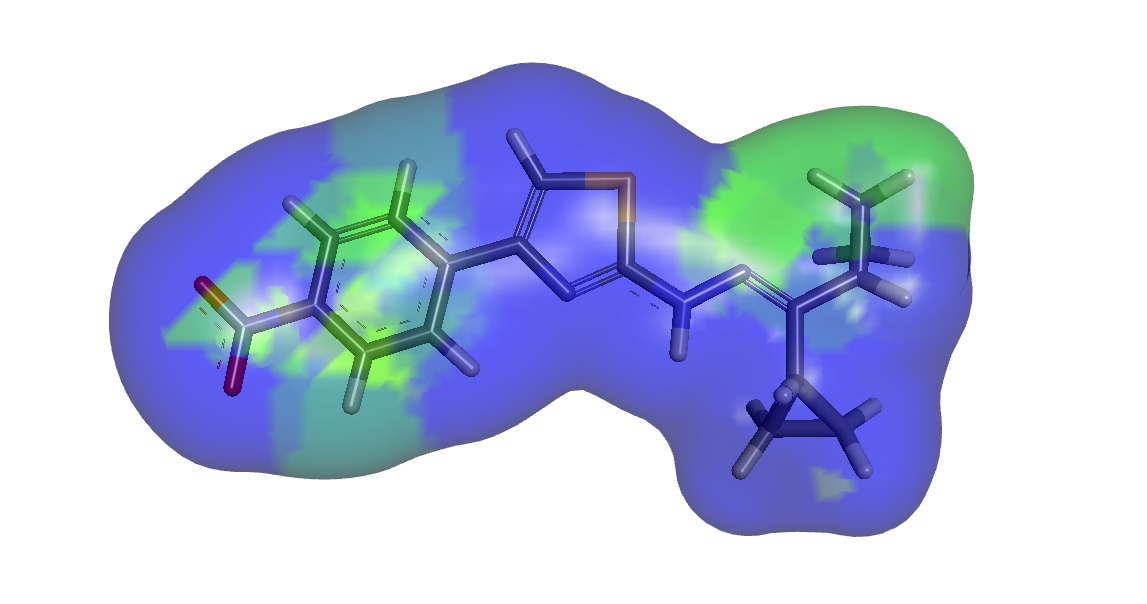 |
|  | G1_3e RDF100e=4.575 | G1_3d RDF100e=5.902 | G1_3h RDF100e=5.270 |
| G2 | 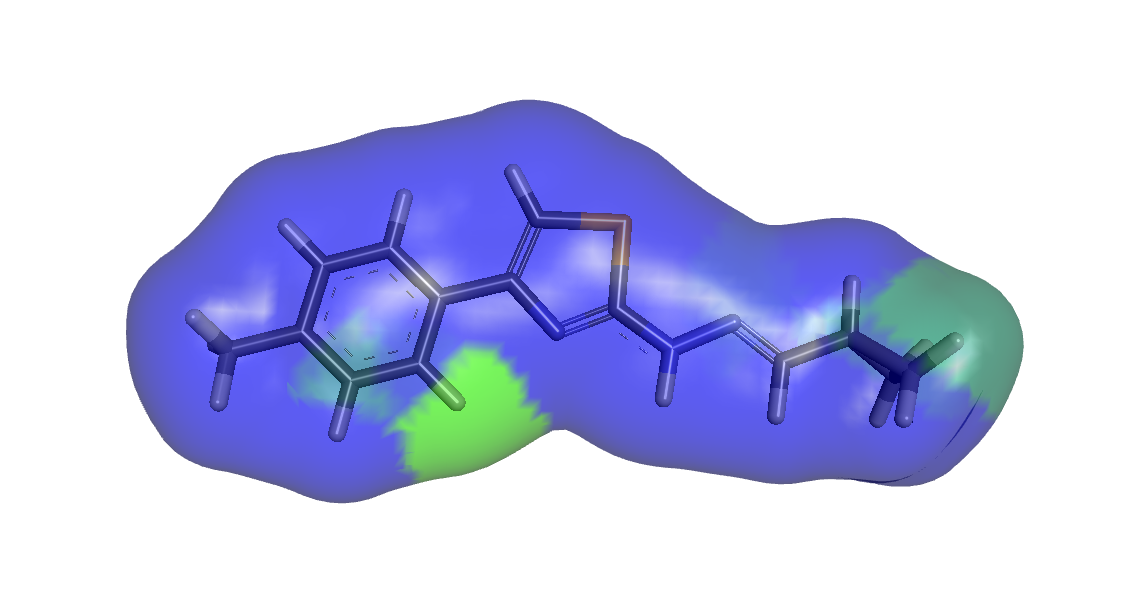 | 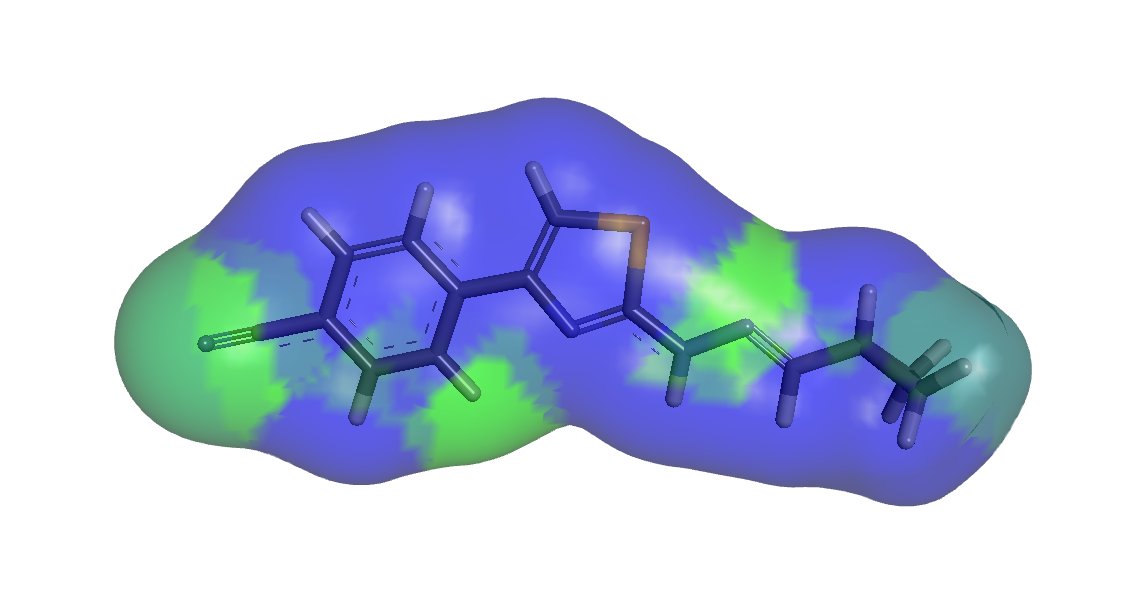 | 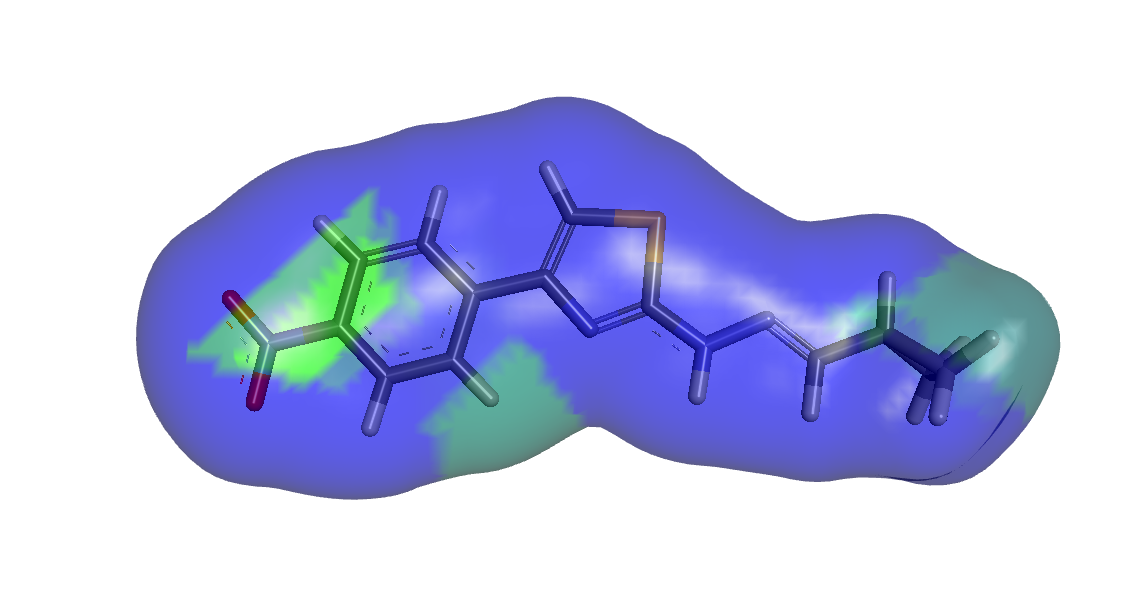 |
|  | G2_3d RDF100e=1.639 | G2_3h RDF100e=3.058 | G2_3j RDF100e=2.402 |
| G3 | 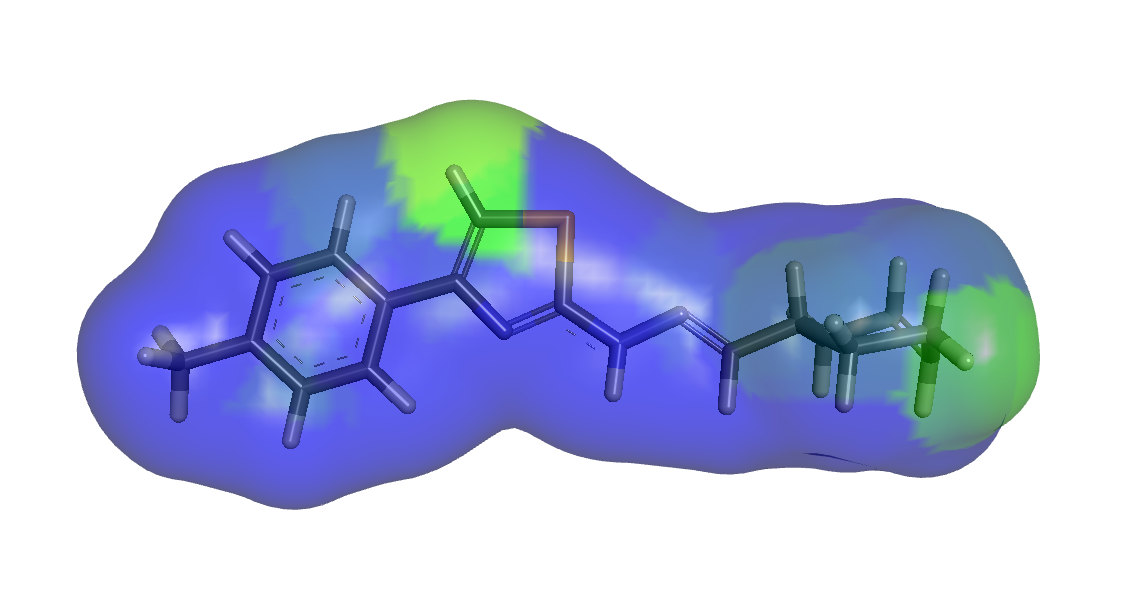 | 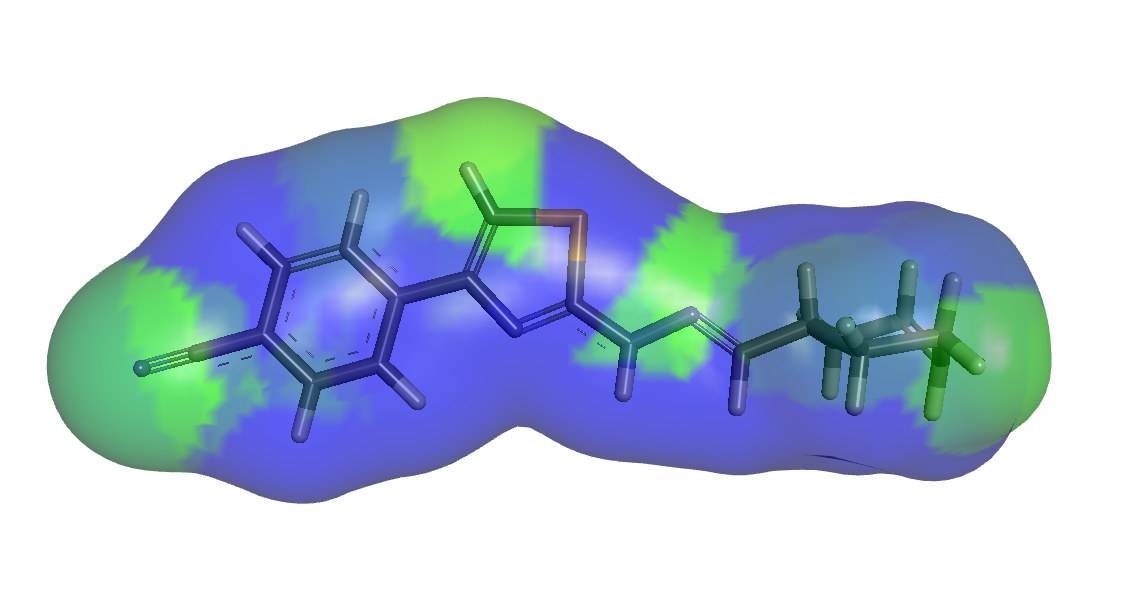 | Molecular structure beyond the model’s applicability domain |
|  | G3_3d RDF100e=3.072 | G3_3h RDF100e=4.525 | G3_3g |
| G4 | 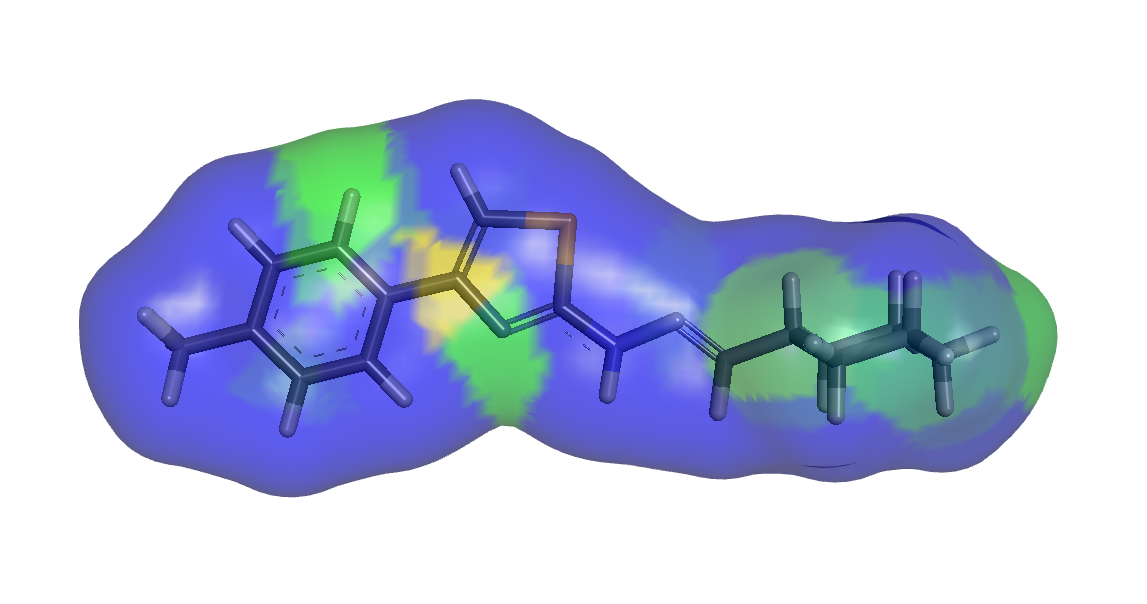 | 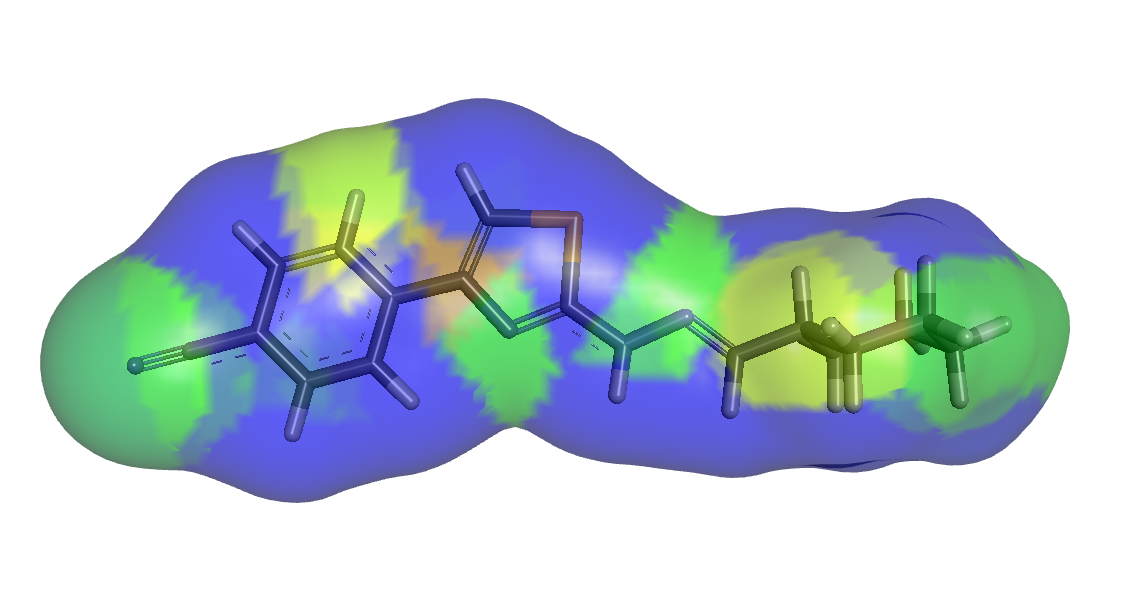 | The molecular structure was not studied |
|  | G4_4h RDF100e=4.872 | G4_4d RDF100e=8.373 |  |
| G5 | 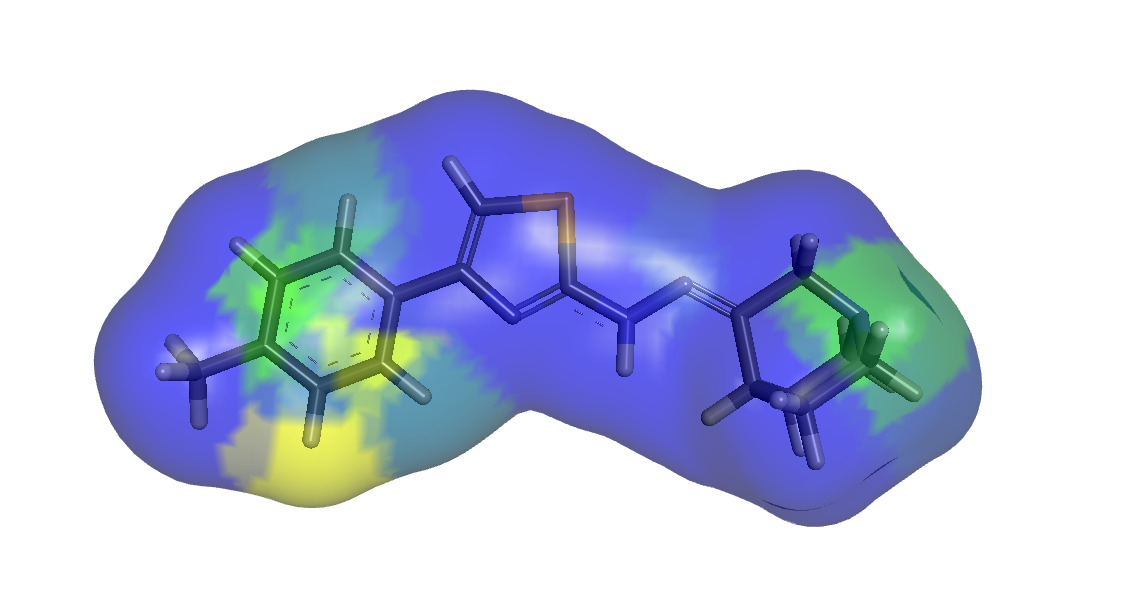 | The molecular structure was not studied | The molecular structure was not studied |
|  | G5_3c RDF100e=6.928 |  |  |

**Figure S9.** Cumulative contribution values (Ω) mapped on the accessible surface area (SASA) of thiazole derivatives (G1-G5) with -CH3, -CN, and -NO2 groups as the R1 substituent of the F3 fragment. Important structural fragments are highlighted with red ellipses

| 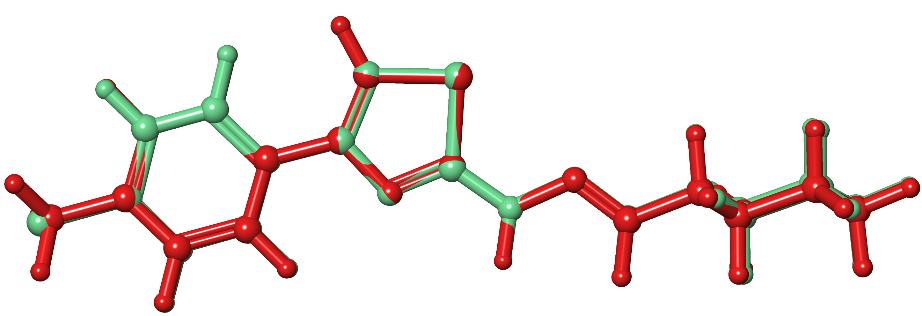 | 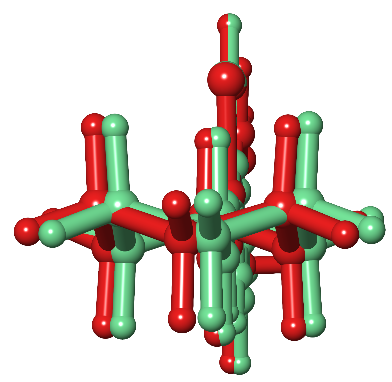 |
| --- | --- |

**Figure S10.** Superposition of the chloroderivative G4_4f and the methylderivative G4_4h. Compound G4_4f is highlighted in green, while G4_4f is highlighted in red.

**Figure S11**. The accessible surface area (SASA) of ten thiazole derivatives with the highest RDF120s values have been mapped with cumulative contribution values (Ω) of their atoms. Additionally, one compound with the lowest descriptor value (G3_3o) has also been included. Important structural fragments have been highlighted with red ellipses for better visibility.

**Figure S12.** Summary of I_TH_ values of thiazole derivatives. The division of compounds into activity classes is included. The compounds have been arranged in descending order based on their pMIC value. The dashed line represents the raw descriptor value, which indicates the positive and negative effects of I_TH_ on the pMIC value. The blue bar represents the descriptor value, while the gray bars measure the ordering of the chemical structure, which is the second part of the descriptor equation: $\sum_{g=1}^{G} N_{g}\cdot{log}_{2}N_{g}$. In turn, the sum of blue and gray bars constitutes the first part of the equation: $A_{0}\cdot{log}_{2}A_{0}$.

**Figure S13.** Visualization of atoms with the same leverage values.

**Figure S14.** List of atomic pairs with the greatest interpretative significance of the $R_{4}^{+}\left( m \right)$ descriptor.

**Figure S15.** Summary of raw GATS8e values for thiazole derivatives. The compounds have been arranged in descending order of pMIC value and grouped into activity classes. A dashed line marks the raw descriptor threshold value (0.872) below which GATS8e has positive effect on activity (pMIC). Compounds with negative spatial autocorrelation (GATS8e>1) are shown with bordered bars along with the structures of their corresponding R1/R2 substituents.

**Figure S16.** Squared differences in electronegativity values $\left( w_{i}-w_{j} \right)^{2}$of atomic pairs with a topological distance of 8 for selected halogen derivatives of thiazoles. The letter X in the name of the atomic pair refers to the type of atom: halogen: F, Cl, or Br. Atomic pairs with large $\left( w_{i}-w_{j} \right)^{2}$ values are highlighted with a red border. The X37-N2 pair is marked with a green filling of the bar.

**Figure S17.** The squares of differences in electronegativity values of the G5_3j compound. Atomic pairs with large $\left( w_{i}-w_{j} \right)^{2}$ values are highlighted with a red border.

1. Supporting Scheme

**Scheme S2.** Techniques for visualizing additive molecular descriptors using RDF100(e) and selected thiazole derivative. The first and second approaches (**plots a** and **b**, respectively) provide a basis for local interpretation of the molecular descriptor. In contrast, the third approach (**plot** **c**) focuses on global interpretation by analyzing SASA-mapped molecular surfaces.

1. References
2. Łączkowski KZ et al. (2018) Synthesis, molecular docking, ctDNA interaction, DFT calculation and evaluation of antiproliferative and anti-Toxoplasma gondii activities of 2,4-diaminotriazine-thiazole derivatives. Med Chem Res 27:1131–1148. https://doi.org/10.1007/s00044-018-2136-6
3. Comesana AE, Huntington TT, Scown CD, Niemeyer KE, Rapp VH (2022) A systematic method for selecting molecular descriptors as features when training models for predicting physiochemical properties. Fuel 321:123836. https://doi.org/10.1016/j.fuel.2022.123836
4. Ballabio D, Consonni V, Mauri A, Claeys-Bruno M, Sergent M, Todeschini R (2014) A novel variable reduction method adapted from space-filling designs. Chemom Intell Lab Syst 136:147–154. https://doi.org/10.1016/j.chemolab.2014.05.010
5. Ambure P, Aher RB, Gajewicz A, Puzyn T, Roy K (2015) “NanoBRIDGES” software: open access tools to perform QSAR and nano-QSAR modeling. Chemom Intell Lab Syst 147:1–13. https://doi.org/10.1016/j.chemolab.2015.07.007
6. Ambure P, Gajewicz-Skretna A, Cordeiro MNDS, Roy K (2019) New workflow for QSAR model development from small data sets: Small Dataset Curator and Small Dataset Modeler. J Chem Inf Model 59:4070–4076. https://doi.org/10.1021/acs.jcim.9b00476
7. Rácz A, Bajusz D, Héberger K (2019) Intercorrelation limits in molecular descriptor preselection for QSAR/QSPR. Mol Inform 38:1800154. https://doi.org/10.1002/minf.201800154
8. Roy K, Das RN, Ambure P, Aher RB (2016) Be aware of error measures: further studies on validation of predictive QSAR models. Chemom Intell Lab Syst 152:18–33. https://doi.org/10.1016/j.chemolab.2016.01.008
9. Roy K, Ambure P (2016) The “double cross-validation” software tool for MLR QSAR model development. Chemom Intell Lab Syst 159:108–126. https://doi.org/10.1016/j.chemolab.2016.10.009
10. Roy K, Ambure P, Kar S (2018) How precise are our quantitative structure–activity relationship derived predictions for new query chemicals? ACS Omega 3:11392–11406. https://doi.org/ 10.1021/acsomega.8b01647
11. Gramatica P, Sangion A (2016) A historical excursus on the statistical validation parameters for QSAR models: a clarification concerning metrics and terminology. J Chem Inf Model 56:1127-1131. https://doi.org/10.1021/acs.jcim.6b00088
12. Golbraikh A, Tropsha A (2002) Beware of q²! J Mol Graph Model 20:269–276. https://doi.org/10.1016/S1093-3263(01)00123-1
13. Ramasami P (2021) Computational Chemistry: Applications and New Technologies. Walter De Gruyter GmbH & Co KG.
14. Alexander DLJ, Tropsha A, Winkler DA (2015) Beware of R²: Simple, unambiguous assessment of the prediction accuracy of QSAR and QSPR models. J Chem Inf Model 55:1316–1322. https://doi.org/10.1021/acs.jcim.5b00206
15. Király P, Kiss R, Kovács D, Ballaj A, Tóth G (2022) The relevance of goodness-of-fit, robustness and prediction validation categories of OECD-QSAR principles with respect to sample size and model type. Mol Inform 41:2200072. https://doi.org/10.1002/minf.202200072
16. OECD (2014) Guidance Document on the Validation of (Quantitative) Structure-Activity Relationship [(Q)SAR] Models. OECD Series on Testing and Assessment No. 69. OECD Publishing. https://doi.org/10.1787/9789264085442-en
17. Eriksson L, Jaworska J, Worth AP, Cronin MTD, McDowell RM, Gramatica P (2003) Methods for reliability and uncertainty assessment and for applicability evaluations of classification- and regression-based QSARs. Environ Health Perspect 111:1361–1375. https://doi.org/10.1289/ehp.5758
18. Chirico N, Gramatica P (2012) Real external predictivity of QSAR models. Part 2: New intercomparable thresholds for different validation criteria and the need for scatter plot inspection. J Chem Inf Model 52:2044–2058. https://doi.org/10.1021/ci300084j
19. Roy PP, Paul S, Mitra I, Roy K (2009) On two novel parameters for validation of predictive QSAR models. Molecules 14:1660–1701. https://doi.org/10.3390/molecules14051660
20. Ojha PK, Mitra I, Das RN, Roy K (2011) Further exploring rm² metrics for validation of QSPR models. Chemom Intell Lab Syst 107:194–205. https://doi.org/10.1016/j.chemolab.2011.03.011
21. De P, Kar S, Ambure P, Roy K (2022) Prediction reliability of QSAR models: An overview of various validation tools. Arch Toxicol 96:1279–1295. https://doi.org/10.1007/s00204-022-03252-y
22. Kiralj R, Ferreira MMC (2009) Basic validation procedures for regression models in QSAR and QSPR studies: Theory and application. J Braz Chem Soc 20:770–787. https://doi.org/10.1590/S0103-50532009000400021
23. Zapadka M, Dekowski P, Kupcewicz B (2022) HATS5m as an example of GETAWAY molecular descriptor in assessing the similarity/diversity of the structural features of 4-thiazolidinone. Int J Mol Sci 23:6576. https://doi.org/10.3390/ijms23126576
